# Cannabinoid receptor expression profiling in lymphocyte subsets reveals clinically relevant immune patterns in SLE

**DOI:** 10.3389/fimmu.2026.1848747

**Published:** 2026-06-03

**Authors:** Angie M. Rosero, Lady J. Rios-Serna, Carlos A. Cañas

**Affiliations:** 1 CIRAT: Centro de Investigación en Reumatología, Autoinmunidad y Medicina Traslacional, Universidad Icesi, Cali, Colombia; 2 Fundación Valle del Lili, Unidad de Reumatología, Cali, Colombia

**Keywords:** cannabinoid receptor, CB1 receptor, CB2 receptor, endocannabinoid system, lymphocytes, systemic lupus erythematosus

## Abstract

**Introduction:**

This study aimed to determine the expression of cannabinoid receptors in lymphocytes from patients with systemic lupus erythematosus (SLE) compared with healthy controls, and to evaluate whether receptor expression is associated with disease activity.

**Methods:**

Using peripheral blood mononuclear cells from 35 SLE patients and 35 healthy controls, we employed multiparametric flow cytometry to characterize T-cell and B-cell subpopulations and their expression of cannabinoid receptors 1 (CB1R) and 2 (CB2R).

**Results:**

Our results reveal a significant imbalance in receptor expression: SLE patients exhibited higher CB2R and lower CB1R levels compared with controls across multiple subsets. Notably, CB1R expression strongly correlated with disease activity, while patients in remission showed expression patterns more similar to healthy individuals. Furthermore, CB2R expression in specific B-cell subsets correlated with clinical manifestations, and its distribution pattern demonstrated a significant ability to distinguish between active disease and remission in ROC analysis.

**Discussion:**

These findings indicate that SLE is characterized by altered cannabinoid receptor profiles in lymphocytes, suggesting that CB1R and CB2R modulation could serve as both a clinical biomarker and a potential therapeutic target for managing the disease.

## 1 Introduction

Systemic lupus erythematosus (SLE) is a systemic autoimmune disease characterized by multisystem involvement and impairment of both innate and adaptive immune responses (1). Its etiopathogenesis reflects an “autoimmunity mosaic,” encompassing genetic, epigenetic, environmental, hormonal, and immunological factors (1, 2). The disruption of self-tolerance leads to a clinical spectrum ranging from mild to severe disease (3, 4).

Despite the availability of targeted therapies that have improved treatment outcomes, critical questions remain regarding mechanisms of treatment resistance and the capacity to achieve sustained remission while minimizing long-term damage (5, 6).

A 2022 systematic review estimated the global incidence of SLE at 5.14 (1.4–15.13) per 100, 000 person-years and a prevalence of 43.7 (15.87–108.92) per 100, 000 persons, with higher rates observed in women (7). Female-to-male ratios between 2:1 and 15:1 have been described in the literature (8).

Altered receptor expression in SLE extends beyond classical immune signaling molecules. Dysregulation of PTPN22 isoforms, steroid hormone receptors (including ERα and androgen receptor balance), vitamin D receptor (VDR), and parathyroid hormone receptor (PTH1R) expression has been reported in patients with SLE (9–15). Collectively, these findings point to broader receptor-level dysregulation involving immune–endocrine pathways. Given the central role of lymphocytes in SLE pathogenesis, exploring immunomodulatory systems that regulate lymphocyte activity, including the endocannabinoid system (ECS), represents a promising research direction (3, 16).

There are several types of cannabinoids, including those present endogenously (endocannabinoids), those isolated from Cannabis sativa (phytocannabinoids), and other synthetic ones (17, 18). These molecules interact with cannabinoid receptors 1 and 2 (CB1R and CB2R), which are G protein-coupled receptors expressed in diverse tissues, with CB2R primarily associated with immune cells (19). While the regulatory role of the ECS in immunity is recognized, its precise contributions remain incompletely understood, with evidence indicating both immunosuppressive and immunostimulatory effects depending on the cannabinoid type and context (17).

This study aimed to determine whether the cannabinoid receptors expression is altered in lymphocytes from SLE patients compared with healthy controls and to evaluate its association with clinical activity y/o particular clinical manifestations. Understanding the dynamics of these receptors can provide insights into aspects related to the pathogenesis of SLE, the development of biomarkers of disease activity, as well as the way for new therapeutic strategies.

## 2 Materials and methods

This cross-sectional study analyzed peripheral blood samples from patients with SLE and healthy controls. PBMCs were isolated and analyzed by multicolor flow cytometry to quantify CB1R and CB2R expression across B- and T-cell subsets. Data preprocessing included quality control, gating, and arcsinh transformation of median fluorescence intensity (MFI) values. Statistical analyses included univariate group comparisons with false discovery rate (FDR) correction, multivariate exploratory analyses (PCA, PLS-DA and MANOVA), and ROC curve analysis to evaluate the discriminatory performance of selected immune cell subsets. Data visualization was performed to support the interpretation of analytical results.

### 2.1 Study design

A cross-sectional observational study was carried out at Universidad Icesi and Fundación Valle del Lili in Cali, Colombia. Sample collection was carried out between 2023 and 2025 and SLE patients’ clinical history was reviewed to describe the demographic data, the relevant clinical manifestations, and disease activity was assessed using the SLE Disease Activity Index (SLEDAI). The study protocol was approved by the appointed ethics committee in biomedical research of Fundación Valle del Lili (protocol number: 1869, act. N°069-2022).

### 2.2 Participants

The study included 35 women diagnosed with SLE according to the “2019 Classification Criteria for Systemic Lupus Erythematosus (EULAR/ACR)” (20) and 35 healthy women without evidence of autoimmune disease, matched by age (± 5 years). All participants were over 18 years and provided written informed consent prior to sample collection. Exclusion criteria included active infections, malignancies, other autoimmune diseases, or recent use of systemic corticosteroids at immunosuppressive doses, due to the risk of significant lymphopenia that may prevent adequate analysis of the data required for the study.

Disease activity was assessed using the SLE Disease Activity Index 2000 (SLEDAI-2K). Patients were classified according to disease activity status as being in remission (SLEDAI-2K = 0) or active disease (SLEDAI-2K ≥1).

### 2.3 Sample collection

Peripheral blood samples were collected in Fundación Valle del Lili by venipuncture under standardized conditions in a 10 mL EDTA tube for each control or patient and samples were transported to Universidad Icesi within the next 2 hours at 4-8 °C for its processing.

### 2.4 Flow cytometry analysis

Peripheral blood samples were processed using 15 mL SepMate™ tubes (StemCell Technologies) to isolate peripheral blood mononuclear cells (PBMCs) via density gradient centrifugation, following the manufacturer’s instructions. After washing, PBMCs were counted using a Neubauer chamber to assess cell viability and adjust the concentration to 1.2 × 10^6^ cells per 100 µL for multicolor flow cytometry panels for B- and T-cell phenotyping.

Panel design and fluorochrome selection were carried out using FluoroFinder panel builder tool. CB1 receptor expression was assessed via indirect staining, using a rabbit anti-CB1 primary antibody (1:10 dilution, Invitrogen by Thermo Fisher Scientific, Waltham, MA, USA) and a goat anti-rabbit IgG secondary antibody (H+L, PerCP conjugated, Novus Biologicals, LLC, USA). CB2 receptor expression was evaluated via direct staining, following conjugation of the rabbit anti-CNR2 (CB2R) primary antibody (Invitrogen by Thermo Fisher Scientific, Waltham, MA, USA) using a CF700 labeling kit (Biotium Inc) following the manufacturer’s instructions. For each assay, 100 µL of PBMCs (1.2 × 10^6^ cells) were used for both the T cell and B cell panels. Data was collected on a CytoFLEX S flow cytometer (Beckman Coulter, Inc., Brea, CA, USA) and analyzed using Cytobank software (BD Biosciences, Ashland, OR, USA).

#### 2.4.1 T cell panel by flow cytometry

PBMCs were incubated with CB1 primary antibody for 15 minutes at room temperature. After washing, cells were stained with the monoclonal antibodies detailed in **Table 1**, and anti-IgG secondary antibody and incubated for 15 minutes at room temperature in the dark. Finally, cells were washed and resuspended for flow cytometry acquisition.

**Table 1:** T cell panel antibodies.

Specificity	Fluorochrome	Clone	Source	Titration
CD45	PC7	J33	Beckman Coulter	1:24
CD3	ECD	UCHT1	Beckman Coulter	1:18
CD4	APC-A750	13B8.2	Beckman Coulter	1:90
CD8	KrO	B9.11	Beckman Coulter	1:26
CD27	APC	1A4CD27	Beckman Coulter	1:24
CD45RA	FITC	ALB11	Beckman Coulter	1:9
CD25^a^	PE	B1.49.9	Beckman Coulter	1:12
CD127^a^	BV605	A019D5	Biolegend	1:51

^a^ Only a subgroup of patients and controls (n = 25) was also incubated with CD25 and CD127.

#### 2.4.2 B cell panel by flow cytometry

PBMCs were incubated with CB1 antibody for 15 minutes at room temperature. After washing, cells were stained using the DuraClone IM B Cell Tube (Beckman Coulter, Inc., Brea, CA, USA) following the manufacturer’s instructions, CB2 CF700-conjugated antibody and anti-IgG secondary antibody were also added and incubated during 15 minutes at room temperature in the dark. Finally, samples were washed and resuspended for flow cytometry acquisition.

### 2.5 Statistical analysis

Initial data analysis was performed using FlowJoTM v.10.10.0 and Cytobank platform (21). Data quality control (QC) was conducted using the PeacoQC tool. The gating strategy included singlet discrimination, lymphocyte selection based on FSC/SSC parameters, and sequential identification of B- and T-cell subsets. Detailed gating schemes are provided in the supplementary material. Fluorescence Minus One (FMO) controls were used to define positive gates for CB1R and CB2R. MFI was used as the quantitative metric for CB1R and CB2R expression across all cell populations (**Supplementary Figure 1**). Representative flow cytometry histograms were generated using samples with median receptor expression values within each study group to illustrate CB2R expression patterns in selected B-cell subsets.

Dimensionality reduction analyses were performed in Cytobank using t-distributed stochastic neighbor embedding (t-SNE; CUDA implementation) with a perplexity of 30 and 750 iterations, and an automatically determined learning rate, to visualize CB1R and CB2R expression across immune cell subpopulations. Concatenated samples from patients and healthy controls were used with equal event sampling.

Statistical analyses were performed in R (v4.5.2). Data preprocessing was conducted using the tidyverse suite of packages, and data visualization was performed using ggplot2, plotly, and ggrepel. MFI values were transformed using an arcsinh transformation with a cofactor of 150, following standard practice in flow cytometry data analysis (22, 23). For laboratory variables, univariate analyses were first conducted. Data normality was assessed using the Shapiro–Wilk test and visual inspection of Q-Q plots to guide the selection of parametric or non-parametric tests. Depending on data distribution, differences in CB1R and CB2R expression between patients and controls were evaluated using Student’s t-test or the Wilcoxon rank-sum test, as appropriate. P-values were adjusted for multiple comparisons using the Benjamini–Hochberg FDR correction across all immune cell subsets and receptor expression comparisons, and an FDR-adjusted p-value < 0.05 was considered statistically significant.

A volcano plot was generated to visualize differential CB1R and CB2R expression across immune cell subsets. Cell subsets meeting significance and effect size thresholds were annotated in the plots.

Multivariate analyses included principal component analysis (PCA) for exploratory data visualization, with results displayed as biplots. Partial least squares discriminant analysis (PLS-DA) was applied as a supervised classification approach to identify variables contributing to group separation. Model interpretation was based on component loadings and variable importance in projection (VIP) scores > 1. Multivariate analysis of variance (MANOVA) was used to assess overall differences between patients with SLE and healthy controls. Model performance was evaluated using repeated k-fold cross-validation, and classification accuracy was assessed using the balanced error rate (BER). Multivariate analyses were conducted using the mixOmics, FactoMineR, and yacca packages.

To assess the discriminatory performance of selected markers, receiver operating characteristic (ROC) curve analysis was performed using variables with VIP scores greater than 1. Classification performance was evaluated by calculating the area under the curve (AUC) with 95% confidence intervals, to determine the ability of these variables to distinguish the remission status. Analyses were performed using the pROC package.

For the same immune cell subsets used in ROC analyses, CB1R/CB2R expression distributions were visualized using boxplots with overlaid individual data points, stratified into healthy controls, active SLE, and remission SLE groups. Statistical significance between groups was assessed using pairwise Wilcoxon rank-sum tests with Benjamini–Hochberg correction for multiple comparisons.

For clinical variables associations, analyses were stratified according to variable type. Numeric clinical variables were analyzed together with laboratory variables using Spearman correlation, with statistical significance determined using FDR-adjusted p-values. Categorical clinical variables with two levels were analyzed using Student’s t-test, and Cohen’s d was calculated to assess the effect size and magnitude of the differences, with associated p-values corrected for FDR. Effect sizes were calculated using the effsize package.

## 3 Results

A total of 35 women with SLE and 35 age-matched healthy female controls were included. The mean age of participants was comparable between groups (SLE 36.5 ± 12.7 vs. controls 36.7 ± 12.6 years). Among patients, the median disease duration was 99 months (IQR 41.5-174), and the median SLEDAI score at sampling was 4 (IQR 0-23.5). Articular, hematologic, and renal involvement were the most frequent clinical manifestations, and 51.4% of patients were classified as being in remission at the time of evaluation. Use of systemic glucocorticoids, antimalarials, and mycophenolate mofetil was common. Baseline demographic and clinical characteristics are summarized in **Table 2**.

**Table 2:** Characteristics of patients with systemic lupus erythematosus and healthy controls.

General characteristics	SLE patients (n=35)	Healthy controls (n=35)
Age, years/mean (SD)	36.5 ± 12.7	36.7 ± 12.6
Sex, female/n (%)	35 (100)	35 (100)
Cannabis use, yes/n (%)	2 (5.7)	5 (14.3)
Diagnosis time, months/median (IQR)	99 (41.5-174)	–
SLEDAI, median (IQR)	4 (0-23.5)	–
Systemic manifestations, yes/n (%)		
Psychiatric	6 (17.1)	–
Articular	24 (68.6)	–
Cutaneous	18 (51.4)	–
Mucosal	3 (8.6)	–
Hematologic	25 (71.4)	–
Renal	19 (54.3)	–
Serosal	8 (22.9)	–
Pulmonary	4 (11.4)	–
Constitutional	5 (14.3)	–
Immunologic	23 (65.7)	–
C3 levels, mean (SD)	91.7 ± 34.2	–
C4 levels, median (IQR)	14 (7.73-17.5)	
Remission, n (%)	18 (51.4)	–
Treatment, n (%)		
Steroids	25 (71.4)	–
Chloroquine/Hydroxychloroquine	15 (42.3)	–
Mycophenolate Mofetil	15 (42.3)	–
Other immunosuppressants	9 (25.7)	–

**Figure 1:**
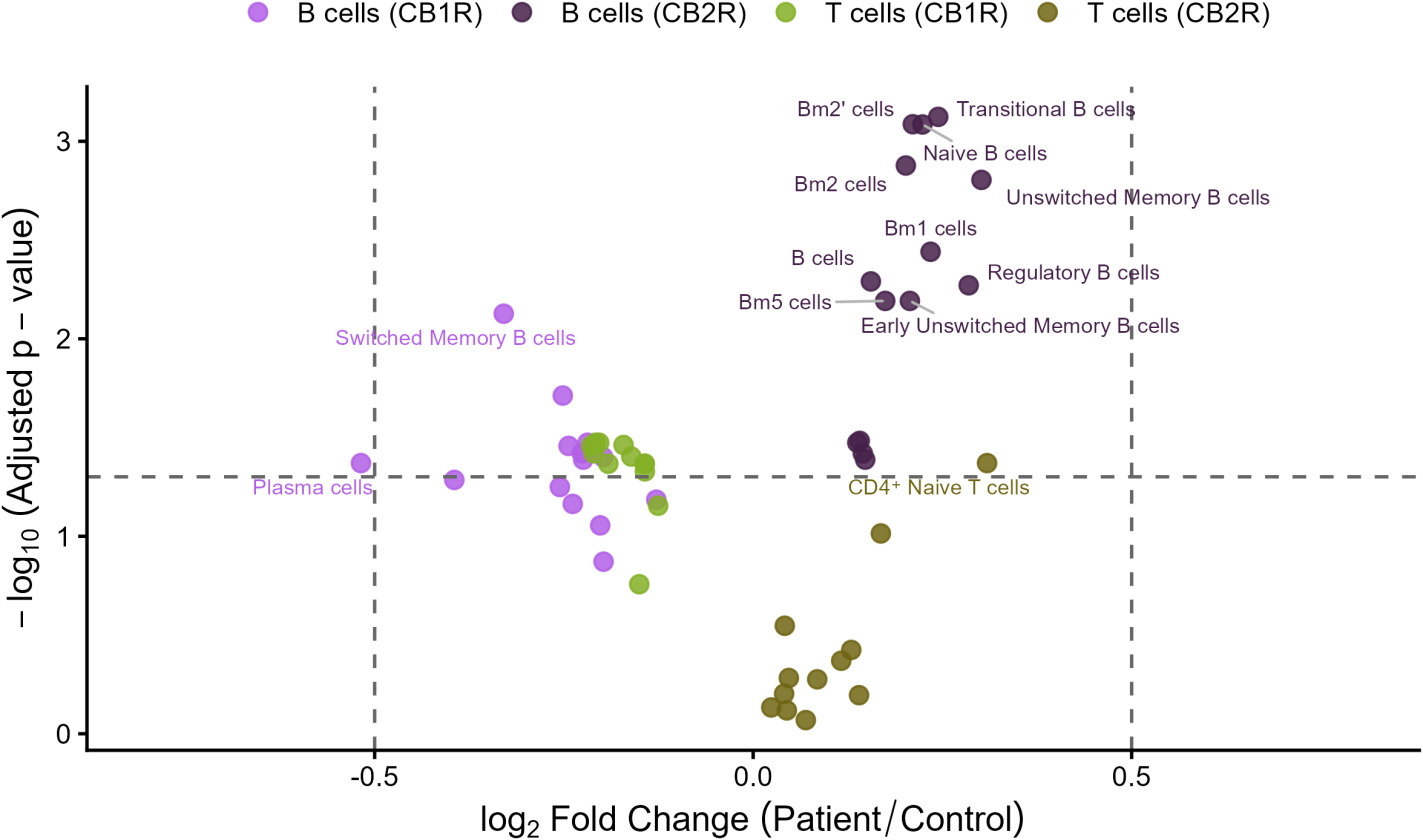
Differential cannabinoid receptor expression across lymphocyte subsets in SLE. Volcano plot showing differential CB1R and CB2R expression across B- and T- lymphocyte subsets in patients with SLE compared with healthy controls. The x-axis represents the log2 fold change in MFI values (Patient/Control), and the y-axis represents -log10 of FDR-adjusted p-values. The horizontal dashed line indicates the statistical significance threshold corresponding to FDR-adjusted p < 0.05, whereas vertical dashed lines indicate reference effect-size thresholds at absolute fold-change values of 0.5 (~1.4-fold change). Labeled subsets correspond to subsets showing the strongest differential expression patterns after FDR correction. significant differences after FDR adjustment.

Group-wise comparisons of cannabinoid receptors expression were performed across B- and T-lymphocyte subsets to evaluate differences between patients with SLE and healthy controls. The results of these analyses are summarized in **Table 3** and visually represented in the volcano plot shown in **Figure 1**. Compared with healthy controls, patients with SLE displayed consistently higher CB2R MFI across a broad range of B-cell subsets. Significant FDR-adjusted differences were observed in total B cells, naïve B cells, transitional and regulatory B cells, early unswitched, unswitched and switched memory B cells, Bm1, Bm2, Bm2’, Bm3-4, eBm5, Bm5 subsets, and plasma cells (FDR < 0.05 for all). In contrast, CB2R MFI in T-cell subsets showed limited differences between groups after FDR correction. Only CD4^+^ naïve T cells exhibited significantly higher CB2R expression in patients with SLE compared with controls, whereas total T cells, CD4**^+^** and CD8**^+^** T cells, and most memory/effector T-cell subsets did not differ significantly.

**Table 3:** Group comparison between SLE and control participants.

MFI variable	CB1R		CB2R	
	*p* value	*p* value (FDR adjusted)	*p* value	*p* value (FDR adjusted)
B cells	0.01298^b^	0.0349*	0.00069^a^	0.0051**
Naive B cells	0.01630^b^	0.0381*	0.00005^a^	0.0008***
Transitional B cells	0.03791^a^	0.0563	0.00001^a^	0.0008***
Regulatory B cells	0.02105^a^	0.0409*	0.00082^a^	0.0054**
Early Unswitched Memory B cells	0.04521^b^	0.0653	0.00115^a^	0.0064**
Unswitched Memory B cells	0.10606^a^	0.1345	0.00015^a^	0.0016**
Switched Memory B cells	0.00158^b^	0.0075**	0.01683^b^	0.0381*
Bm1 cells	0.06607^a^	0.0881	0.00042^a^	0.0036**
Bm2 cells	0.01911^b^	0.0398*	0.00010^a^	0.0013**
Bm2’ cells	0.04879^a^	0.0686	0.00003^a^	0.0008***
Bm3–4 cells	0.03390^b^	0.0518	0.00911^b^	0.0336*
eBm5 cells	0.00447^b^	0.0194*	0.00822^b^	0.0329*
Bm5 cells	0.00993^b^	0.0336*	0.00124^b^	0.0064**
Plasma cells (n=32)	0.02347^b^	0.0426*	0.02125^b^	0.0409*
T cells	0.01195^b^	0.0345*	0.31850^a^	0.3764
CD4^+^ T cells	0.02642^b^	0.0429*	0.23480^a^	0.2839
CD8^+^ T cells	0.01604^b^	0.0381*	0.36946^a^	0.4269
CD4^+^ Naive T cells	0.05118^b^	0.0700	0.02377^a^	0.0426*
CD8^+^ Naive T cells	0.02563^b^	0.0429*	0.07444^a^	0.0968
CD4^+^ Effector T cells	0.02524^b^	0.0429*	0.60117^b^	0.6380
CD8^+^ Effector T cells	0.01099^b^	0.0336*	0.46174^a^	0.5220
CD4^+^ Effector Memory T cells	0.01823^b^	0.0395*	0.57936^a^	0.6276
CD8^+^ Effector Memory T cells	0.01099^b^	0.0336*	0.74686^a^	0.7615
CD4^+^ Central Memory T cells	0.02978^b^	0.0469*	0.85305^a^	0.8530
CD8^+^ Central Memory T cells	0.01341^b^	0.0349*	0.47918^a^	0.5302
CD4^+^ Regulatory T cells (n=25)	0.14133^b^	0.1750	0.70768^a^	0.7360

^a^ t-test; ^b^ Mann–Whitney U test. *p<0.05, **p<0.01, ***p<0.001 (FDR adjusted).

By comparison, CB1R expression also differed significantly between groups across multiple B-cell subsets, including total B cells, naïve, regulatory, switched memory B cells, Bm2, eBm5, Bm5 subsets, and plasma cells (FDR <0.05). Unlike the B cell-focused pattern observed for CB2R, CB1R expression showed broader alterations across the T-cell compartment. Significant differences were detected in total T cells as well as in several CD4**^+^** and CD8**^+^** subsets, particularly within naïve, effector, central memory and effector memory phenotypes.

**Figure 2:**
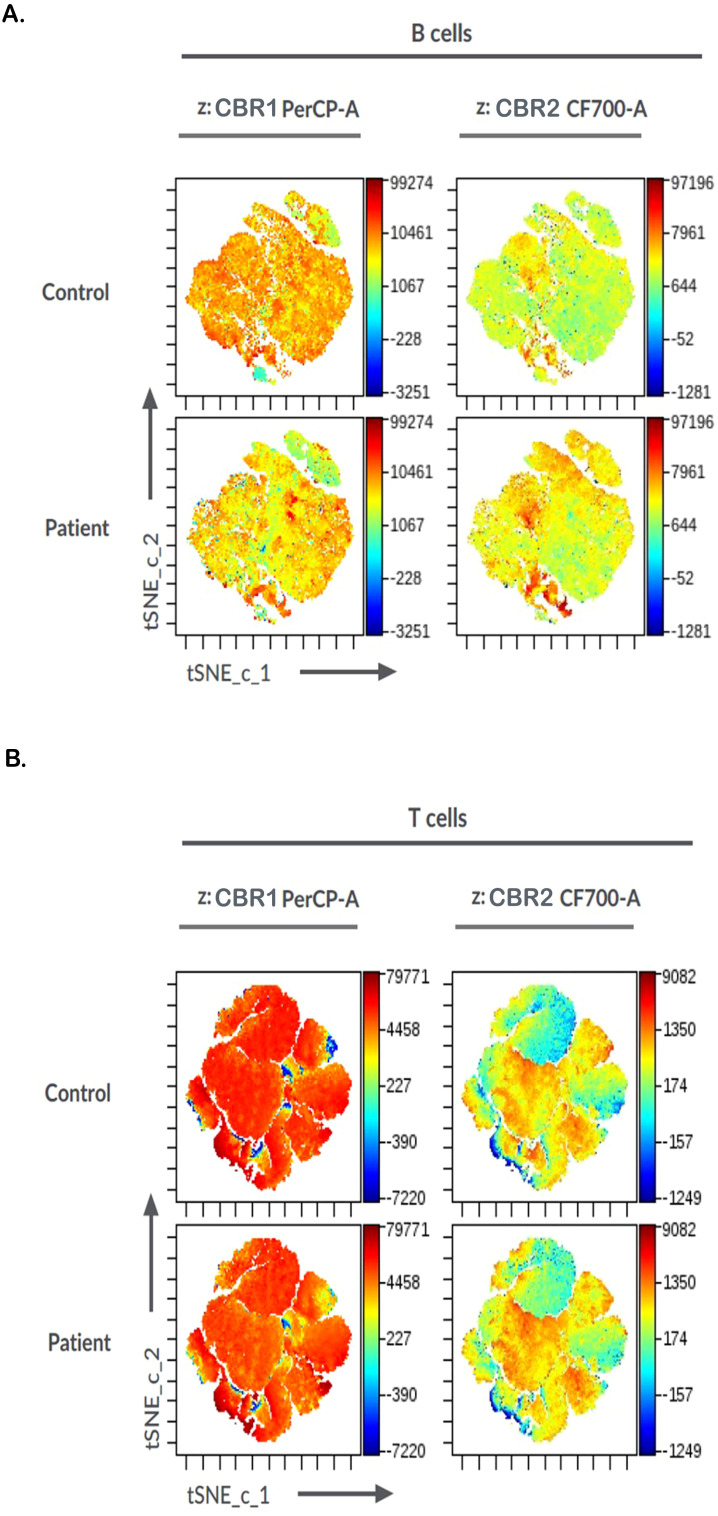
t-SNE visualization of CB1R and CB2R expression in B- and T-cell compartments from controls and patients with SLE. Two-dimensional t-distributed stochastic neighbor embedding (t-SNE) maps illustrating the expression of cannabinoid receptors 1 and 2 in peripheral blood lymphocytes from healthy controls and patients with systemic lupus erythematosus (SLE). **(A)** B cells and **(B)** T cells are shown separately. For each lineage, cells from controls (upper panels) and patients (lower panels) are displayed on the same t-SNE coordinates. Color intensity represents z-score-scaled median fluorescence intensity (MFI) of CB1R (left panels) and CB2R (right panels), as measured by multicolor flow cytometry. Each point corresponds to a single cell, and color gradients reflect relative receptor expression across phenotypically distinct cell clusters. Axes represent t-SNE components 1 and 2. The distribution of individual subsets in the t-SNE analysis is shown in the Supplementary Material (**Supplementary 2**).

**Figure 3:**
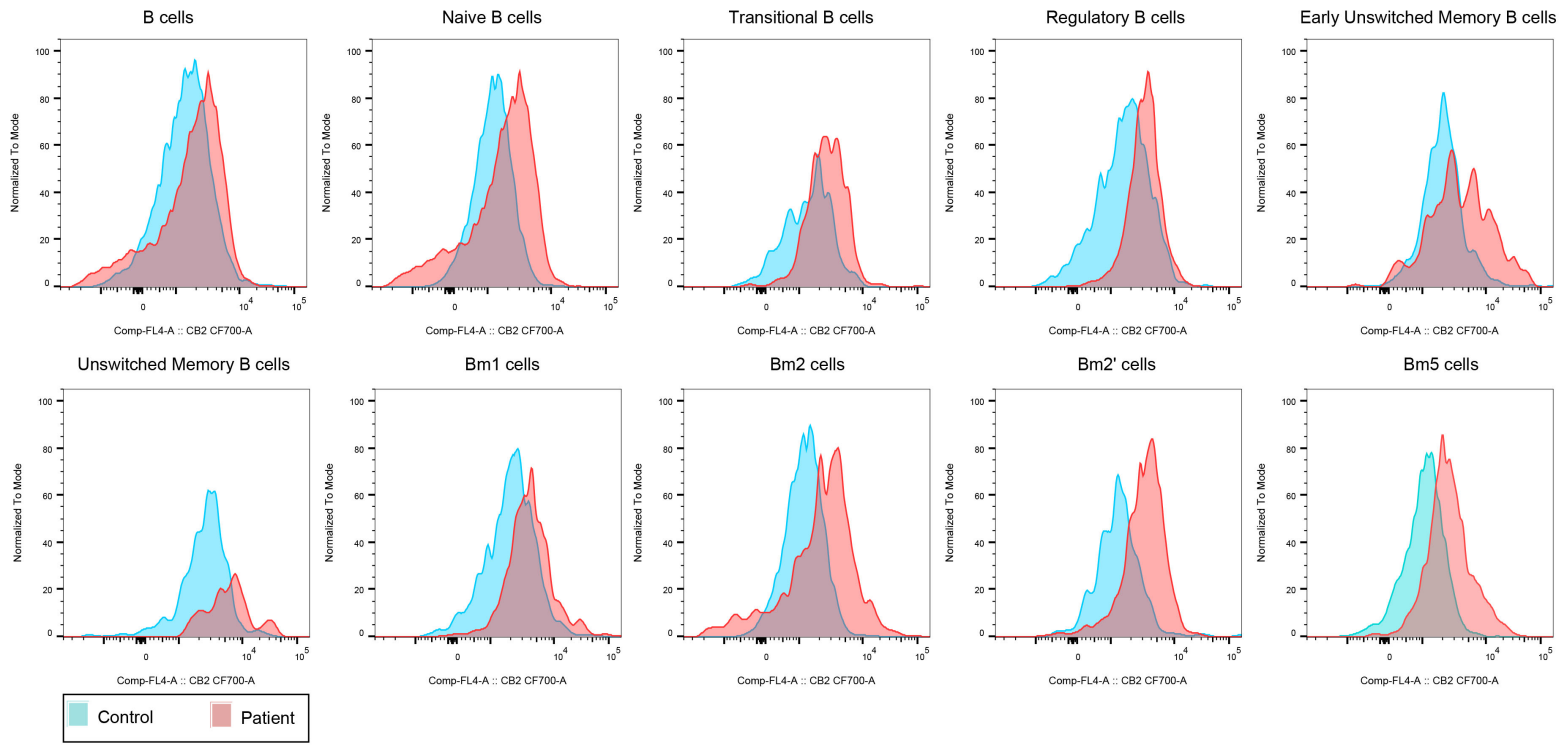
Representative flow cytometry histogram of CB2R expression. Representative histogram overlays showing CB2R expression in selected B-cell subsets from patients with SLE and healthy controls. Histograms correspond to B-cell subsets showing the most significant differences after FDR correction in univariate analyses. Red histograms represent patients with SLE and blue histograms represent healthy controls.

To contextualize these univariate findings within the global immunophenotypic landscape, high-dimensional visualization approaches were applied. The t-SNE representation showed a differential distribution patterns of B- and T-cell subsets based on CB1R and CB2R expressions in patients with SLE compared with healthy controls. Patterns observed in the t-SNE maps were consistent with differences identified in the univariate analysis. Notably, CB1R expression showed a relative reduction across multiple T and B cell subsets in patients with SLE, whereas CB2R expression was increased primarily within B-cell subsets and CD4**^+^** Naive T cells in patients (**Figure 2**). Representative flow cytometry histogram overlays illustrating CB2R expression patterns in the most significantly altered B-cells subsets are shown in **Figure 3**, and complete subset distribution is detailed in the supplementary material (**Supplementary Figure 2**).

**Figure 4:**
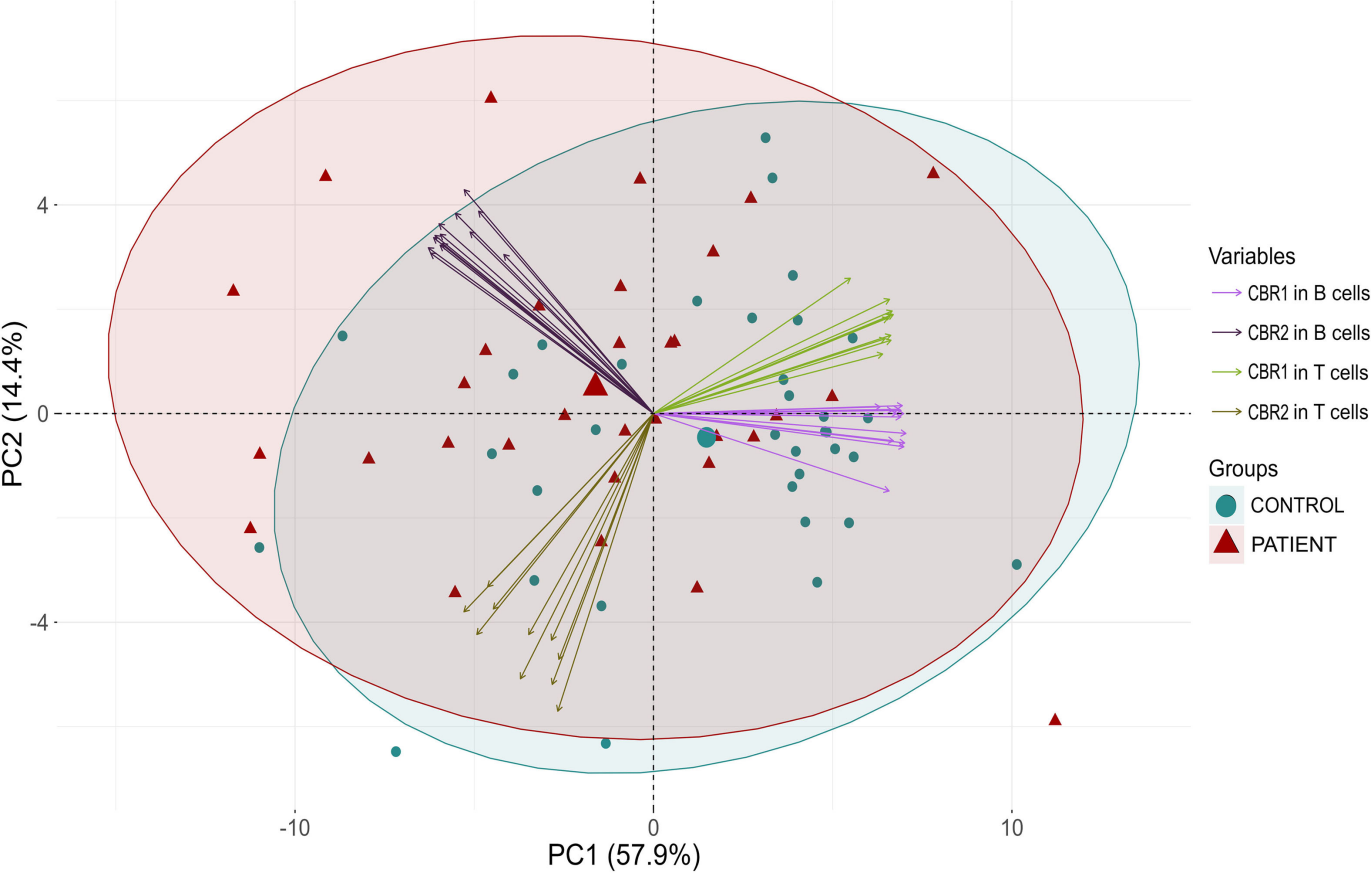
Principal component analysis reveals coordinated patterns of CB1R and CB2R expression across lymphocyte lineages in SLE. Principal component analysis (PCA) biplot summarizing the multivariate structure of CB1R and CB2R expression across B- and T-cell subsets in healthy controls and patients with systemic lupus erythematosus (SLE). Individual points represent study participants, colored by group (controls vs patients), with ellipses indicating the 95% confidence region for each group. The first two principal components accounted for a substantial proportion of the total variance (PC1: 57.9%; PC2: 14.4%). Vectors represent the contribution and directionality of cannabinoid receptors expression across B-cell and T-cell subsets to the principal components. The length of each vector reflects its relative contribution to variance, whereas the orientation indicates coordinated expression patterns among related immune variables.

Principal component analysis (PCA) demonstrated that the first two principal components explained 57.9% and 14.6% of the total variance, respectively (**Figure 4**). Although healthy controls and patients with SLE did not form completely discrete clusters, a partial separation along PC1 was observed, indicating global differences in CB1R and CB2R expression profiles across lymphocyte subsets between groups. Inspection of the loading vectors revealed that CB1R expression in B-cell subsets contributed predominantly to PC1, whereas CB2R expression in B cells loaded mainly along PC2, reflecting distinct axes of variability within the B-cell compartment. In contrast, CB1R and CB2R expressions in T-cell subsets contributed less to the overall variance, as indicated by shorter loading vectors.

**Figure 5:**
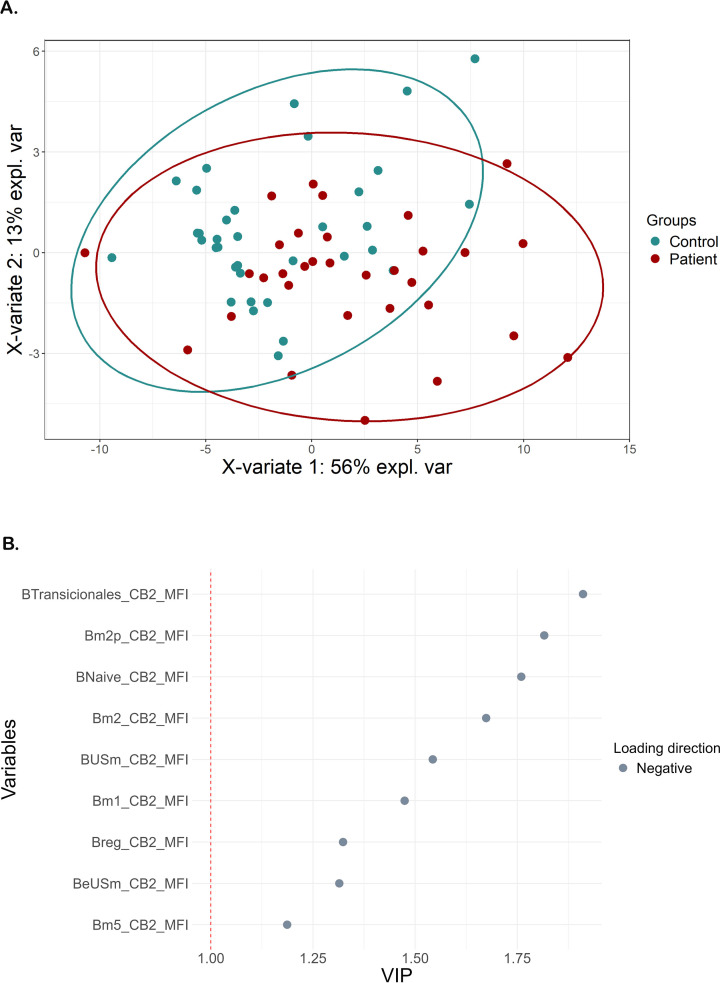
Partial least squares discriminant analysis of multivariate cannabinoid receptor expression profiles in SLE. Partial least squares discriminant analysis (PLS-DA) performed using multivariate cannabinoid receptor expression variables across lymphocyte subsets from healthy controls and patients with systemic lupus erythematosus (SLE). **(A)** PLS-DA score plot showing individual participants projected onto the first two latent components (X-variates). Each point represents one subject and is colored according to study group (control or patient). Ellipses indicate the 95% confidence region for each group, and the percentage of explained variance is shown for each component. **(B)** Variable importance in projection (VIP) scores for component 1 of the PLS-DA model. Each point represents a laboratory variable included in the model, with higher VIP values indicating greater contribution to the model projection. The dashed vertical line indicates the VIP threshold of 1.0. Variables are labeled according to lymphocyte subset and corresponding cannabinoid receptor median fluorescence intensity (MFI).

Supervised multivariate analysis using PLS-DA showed a greater degree of separation between patients with SLE and healthy controls based on CB1R and CB2R expression across lymphocyte subsets compared with the unsupervised PCA, while preserving interindividual heterogeneity (**Figure 5A**). This separation was primarily associated with the first latent variable. Analysis of VIP scores identified CB2R expression in B-cell subsets as the principal contributors to group discrimination (VIP> 1). The highest-ranking variables included CB2R expression in transitional, naïve, regulatory, and early memory B-cell compartments, whereas CB1R-related variables and T-cell subsets showed lower discriminative relevance (**Figure 5B**). MANOVA indicated a significant overall difference between patients with SLE and healthy controls across CB2R-related B-cell variables with VIP scores greater than 1 (MANOVA, p= 0.00033). *Post hoc* analyses demonstrated that each of these subsets remained significantly different between groups, consistently showing higher CB2R expression in patients with SLE compared with controls (**Supplementary Table 1**).

Exploratory correlation analyses with clinical variables revealed significant associations between laboratory immune variables and five clinical features, including disease activity (SLEDAI score), remission status, hemoglobin levels, and articular and mucosal involvement. Correlation analyses were performed across the complete set of laboratory and clinical variables, with comprehensive results provided in the Supplementary Material (**Supplementary Table 2**). Here, only associations reaching statistical significance with at least one clinical parameter are presented. In total, 36 laboratory variables showed significant associations with at least one clinical feature, with effect sizes predominantly in the moderate to large range (**Table 4**).

**Table 4:** Associations between cannabinoid receptor expression and clinical variables.

Variables		*p*-value	*p*-value (FDR adjusted)	Effect size
Clinics	Laboratory			
Articular involvement	CB2R^+^CD4^+^ Effector Memory T cells	0, 0009	0, 0169	1, 14^a^
	CB2R^+^CD4^+^ Central Memory T cells	0, 0034	0, 0489	0, 98^a^
	CB2R^+^ T regulatory cells	0, 0014	0, 0155	1, 31^a^
Mucosal involvement	CB2R^+^ T cells	0, 0010	0, 0170	-0, 85^a^
	CB2R^+^CD4^+^ T cells	0, 0005	0, 0115	-0, 80^a^
	CB2R^+^CD8^+^ T cells	0, 0004	0, 0111	-0, 87^a^
	CB2R^+^CD8^+^ Naive T cells	0, 0005	0, 0112	-0, 98^a^
Hemoglobin	CB1R^+^ Bm2 cells	0, 0028	0, 0487	0, 51^b^
	CB1R^+^ Unswitched Memory B cells	0, 0028	0, 0487	0, 51^b^
SLEDAI	CB1R^+^ B cells	0, 0000	0, 0022	-0, 67^b^
		0, 0000	0, 0053	-1, 84^a^
	CB1R^+^ Early Unswitched Memory B cells	0, 0009	0, 0213	-0, 56^b^
		0, 0005	0, 0111	-1, 39^a^
	CB1R^+^ Bm1 cells	0, 0000	0, 0021	-0, 69^b^
		0, 0000	0, 0056	-1, 72^a^
	CB1R^+^ Bm2 cells	0, 0001	0, 0046	-0, 63^b^
		0, 0000	0, 0056	-1, 74^a^
	CB2R^+^ Bm2 cells	nsc	nsc	–
		0, 0029	0, 0450	1, 17^a^
	CB1R^+^ Bm2’ cells	0, 0001	0, 0046	-0, 64^b^
		0, 0001	0, 0074	-1, 60^a^
	CB2R^+^ Bm2’ cells	0, 0016	0, 0305	0, 54^b^
		0, 0034	0, 0489	1, 13^a^
	CB1R^+^ Bm3–4 cells	0, 0005	0, 0145	-0, 58^b^
		0, 0002	0, 0084	-1, 53^a^
	CB1R^+^ Bm5 cells	0, 0000	0, 0021	-0, 68^b^
		0, 0001	0, 0074	-1, 69^a^
	CB1R^+^ Naive B cells	0, 0000	0, 0021	-0, 69^b^
		0, 0000	0, 0053	-1, 83^a^
	CB2R^+^ Naive B cells	nsc	nsc	–
		0, 0033	0, 0489	1, 14^a^
	CB1R^+^ Plasma cells	nsc	nsc	–
		0, 0034	0, 0489	-1, 15^a^
	CB1R^+^ Regulatory B cells	0, 0000	0, 0021	-0, 68^b^
		0, 0000	0, 0053	-1, 80^a^
	CB1R^+^ Switched Memory B cells	0, 0005	0, 0136	-0, 58^b^
		0, 0003	0, 0101	-1, 45^a^
Remission status	CB1R^+^ Transitional B cells	0, 0000	0, 0028	-0, 66^b^
		0, 0000	0, 0056	-1, 69^a^
	CB1R^+^ Unswitched Memory B cells	0, 0001	0, 0044	-0, 64^b^
		0, 0002	0, 0084	-1, 52^a^
	CB1R^+^ eBm5 B cells	0, 0000	0, 0028	-0, 66^b^
		0, 0000	0, 0056	-1, 74^a^
	CB1R^+^ T cells	0, 0005	0, 0136	-0, 58^b^
		0, 0001	0, 0083	-1, 58^a^
	CB1R^+^CD4^+^ T cells	0, 0007	0, 0185	-0, 57^b^
		0, 0001	0, 0075	-1, 63^a^
	CB1R^+^CD4^+^ Effector T cells	nsc	nsc	–
		0, 0003	0, 0101	-1, 46^a^
	CB2R^+^ CD4^+^ Effector T cells	0, 0010	0, 0217	0, 55^b^
		0, 0010	0, 0170	1, 30^a^
	CB1R^+^CD4^+^ Effector Memory T cells	0, 0004	0, 0130	-0, 59^b^
		0, 0001	0, 0079	-1, 60^a^
	CB1R^+^CD4^+^ Central Memory T cells	0, 0002	0, 0092	-0, 61^b^
		0, 0001	0, 0074	-1, 67^a^
	CB1R^+^CD4^+^ Naive T cells	0, 0016	0, 0305	-0, 53^b^
		0, 0002	0, 0084	-1, 55^a^
	CB1R^+^CD8^+^ T cells	0, 0009	0, 0209	-0, 56^b^
		0, 0003	0, 0101	-1, 46^a^
	CB1R^+^CD8^+^ Effector T cells	0, 0029	0, 0491	-0, 51^b^
		0, 0005	0, 0112	-1, 39^a^
	CB1R^+^CD8^+^ Effector Memory T cells	0, 0005	0, 0136	-0, 58^b^
		0, 0002	0, 0096	-1, 48^a^
	CB1R^+^CD8^+^ Central Memory T cells	0, 0003	0, 0126	-0, 59^b^
		0, 0002	0, 0084	-1, 53^a^
	CB1R^+^ T Regulatory cells (n=25)	nsc	nsc	–
		0, 0001	0, 0025	-1, 66^a^
	CB1R^+^CD8^+^ Naive T cells	0, 0016	0, 0305	-0, 53^b^
		0, 0002	0, 0092	-1, 49^a^

^a^ Cohen’s d; ^b^ ρ;. nsc: not significantly correlated.

Remission status was associated with multiple immune subsets characterized by altered CB1R and CB2R expressions. Most immune features associated with SLEDAI score were also associated with remission status, while additional variables were uniquely linked to clinical remission, both exhibited the broadest pattern of association. Hemoglobin levels were significantly associated with CB1R-expressing B cell subsets, with effect sizes in the moderate range. Among categorical clinical variables, articular involvement was associated with significantly higher CB2R expression in regulatory T cells, CD4^+^ effector memory and central memory T cells, with large effect sizes, whereas mucosal involvement was associated with significantly lower CB2R expression across multiple T cell populations, including total T cells, CD4^+^ T cells, CD8^+^ T cells, and naïve CD8^+^ T subsets.

**Figure 6:**
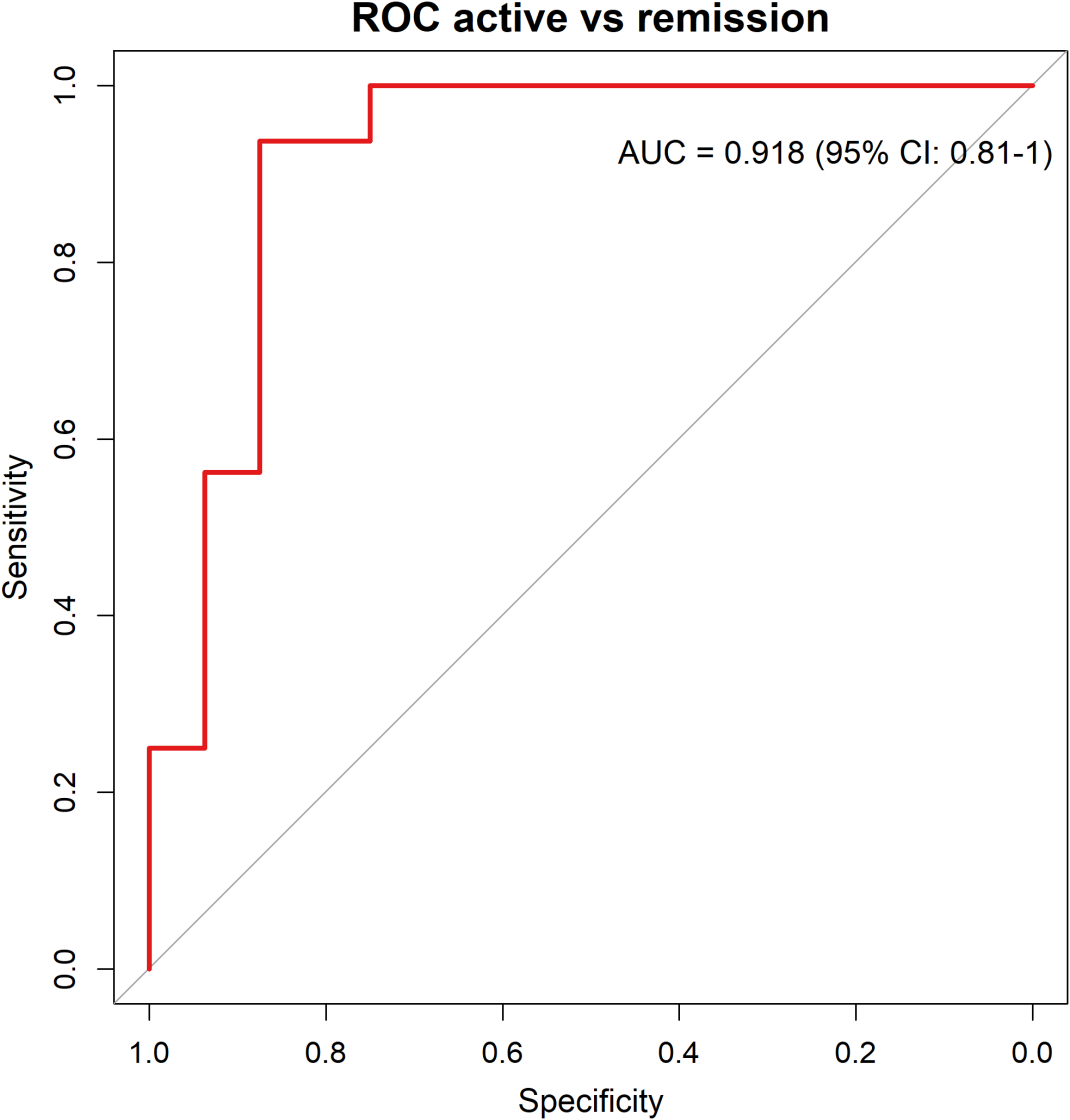
Receiver operating characteristic (ROC) curve for remission status based on multivariate immune variables. Receiver operating characteristic (ROC) curve assessing the performance of a multivariate immune signature in discriminating remission status in patients with systemic lupus erythematosus (SLE). The ROC curve was constructed using the variables identified as relevant by the partial least squares discriminant analysis (PLS-DA) model. The area under the curve (AUC) with 95% confidence interval is indicated in the figure.

**Figure 7:**
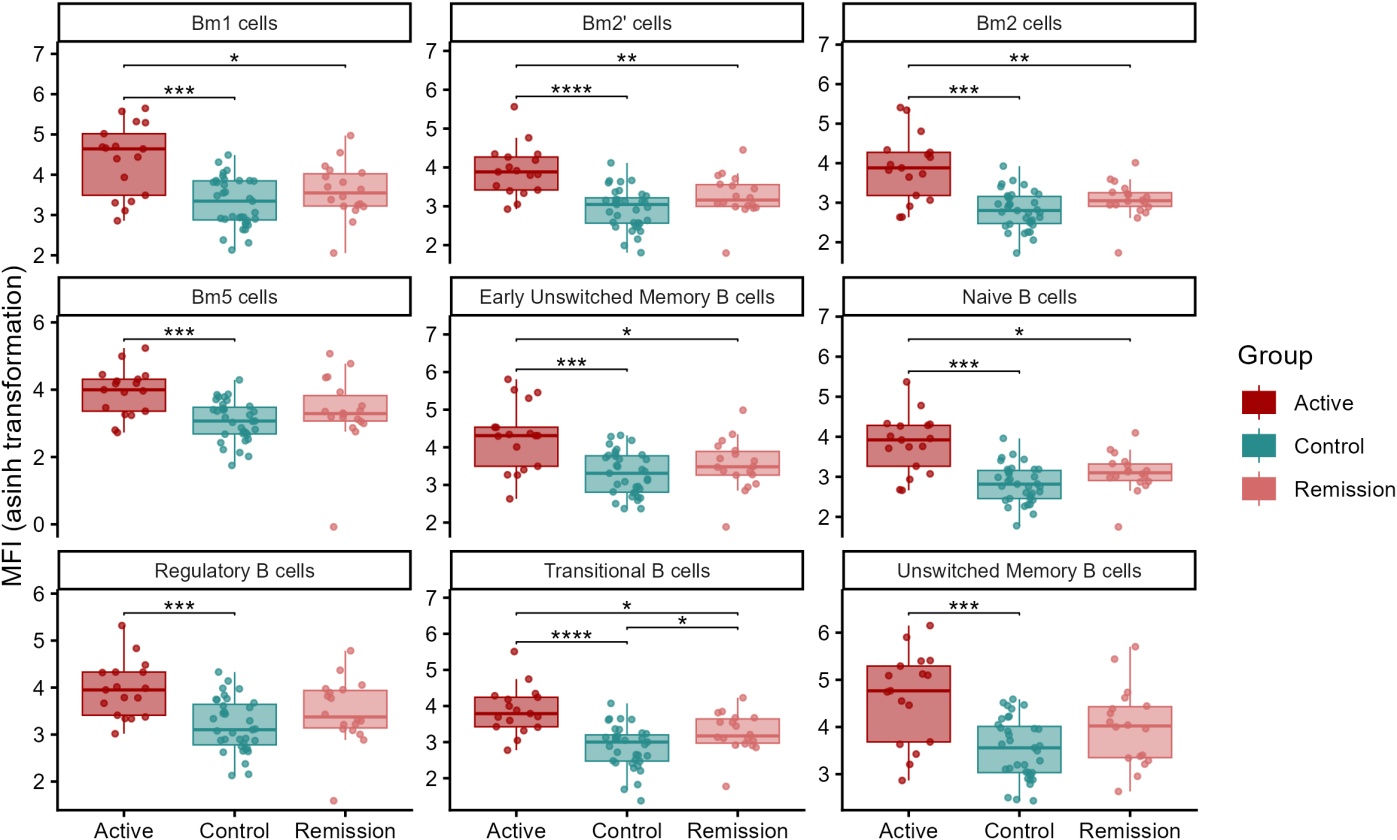
CB2R expression patterns in B-cell subsets associated with remission status. Distribution of BC2R expression levels in selected B-cells subsets from patients with active SLE, patients in remission, and healthy controls. Subsets shown correspond to the principal B-cell subsets identified as relevant contributors to remission status in multivariate and ROC analysis. Data are presented as boxplots with individual observations overlaid. Pairwise group comparisons were performed using Wilcoxon rank-sum tests with FDR correction for multiple comparisons. Statistical significance is indicated as follows: *p < 0.05, **p < 0.01, ***p < 0.001, ****p < 0.0001..

Finally, the discriminative capacity of the immune variables identified by the PLS-DA model was evaluated in relation to remission status using ROC analysis. The resulting model showed an area under the curve (AUC) of 0.918 (95% CI: 0.81–1.00) within the study cohort (**Figure 6**), suggesting a potential capacity of the selected immune features to differentiate remission from active disease in this dataset. To further visualize the distribution of the principal B-cell subsets contributing to remission status discrimination, CB2R expression levels across active SLE, remission SLE, and healthy controls are shown in **Figure 7**.

## 4 Discussion

The endocannabinoid system has emerged as an important modulator of immune responses, contributing to the regulation of inflammation, immune activation, and immune tolerance (17). Among its components, CB1R and CB2R play distinct roles within the immune system. CB2R is preferentially expressed in immune cells and has been associated with immunoregulatory and predominantly anti-inflammatory functions, whereas CB1R, although primarily described in the nervous system, is also detectable in peripheral immune cells in a context-dependent manner (19). Despite increasing interest in the immunological functions of cannabinoid receptors, their characterization in human autoimmune diseases remains limited (17, 24–26). SLE is an autoimmune disease characterized by dysregulated immune responses involving altered B-cell activation, impaired immune tolerance, and changes in T-cell function. Evidence on cannabinoid receptor involvement has been limited (3). Previous studies have reported the presence of CNR1 and CNR2 mRNA in peripheral blood mononuclear cells from patients with SLE, as well as correlations between CNR2 transcript levels and complement components C3 and C4; however, these approaches do not provide information on receptor expression at the protein level or within defined immune cell subsets (27).

In this study, we addressed this gap by providing a comprehensive immunophenotypic characterization of CB1R and CB2R expression across major B- and T-lymphocyte subsets in patients with SLE. By integrating univariate analyses with high-dimensional and multivariate approaches, we show that alterations in cannabinoid receptor expression in SLE are not uniformly distributed across the immune system. Instead, our results consistently identify the B-cell compartment as the major contributor to group differentiation between patients and healthy controls, with CB2R expression emerging as a dominant feature across multiple stages of B-cell differentiation. In contrast, changes in CB1R expression were more heterogeneous and extended to both B- and T-cell subsets but contributed less strongly to group discrimination in multivariate models. Notably, however, CB1R-associated variables showed a higher degree of association with clinical features, suggesting a potential link between CB1R expression patterns and clinical heterogeneity within SLE, whereas CB2R expression primarily reflects broader immunophenotypic differences between groups.

The preferential involvement of the B-cell compartment is particularly relevant in the context of SLE, where B cells contribute to disease pathogenesis not only through autoantibody production but also by acting as antigen-presenting cells and by shaping T-cell responses via cytokine secretion (28). The observation that CB2R expression was increased across naïve, transitional, regulatory, memory, and plasma cell subsets suggests that cannabinoid signaling pathways may be engaged throughout multiple stages of B-cell differentiation in SLE.

In other autoimmune and inflammatory conditions, increased CB2R expression in immune cells has frequently been interpreted as a response to sustained immune activation, consistent with experimental evidence showing anti-inflammatory and immunomodulatory effects following CB2R engagement (29–33). Within this framework, elevated CB2R expression in B cells has been proposed to reflect an attempt to restrain excessive immune activation and to promote immune homeostasis in chronically inflamed environments. These findings are consistent with ***in vitro*** studies demonstrating anti-inflammatory effects mediated by CB2R activation, including inhibition of T-cell recruitment, reduced Th1/Th2 polarization, decreased T-cell proliferation, and increased T-cell apoptosis after CB2R agonist stimulation (34–40). CB2R activation has also been shown to modulate B-cell migration, promote B-cell expansion, enhance retention of immature B cells in bone marrow, and increase CB2R expression within germinal centers (41–45). Although this evidence is not directly related to the altered receptor expression observed in the present study, it further suggests the functional relevance of CB2R in immune regulation. In this context, our findings raise the possibility that altered CB2R expression in SLE may be associated with immunoregulatory processes across B-cell subsets; however, given the disease-specific immune dysregulation that characterizes SLE, functional studies in this context are required to determine whether this expression patterns translate into mechanistic or clinically relevant effects.

In immune-mediated diseases characterized by impaired tolerance, sustained overexpression of receptors involved in survival, activation, or differentiation pathways may paradoxically contribute to disease persistence by altering activation thresholds or promoting the survival of autoreactive cells. Under such conditions, signaling pathways that are homeostatic under physiological circumstances may become maladaptive when chronically engaged. This context-dependent interpretation of CB2R signaling is illustrated by observations from other disease settings. In melanoma, higher CB2R expression has been associated with improved survival and increased intratumoral B-cell infiltration, while experimental models have shown that CB2R deficiency impairs B-cell development and differentiation (42, 46). These findings are consistent with a role for CB2R in B-cell survival and functional competence under physiological or antitumor immune conditions.

In contrast, in autoimmune diseases such as SLE, where peripheral B-cell tolerance is already compromised, similar signaling pathways may have different consequences (1). Chronic engagement of receptors involved in B-cell survival and activation can favor the persistence of autoreactive clones, as exemplified by BAFF overexpression in SLE, which enables naïve and transitional autoreactive B cells to escape anergy and negative selection (1, 28, 47). In this setting, the widespread increase of CB2R expression observed across early and mature B-cell subsets may be compatible with a dysregulated engagement of pathways that are otherwise homeostatic, potentially contributing to immune persistence rather than resolution. However, this interpretation remains hypothetical and requires functional validation in the context of SLE.

Following the identification of clear group-level differences between patients with SLE and healthy controls, we next examined the relationship between cannabinoid receptor expression and clinical features within the SLE cohort. In these analyses, multiple immune variables were associated with disease-related clinical parameters, including disease activity, remission status, hemoglobin levels, and specific organ involvement. Among clinical measures, SLEDAI and remission status were those most consistently associated with laboratory features. Higher SLEDAI scores were specifically associated with a polarized receptor expression pattern characterized by reduced CB1R expression and increased CB2R expression across multiple B-cell subsets, most prominently within transitional and memory compartments. Remission status showed substantial overlap with these associations, while additionally encompassing a subset of immune variables unique to the remission profile.

The strength of these findings is supported by multivariate analyses, including VIP scores and ROC curve evaluation. CB2R expression in Bm2, Bm2’, and naive B cells emerged as key contributors to group differentiation, not only between patients and controls but also between active and remission states. These subsets showed high AUC values within this dataset, suggesting their potential relevance in distinguishing disease activity. Although these findings point to candidate immunophenotypic profiles, they should be interpreted as exploratory and require validation in independent cohorts.

These clinical correlations can be interpreted in the context of previous observations on endocannabinoid signaling dynamics in SLE. Alterations in ligand availability and receptor expression appear to coexist with disease activity, rather than remaining static features of the disease. Elevated circulating levels of 2-arachidonoylglycerol (2-AG), a full agonist of both CB1R and CB2R, have been reported in patients with SLE, with higher concentrations observed in individuals with lower disease activity (27). This pattern suggests a potential relationship between endocannabinoid signaling and disease activity; however, the functional implications of this association remain to be fully elucidated.

CB1R expression has been less studied in SLE; however, studies in other immune-mediated conditions provide contextual information regarding its potential role. In type 1 diabetes, increased CB1R expression has been reported in CD4^+^ T cells and pancreatic β cells, and CB1R blockade has been shown to reduce Th1 polarization (48). Notably, in murine models of cutaneous SLE, CB1R agonists have demonstrated potent anti-inflammatory effects, reducing clinical manifestations, histological damage, and systemic cytokine levels (49). *In vitro* studies further suggest a role for CB1R in immune regulation, as its activation has been associated with a reduced Th1 response, decreased T-cell migration and proliferation, increased apoptosis, and downregulation of activation markers (40). In this framework, the reduced CB1R expression observed in patients with higher disease activity in our study may be associated with a more permissive environment for immune activation. At the same time, upregulation of CB2R in B-cell subsets may reflect a concurrent response to sustained immune activation, consistent with its proposed role in modulating immune responses in inflammatory settings. These observations support a context-dependent immunomodulatory effects of endocannabinoid receptor expression in SLE, although the functional implications of these observations in human disease are not yet fully understood.

In addition, cannabinoid receptor expression was also associated with specific clinical domains. Hemoglobin levels correlated predominantly with CB1R-expressing B-cell subsets, suggesting a possible association between CB1R expression and systemic inflammatory features in SLE, although the underlying mechanisms remain unclear.

Organ-specific associations also suggest that cannabinoid receptor expression patterns may vary across different clinical manifestations of SLE. Articular involvement was associated with higher CB2R expression in CD4^+^ effector and central memory T cells, whereas mucosal involvement correlated with reduced CB2R expression across multiple T-cell populations, suggesting that distinct inflammatory milieus may be associated with differential engagement of cannabinoid receptor pathways. Finally, the association of regulatory T cells with cannabinoid receptor expression highlights the possibility that ECS dysregulation in SLE may extend beyond effector compartments and may influence immunoregulatory balance.

These results should be interpreted in an exploratory context, as the relatively small sample size and cross-sectional design limit causal inference and generalizability. Although multiple comparisons across immune cell subsets were controlled using FDR correction, the possibility of residual false-positive findings cannot be excluded. Therefore, these findings should be considered hypothesis-generating and require validation in independent and longitudinal cohorts.

Finally, the observed associations between cannabinoid receptors expression and clinical parameters support the relevance of the endocannabinoid system as a potentially important immunoregulatory pathway in SLE. Our findings expand the current understanding of cannabinoid receptor expression across immune cell subsets in human disease and highlight the B-cell compartment as a major site of altered receptor expression. In addition, the associations identified with disease activity and clinical manifestations suggest that cannabinoid receptor expression patterns may have potential relevance as candidate immunological markers and warrant further investigation in functional and longitudinal studies.

## Data Availability

The raw data supporting the conclusions of this article will be made available by the authors, without undue reservation.
