# Supplementary material for: Cannabinoid receptor expression profiling in lymphocyte subsets reveals clinically relevant immune patterns in SLE
Source: Front Immunol. 2026 Jun 3;17:1848747. doi: 10.3389/fimmu.2026.1848747 (PMC13272394; doi:10.3389/fimmu.2026.1848747)
Supplement: Supplementary file 1 [file Supplementaryfile1.docx]

Supplementary Material

# Supplementary Figures and Tables

## Supplementary Figures

**Supplementary Figure 1.** Flow cytometry Gatting strategy

1. B cell gating strategy


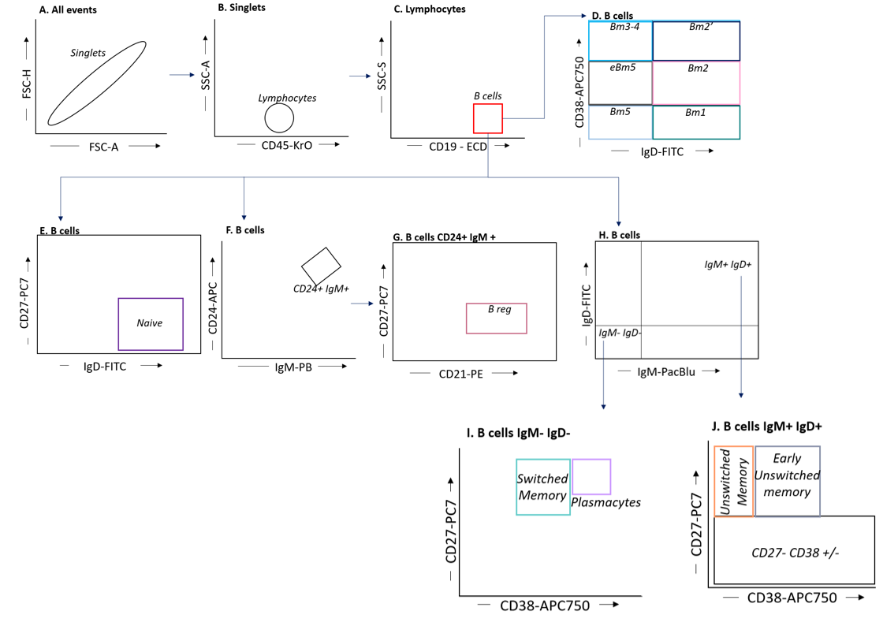


1. T cell gating strategy


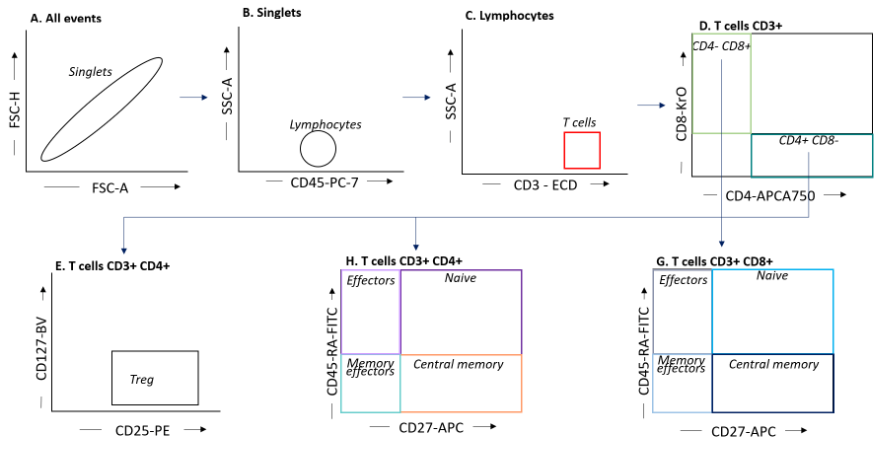


**Supplementary Figure 2.** Distribution of individual subsets in the t-SNE analysis

1. **B cell subsets**

**
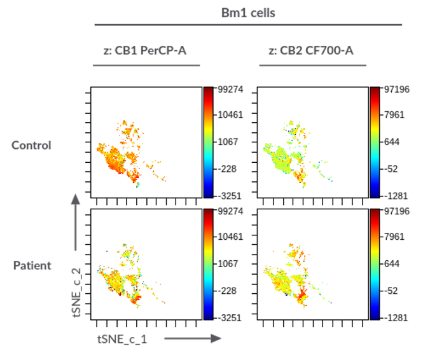
** **
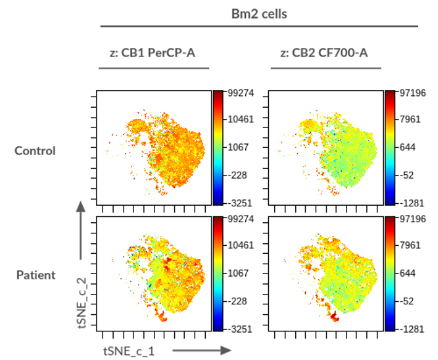

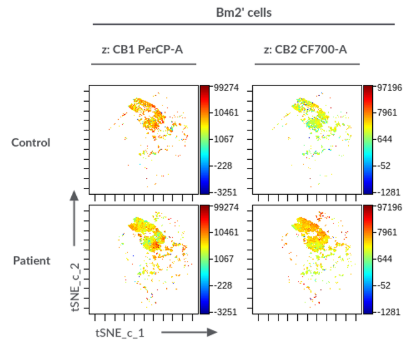

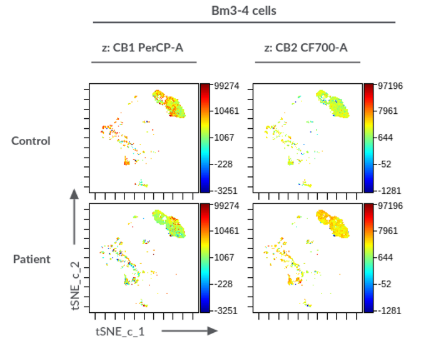
**


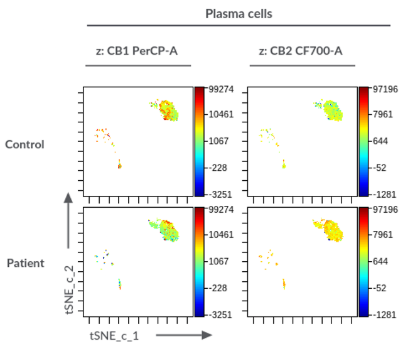
  
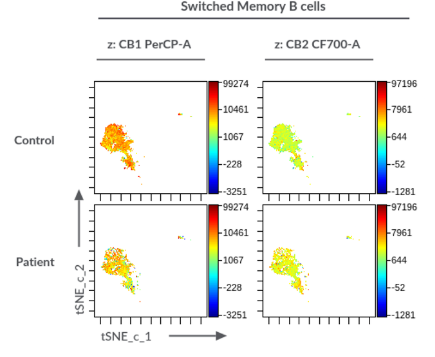
 
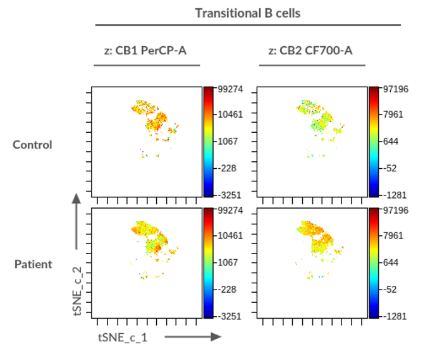
 
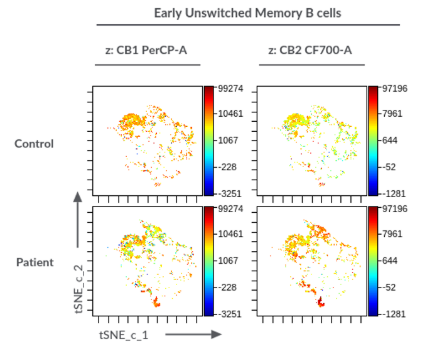
 **
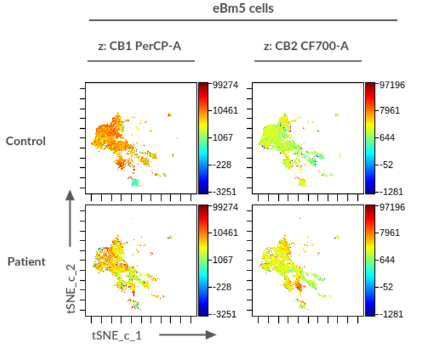

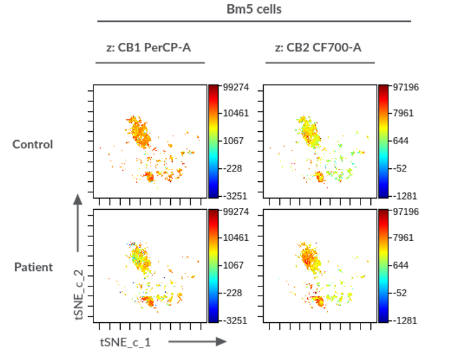
**


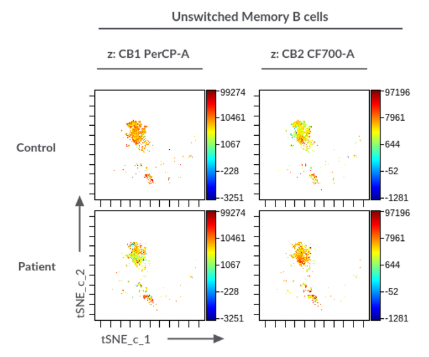
 
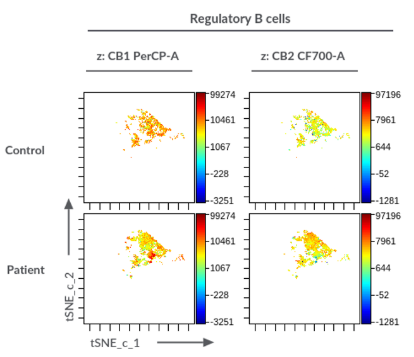
 
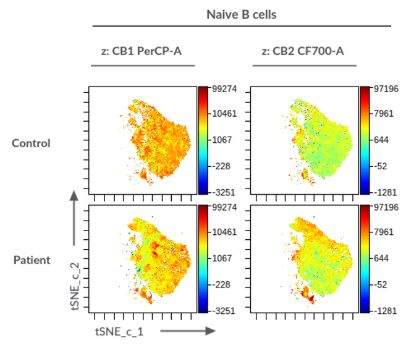


1. **T cell subsets**


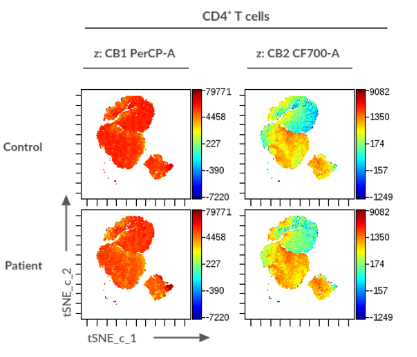

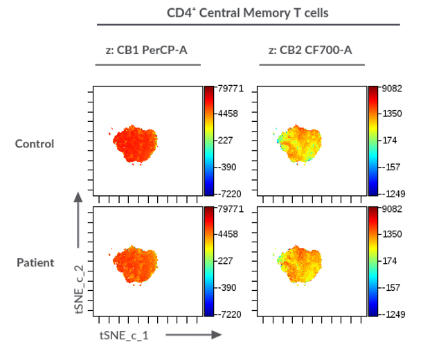


  
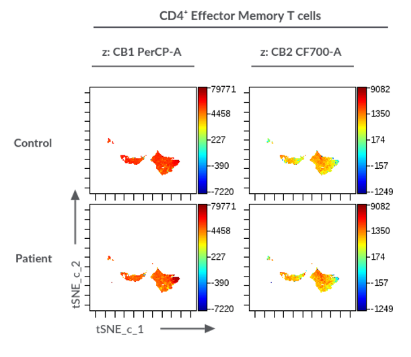
 
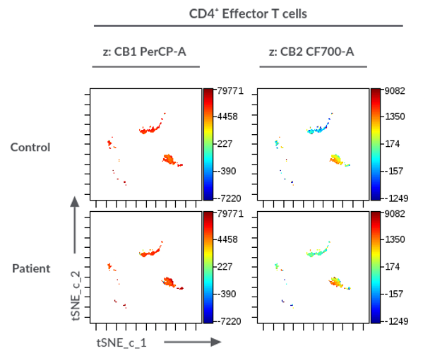
 
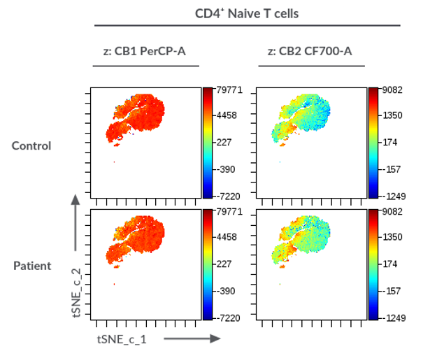
 
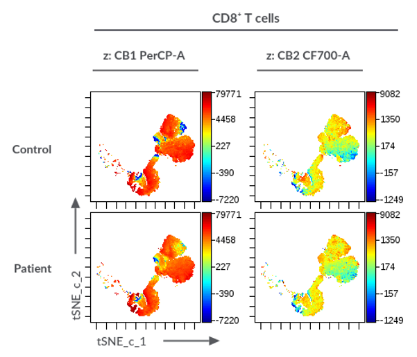

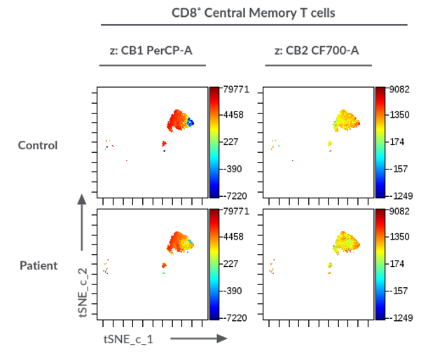

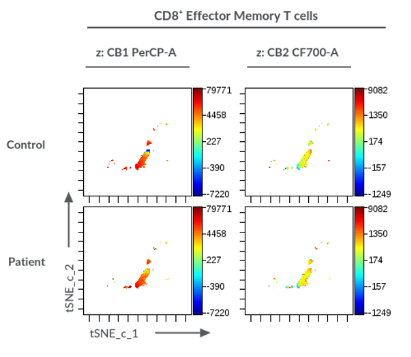


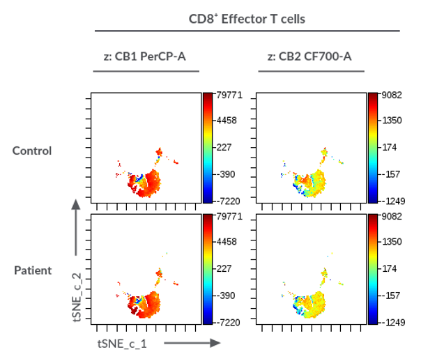
 
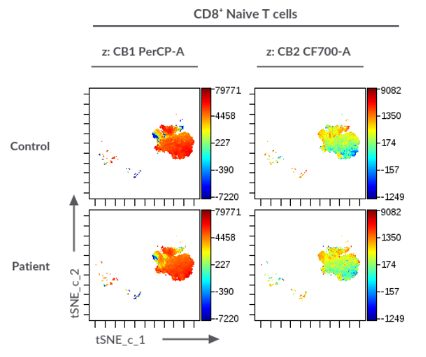


## Supplementary Tables

**Supplementary Table 1.** Manova Post hoc univariate analysis of VIP variables identified by PLS-DA

| **Variable (subset, marker)** | **Group** | **Mean** | **F value** | **Adjusted p-value (FDR)** |
| --- | --- | --- | --- | --- |
| B transitional CB2 MFI | Control | 2.86 | 21.06 | 2.09 × 10⁻⁵ |
|  | SLE | 3.58 |  |  |
| Bm2’ CB2 MFI | Control | 2.93 | 19.26 | 4.29 × 10⁻⁵ |
|  | SLE | 3.59 |  |  |
| Naïve B cells CB2 MFI | Control | 2.82 | 18.43 | 5.99 × 10⁻⁵ |
|  | SLE | 3.46 |  |  |
| Bm2 CB2 MFI | Control | 2.81 | 16.84 | 1.16 × 10⁻⁴ |
|  | SLE | 3.47 |  |  |
| Unswitched memory B CB2 MFI | Control | 3.53 | 14.18 | 3.60 × 10⁻⁴ |
|  | SLE | 4.28 |  |  |
| Bm1 CB2 MFI | Control | 3.31 | 13.41 | 5.05 × 10⁻⁴ |
|  | SLE | 3.98 |  |  |
| Regulatory B cells CB2 MFI | Control | 3.19 | 10.93 | 1.54 × 10⁻³ |
|  | SLE | 3.71 |  |  |
| Early unswitched memory B CB2 MFI | Control | 3.30 | 10.73 | 1.70 × 10⁻³ |
|  | SLE | 3.89 |  |  |
| Bm5 CB2 MFI | Control | 3.07 | 8.48 | 4.91 × 10⁻³ |
|  | SLE | 3.65 |  |  |

**Supplementary Table 2.** Correlation analyses were performed across the full set of laboratory and clinical variables

1. **Correlation between laboratory and categorical clinical variables**

| **Clinical variable** | **Laboratory variable** | **p value** | **Effect size** | **Effect**  **type** | **FDR** |
| --- | --- | --- | --- | --- | --- |
| Articular involvement | B cell CB1 MFI | 0,1534 | -0,57 | Cohen's d | 0,6570 |
| Articular involvement | B cell CB2 MFI | 0,2311 | 0,44 | Cohen's d | 0,7481 |
| Articular involvement | B eUSm CB1 MFI | 0,4115 | -0,33 | Cohen's d | 0,8417 |
| Articular involvement | B eUSm CB2 MFI | 0,1957 | 0,46 | Cohen's d | 0,7176 |
| Articular involvement | Bm1 CB1 MFI | 0,1270 | -0,57 | Cohen's d | 0,6462 |
| Articular involvement | Bm1 CB2 MFI | 0,1521 | 0,49 | Cohen's d | 0,6570 |
| Articular involvement | Bm2 CB1 MFI | 0,2526 | -0,44 | Cohen's d | 0,7572 |
| Articular involvement | Bm2 CB2 MFI | 0,3428 | 0,37 | Cohen's d | 0,8049 |
| Articular involvement | Bm2p CB1 MFI | 0,0754 | -0,78 | Cohen's d | 0,5131 |
| Articular involvement | Bm2p CB2 MFI | 0,8347 | 0,07 | Cohen's d | 0,9587 |
| Articular involvement | Bm3-4 CB1 MFI | 0,0792 | -0,77 | Cohen's d | 0,5256 |
| Articular involvement | Bm3-4 CB2 MFI | 0,1828 | 0,39 | Cohen's d | 0,6976 |
| Articular involvement | Bm5 CB1 MFI | 0,2427 | -0,47 | Cohen's d | 0,7572 |
| Articular involvement | Bm5 CB2 MFI | 0,6747 | 0,13 | Cohen's d | 0,9336 |
| Articular involvement | B Naive CB1 MFI | 0,1009 | -0,68 | Cohen's d | 0,5842 |
| Articular involvement | B Naive CB2 MFI | 0,5327 | 0,21 | Cohen's d | 0,8905 |
| Articular involvement | B Plasma cells CB1 MFI | 0,1770 | -0,47 | Cohen's d | 0,6900 |
| Articular involvement | B Plasma cells CB2 MFI | 0,7230 | 0,13 | Cohen's d | 0,9399 |
| Articular involvement | B reg CB1 MFI | 0,1143 | -0,67 | Cohen's d | 0,6286 |
| Articular involvement | B reg CB2 MFI | 0,4771 | 0,23 | Cohen's d | 0,8659 |
| Articular involvement | B Sm CB1 MFI | 0,5377 | -0,24 | Cohen's d | 0,8926 |
| Articular involvement | B Sm CB2 MFI | 0,2969 | 0,31 | Cohen's d | 0,7631 |
| Articular involvement | B Transitional  CB1 MFI | 0,0819 | -0,70 | Cohen's d | 0,5397 |
| Articular involvement | B Transitional  CB2 MFI | 0,9955 | 0,00 | Cohen's d | 0,9955 |
| Articular involvement | B USm CB1 MFI | 0,1358 | -0,64 | Cohen's d | 0,6489 |
| Articular involvement | B USm CB2 MFI | 0,2219 | 0,40 | Cohen's d | 0,7451 |
| Articular involvement | eBm5 CB1 MFI | 0,2699 | -0,46 | Cohen's d | 0,7572 |
| Articular involvement | eBm5 CB2 MFI | 0,2534 | 0,37 | Cohen's d | 0,7572 |
| Articular involvement | T cell CB1 MFI | 0,1641 | -0,50 | Cohen's d | 0,6800 |
| Articular involvement | T cell CB2 MFI | 0,0385 | 0,80 | Cohen's d | 0,3757 |
| Articular involvement | T CD4 CB1 MFI | 0,1576 | -0,54 | Cohen's d | 0,6601 |
| Articular involvement | T CD4 CB2 MFI | 0,0242 | 0,85 | Cohen's d | 0,2634 |
| Articular involvement | T CD4 Effectors CB1 MFI | 0,2262 | -0,42 | Cohen's d | 0,7451 |
| Articular involvement | T CD4 Effectors CB2 MFI | 0,1993 | 0,50 | Cohen's d | 0,7176 |
| Articular involvement | T CD4 Effectors Memory CB1 MFI | 0,1758 | -0,53 | Cohen's d | 0,6900 |
| Articular involvement | T CD4 Effectors Memory CB2 MFI | 0,0009 | 1,14 | Cohen's d | 0,0169 |
| Articular involvement | T CD4 MemoryCentral CB1 MFI | 0,1677 | -0,52 | Cohen's d | 0,6884 |
| Articular involvement | T CD4 MemoryCentral CB2 MFI | 0,0034 | 0,98 | Cohen's d | 0,0489 |
| Articular involvement | T CD4 Naive CB1 MFI | 0,1294 | -0,57 | Cohen's d | 0,6489 |
| Articular involvement | T CD4 Naive CB2 MFI | 0,0486 | 0,70 | Cohen's d | 0,4158 |
| Articular involvement | T CD8 CB1 MFI | 0,1265 | -0,51 | Cohen's d | 0,6462 |
| Articular involvement | T CD8 CB2 MFI | 0,1244 | 0,58 | Cohen's d | 0,6462 |
| Articular involvement | T CD8 Effectors CB1 MFI | 0,2843 | -0,35 | Cohen's d | 0,7631 |
| Articular involvement | T CD8 Effectors CB2 MFI | 0,0465 | 0,75 | Cohen's d | 0,4118 |
| Articular involvement | T CD8 Effectors Memory CB1 MFI | 0,2294 | -0,40 | Cohen's d | 0,7481 |
| Articular involvement | T CD8 Effectors Memory CB2 MFI | 0,0441 | 0,75 | Cohen's d | 0,3990 |
| Articular involvement | T CD8 MemoryCentral CB1 MFI | 0,1954 | -0,44 | Cohen's d | 0,7176 |
| Articular involvement | T CD8 MemoryCentral CB2 MFI | 0,0464 | 0,73 | Cohen's d | 0,4118 |
| Articular involvement | T CD8 Naive CB1 MFI | 0,0958 | -0,56 | Cohen's d | 0,5736 |
| Articular involvement | T CD8 Naive CB2 MFI | 0,0650 | 0,66 | Cohen's d | 0,5056 |
| Articular involvement | T reg CB1 MFI | 0,1076 | -0,78 | Cohen's d | 0,6887 |
| Articular involvement | T reg CB2 MFI | 0,0014 | 1,31 | Cohen's d | 0,0155 |
| Cannabis | B cell CB1 MFI | 0,9857 | -0,03 | Cohen's d | 0,9931 |
| Cannabis | B cell CB2 MFI | 0,8803 | -0,12 | Cohen's d | 0,9624 |
| Cannabis | B eUSm CB1 MFI | 0,9046 | 0,19 | Cohen's d | 0,9675 |
| Cannabis | B eUSm CB2 MFI | 0,6852 | -0,65 | Cohen's d | 0,9336 |
| Cannabis | Bm1 CB1 MFI | 0,9870 | -0,02 | Cohen's d | 0,9931 |
| Cannabis | Bm1 CB2 MFI | 0,7629 | -0,52 | Cohen's d | 0,9490 |
| Cannabis | Bm2 CB1 MFI | 0,9830 | 0,03 | Cohen's d | 0,9931 |
| Cannabis | Bm2 CB2 MFI | 0,6184 | -0,97 | Cohen's d | 0,9278 |
| Cannabis | Bm2p CB1 MFI | 0,8765 | -0,18 | Cohen's d | 0,9624 |
| Cannabis | Bm2p CB2 MFI | 0,5982 | -1,25 | Cohen's d | 0,9252 |
| Cannabis | Bm3-4 CB1 MFI | 0,9632 | -0,07 | Cohen's d | 0,9840 |
| Cannabis | Bm3-4 CB2 MFI | 0,5274 | -0,60 | Cohen's d | 0,8905 |
| Cannabis | Bm5 CB1 MFI | 0,9434 | 0,09 | Cohen's d | 0,9737 |
| Cannabis | Bm5 CB2 MFI | 0,7202 | -0,44 | Cohen's d | 0,9399 |
| Cannabis | B Naive CB1 MFI | 0,9651 | -0,06 | Cohen's d | 0,9840 |
| Cannabis | B Naive CB2 MFI | 0,6090 | -1,12 | Cohen's d | 0,9252 |
| Cannabis | B Plasma cells CB1 MFI | 0,7444 | -0,41 | Cohen's d | 0,9473 |
| Cannabis | B Plasma cells CB2 MFI | 0,5439 | -0,16 | Cohen's d | 0,8926 |
| Cannabis | B reg CB1 MFI | 0,9876 | -0,02 | Cohen's d | 0,9931 |
| Cannabis | B reg CB2 MFI | 0,6312 | -0,92 | Cohen's d | 0,9289 |
| Cannabis | B Sm CB1 MFI | 0,8544 | 0,31 | Cohen's d | 0,9624 |
| Cannabis | B Sm CB2 MFI | 0,8913 | -0,03 | Cohen's d | 0,9624 |
| Cannabis | B Transitional  CB1 MFI | 0,9040 | -0,15 | Cohen's d | 0,9675 |
| Cannabis | B Transitional  CB2 MFI | 0,6007 | -1,24 | Cohen's d | 0,9252 |
| Cannabis | B USm CB1 MFI | 0,9340 | -0,10 | Cohen's d | 0,9717 |
| Cannabis | B USm CB2 MFI | 0,8037 | -0,41 | Cohen's d | 0,9490 |
| Cannabis | eBm5 CB1 MFI | 0,8670 | 0,27 | Cohen's d | 0,9624 |
| Cannabis | eBm5 CB2 MFI | 0,6125 | -0,72 | Cohen's d | 0,9252 |
| Cannabis | T cell CB1 MFI | 0,6871 | 0,79 | Cohen's d | 0,9336 |
| Cannabis | T cell CB2 MFI | 0,3282 | -0,35 | Cohen's d | 0,7896 |
| Cannabis | T CD4 CB1 MFI | 0,6921 | 0,77 | Cohen's d | 0,9336 |
| Cannabis | T CD4 CB2 MFI | 0,2673 | -0,41 | Cohen's d | 0,7572 |
| Cannabis | T CD4 Effectors CB1 MFI | 0,6224 | 0,80 | Cohen's d | 0,9278 |
| Cannabis | T CD4 Effectors CB2 MFI | 0,7384 | 0,21 | Cohen's d | 0,9424 |
| Cannabis | T CD4 Effectors Memory CB1 MFI | 0,6952 | 0,71 | Cohen's d | 0,9336 |
| Cannabis | T CD4 Effectors Memory CB2 MFI | 0,7552 | -0,20 | Cohen's d | 0,9490 |
| Cannabis | T CD4 MemoryCentral CB1 MFI | 0,6951 | 0,74 | Cohen's d | 0,9336 |
| Cannabis | T CD4 MemoryCentral CB2 MFI | 0,2580 | -0,33 | Cohen's d | 0,7572 |
| Cannabis | T CD4 Naive CB1 MFI | 0,6730 | 0,87 | Cohen's d | 0,9336 |
| Cannabis | T CD4 Naive CB2 MFI | 0,5626 | -0,54 | Cohen's d | 0,9039 |
| Cannabis | T CD8 CB1 MFI | 0,6632 | 0,85 | Cohen's d | 0,9295 |
| Cannabis | T CD8 CB2 MFI | 0,5455 | -0,28 | Cohen's d | 0,8935 |
| Cannabis | T CD8 Effectors CB1 MFI | 0,6287 | 1,01 | Cohen's d | 0,9278 |
| Cannabis | T CD8 Effectors CB2 MFI | 0,9125 | -0,03 | Cohen's d | 0,9690 |
| Cannabis | T CD8 Effectors Memory CB1 MFI | 0,6668 | 0,95 | Cohen's d | 0,9316 |
| Cannabis | T CD8 Effectors Memory CB2 MFI | 0,6964 | 0,09 | Cohen's d | 0,9336 |
| Cannabis | T CD8 MemoryCentral CB1 MFI | 0,6476 | 0,94 | Cohen's d | 0,9295 |
| Cannabis | T CD8 MemoryCentral CB2 MFI | 0,9098 | 0,02 | Cohen's d | 0,9690 |
| Cannabis | T CD8 Naive CB1 MFI | 0,6705 | 0,78 | Cohen's d | 0,9328 |
| Cannabis | T CD8 Naive CB2 MFI | 0,4732 | -0,53 | Cohen's d | 0,8659 |
| Chloroquine/Hydroxychloroquine | B cell CB1 MFI | 0,8367 | 0,07 | Cohen's d | 0,9587 |
| Chloroquine/Hydroxychloroquine | B cell CB2 MFI | 0,7353 | -0,12 | Cohen's d | 0,9424 |
| Chloroquine/Hydroxychloroquine | B eUSm CB1 MFI | 0,5035 | 0,24 | Cohen's d | 0,8807 |
| Chloroquine/Hydroxychloroquine | B eUSm CB2 MFI | 0,8474 | -0,07 | Cohen's d | 0,9601 |
| Chloroquine/Hydroxychloroquine | Bm1 CB1 MFI | 0,6687 | 0,15 | Cohen's d | 0,9318 |
| Chloroquine/Hydroxychloroquine | Bm1 CB2 MFI | 0,7003 | -0,14 | Cohen's d | 0,9345 |
| Chloroquine/Hydroxychloroquine | Bm2 CB1 MFI | 0,4479 | 0,27 | Cohen's d | 0,8659 |
| Chloroquine/Hydroxychloroquine | Bm2 CB2 MFI | 0,7130 | 0,12 | Cohen's d | 0,9372 |
| Chloroquine/Hydroxychloroquine | Bm2p CB1 MFI | 0,6558 | 0,15 | Cohen's d | 0,9295 |
| Chloroquine/Hydroxychloroquine | Bm2p CB2 MFI | 0,9295 | -0,03 | Cohen's d | 0,9717 |
| Chloroquine/Hydroxychloroquine | Bm3-4 CB1 MFI | 0,5225 | 0,23 | Cohen's d | 0,8900 |
| Chloroquine/Hydroxychloroquine | Bm3-4 CB2 MFI | 0,4678 | -0,24 | Cohen's d | 0,8659 |
| Chloroquine/Hydroxychloroquine | Bm5 CB1 MFI | 0,4852 | -0,23 | Cohen's d | 0,8659 |
| Chloroquine/Hydroxychloroquine | Bm5 CB2 MFI | 0,2454 | -0,41 | Cohen's d | 0,7572 |
| Chloroquine/Hydroxychloroquine | B Naive CB1 MFI | 0,7139 | 0,13 | Cohen's d | 0,9372 |
| Chloroquine/Hydroxychloroquine | B Naive CB2 MFI | 0,9331 | 0,03 | Cohen's d | 0,9717 |
| Chloroquine/Hydroxychloroquine | B Plasma cells CB1 MFI | 0,5053 | 0,23 | Cohen's d | 0,8820 |
| Chloroquine/Hydroxychloroquine | B Plasma cells CB2 MFI | 0,8687 | 0,06 | Cohen's d | 0,9624 |
| Chloroquine/Hydroxychloroquine | B reg CB1 MFI | 0,7159 | 0,13 | Cohen's d | 0,9372 |
| Chloroquine/Hydroxychloroquine | B reg CB2 MFI | 0,6550 | -0,15 | Cohen's d | 0,9295 |
| Chloroquine/Hydroxychloroquine | B Sm CB1 MFI | 0,8189 | -0,08 | Cohen's d | 0,9549 |
| Chloroquine/Hydroxychloroquine | B Sm CB2 MFI | 0,2181 | -0,41 | Cohen's d | 0,7356 |
| Chloroquine/Hydroxychloroquine | B Transitional  CB1 MFI | 0,6017 | 0,18 | Cohen's d | 0,9252 |
| Chloroquine/Hydroxychloroquine | B Transitional  CB2 MFI | 0,9264 | 0,03 | Cohen's d | 0,9717 |
| Chloroquine/Hydroxychloroquine | B USm CB1 MFI | 0,6151 | 0,18 | Cohen's d | 0,9254 |
| Chloroquine/Hydroxychloroquine | B USm CB2 MFI | 0,3585 | -0,33 | Cohen's d | 0,8136 |
| Chloroquine/Hydroxychloroquine | eBm5 CB1 MFI | 0,6800 | -0,14 | Cohen's d | 0,9336 |
| Chloroquine/Hydroxychloroquine | eBm5 CB2 MFI | 0,5787 | -0,18 | Cohen's d | 0,9126 |
| Chloroquine/Hydroxychloroquine | T cell CB1 MFI | 0,2574 | -0,39 | Cohen's d | 0,7572 |
| Chloroquine/Hydroxychloroquine | T cell CB2 MFI | 0,3654 | -0,33 | Cohen's d | 0,8211 |
| Chloroquine/Hydroxychloroquine | T CD4 CB1 MFI | 0,2725 | -0,38 | Cohen's d | 0,7572 |
| Chloroquine/Hydroxychloroquine | T CD4 CB2 MFI | 0,2083 | -0,45 | Cohen's d | 0,7176 |
| Chloroquine/Hydroxychloroquine | T CD4 Effectors CB1 MFI | 0,2896 | -0,38 | Cohen's d | 0,7631 |
| Chloroquine/Hydroxychloroquine | T CD4 Effectors CB2 MFI | 0,8153 | -0,09 | Cohen's d | 0,9545 |
| Chloroquine/Hydroxychloroquine | T CD4 Effectors Memory CB1 MFI | 0,2717 | -0,39 | Cohen's d | 0,7572 |
| Chloroquine/Hydroxychloroquine | T CD4 Effectors Memory CB2 MFI | 0,0579 | -0,69 | Cohen's d | 0,4689 |
| Chloroquine/Hydroxychloroquine | T CD4 MemoryCentral CB1 MFI | 0,2409 | -0,41 | Cohen's d | 0,7572 |
| Chloroquine/Hydroxychloroquine | T CD4 MemoryCentral CB2 MFI | 0,1121 | -0,58 | Cohen's d | 0,6226 |
| Chloroquine/Hydroxychloroquine | T CD4 Naive CB1 MFI | 0,2974 | -0,36 | Cohen's d | 0,7631 |
| Chloroquine/Hydroxychloroquine | T CD4 Naive CB2 MFI | 0,8302 | 0,08 | Cohen's d | 0,9581 |
| Chloroquine/Hydroxychloroquine | T CD8 CB1 MFI | 0,2245 | -0,42 | Cohen's d | 0,7451 |
| Chloroquine/Hydroxychloroquine | T CD8 CB2 MFI | 0,9415 | -0,03 | Cohen's d | 0,9736 |
| Chloroquine/Hydroxychloroquine | T CD8 Effectors CB1 MFI | 0,1561 | -0,49 | Cohen's d | 0,6570 |
| Chloroquine/Hydroxychloroquine | T CD8 Effectors CB2 MFI | 0,6055 | -0,18 | Cohen's d | 0,9252 |
| Chloroquine/Hydroxychloroquine | T CD8 Effectors Memory CB1 MFI | 0,1738 | -0,47 | Cohen's d | 0,6900 |
| Chloroquine/Hydroxychloroquine | T CD8 Effectors Memory CB2 MFI | 0,6619 | -0,16 | Cohen's d | 0,9295 |
| Chloroquine/Hydroxychloroquine | T CD8 MemoryCentral CB1 MFI | 0,1896 | -0,45 | Cohen's d | 0,7158 |
| Chloroquine/Hydroxychloroquine | T CD8 MemoryCentral CB2 MFI | 0,4539 | -0,27 | Cohen's d | 0,8659 |
| Chloroquine/Hydroxychloroquine | T CD8 Naive CB1 MFI | 0,2866 | -0,37 | Cohen's d | 0,7631 |
| Chloroquine/Hydroxychloroquine | T CD8 Naive CB2 MFI | 0,8946 | -0,05 | Cohen's d | 0,9624 |
| Chloroquine/Hydroxychloroquine | T reg CB1 MFI | 0,4907 | -0,28 | Cohen's d | 0,7477 |
| Chloroquine/Hydroxychloroquine | T reg CB2 MFI | 0,2415 | -0,48 | Cohen's d | 0,6943 |
| Constitutional involvement | B cell CB1 MFI | 0,2414 | 0,67 | Cohen's d | 0,7572 |
| Constitutional involvement | B cell CB2 MFI | 0,4745 | 0,32 | Cohen's d | 0,8659 |
| Constitutional involvement | B eUSm CB1 MFI | 0,1544 | 0,90 | Cohen's d | 0,6570 |
| Constitutional involvement | B eUSm CB2 MFI | 0,8893 | 0,09 | Cohen's d | 0,9624 |
| Constitutional involvement | Bm1 CB1 MFI | 0,2915 | 0,60 | Cohen's d | 0,7631 |
| Constitutional involvement | Bm1 CB2 MFI | 0,9376 | 0,05 | Cohen's d | 0,9717 |
| Constitutional involvement | Bm2 CB1 MFI | 0,1838 | 0,76 | Cohen's d | 0,6976 |
| Constitutional involvement | Bm2 CB2 MFI | 0,7897 | -0,19 | Cohen's d | 0,9490 |
| Constitutional involvement | Bm2p CB1 MFI | 0,3855 | 0,40 | Cohen's d | 0,8278 |
| Constitutional involvement | Bm2p CB2 MFI | 0,5093 | -0,49 | Cohen's d | 0,8823 |
| Constitutional involvement | Bm3-4 CB1 MFI | 0,2841 | 0,68 | Cohen's d | 0,7631 |
| Constitutional involvement | Bm3-4 CB2 MFI | 0,7852 | -0,10 | Cohen's d | 0,9490 |
| Constitutional involvement | Bm5 CB1 MFI | 0,2824 | 0,52 | Cohen's d | 0,7631 |
| Constitutional involvement | Bm5 CB2 MFI | 0,8059 | 0,12 | Cohen's d | 0,9490 |
| Constitutional involvement | B Naive CB1 MFI | 0,2798 | 0,56 | Cohen's d | 0,7631 |
| Constitutional involvement | B Naive CB2 MFI | 0,6990 | -0,30 | Cohen's d | 0,9345 |
| Constitutional involvement | B Plasma cells CB1 MFI | 0,5997 | 0,24 | Cohen's d | 0,9252 |
| Constitutional involvement | B Plasma cells CB2 MFI | 0,4009 | 0,51 | Cohen's d | 0,8414 |
| Constitutional involvement | B reg CB1 MFI | 0,2609 | 0,56 | Cohen's d | 0,7572 |
| Constitutional involvement | B reg CB2 MFI | 0,6594 | -0,29 | Cohen's d | 0,9295 |
| Constitutional involvement | B Sm CB1 MFI | 0,2148 | 0,74 | Cohen's d | 0,7301 |
| Constitutional involvement | B Sm CB2 MFI | 0,2661 | 0,30 | Cohen's d | 0,7572 |
| Constitutional involvement | B Transitional  CB1 MFI | 0,3591 | 0,42 | Cohen's d | 0,8136 |
| Constitutional involvement | B Transitional  CB2 MFI | 0,4614 | -0,57 | Cohen's d | 0,8659 |
| Constitutional involvement | B USm CB1 MFI | 0,2844 | 0,54 | Cohen's d | 0,7631 |
| Constitutional involvement | B USm CB2 MFI | 0,8594 | 0,11 | Cohen's d | 0,9624 |
| Constitutional involvement | eBm5 CB1 MFI | 0,2070 | 0,76 | Cohen's d | 0,7176 |
| Constitutional involvement | eBm5 CB2 MFI | 0,8483 | 0,10 | Cohen's d | 0,9601 |
| Constitutional involvement | T cell CB1 MFI | 0,1648 | 0,83 | Cohen's d | 0,6800 |
| Constitutional involvement | T cell CB2 MFI | 0,3046 | -0,31 | Cohen's d | 0,7684 |
| Constitutional involvement | T CD4 CB1 MFI | 0,1431 | 0,86 | Cohen's d | 0,6562 |
| Constitutional involvement | T CD4 CB2 MFI | 0,1466 | -0,40 | Cohen's d | 0,6570 |
| Constitutional involvement | T CD4 Effectors CB1 MFI | 0,2350 | 0,77 | Cohen's d | 0,7566 |
| Constitutional involvement | T CD4 Effectors CB2 MFI | 0,0420 | -0,66 | Cohen's d | 0,3909 |
| Constitutional involvement | T CD4 Effectors Memory CB1 MFI | 0,1837 | 0,74 | Cohen's d | 0,6976 |
| Constitutional involvement | T CD4 Effectors Memory CB2 MFI | 0,7755 | 0,10 | Cohen's d | 0,9490 |
| Constitutional involvement | T CD4 MemoryCentral CB1 MFI | 0,1438 | 0,87 | Cohen's d | 0,6562 |
| Constitutional involvement | T CD4 MemoryCentral CB2 MFI | 0,7505 | -0,09 | Cohen's d | 0,9490 |
| Constitutional involvement | T CD4 Naive CB1 MFI | 0,1385 | 0,93 | Cohen's d | 0,6506 |
| Constitutional involvement | T CD4 Naive CB2 MFI | 0,1767 | -0,55 | Cohen's d | 0,6900 |
| Constitutional involvement | T CD8 CB1 MFI | 0,2376 | 0,71 | Cohen's d | 0,7572 |
| Constitutional involvement | T CD8 CB2 MFI | 0,2751 | -0,43 | Cohen's d | 0,7582 |
| Constitutional involvement | T CD8 Effectors CB1 MFI | 0,2965 | 0,69 | Cohen's d | 0,7631 |
| Constitutional involvement | T CD8 Effectors CB2 MFI | 0,2511 | -0,33 | Cohen's d | 0,7572 |
| Constitutional involvement | T CD8 Effectors Memory CB1 MFI | 0,3211 | 0,63 | Cohen's d | 0,7842 |
| Constitutional involvement | T CD8 Effectors Memory CB2 MFI | 0,2988 | -0,34 | Cohen's d | 0,7631 |
| Constitutional involvement | T CD8 MemoryCentral CB1 MFI | 0,2603 | 0,72 | Cohen's d | 0,7572 |
| Constitutional involvement | T CD8 MemoryCentral CB2 MFI | 0,7275 | -0,10 | Cohen's d | 0,9399 |
| Constitutional involvement | T CD8 Naive CB1 MFI | 0,1729 | 0,77 | Cohen's d | 0,6900 |
| Constitutional involvement | T CD8 Naive CB2 MFI | 0,3569 | -0,39 | Cohen's d | 0,8136 |
| Constitutional involvement | T reg CB1 MFI | 0,3043 | 0,63 | Cohen's d | 0,6943 |
| Constitutional involvement | T reg CB2 MFI | 0,9653 | -0,01 | Cohen's d | 0,9653 |
| hematological involvement | B cell CB1 MFI | 0,6086 | 0,19 | Cohen's d | 0,9252 |
| hematological involvement | B cell CB2 MFI | 0,5073 | 0,27 | Cohen's d | 0,8823 |
| hematological involvement | B eUSm CB1 MFI | 0,8018 | 0,09 | Cohen's d | 0,9490 |
| hematological involvement | B eUSm CB2 MFI | 0,7681 | 0,10 | Cohen's d | 0,9490 |
| hematological involvement | Bm1 CB1 MFI | 0,8430 | 0,07 | Cohen's d | 0,9592 |
| hematological involvement | Bm1 CB2 MFI | 0,7900 | 0,10 | Cohen's d | 0,9490 |
| hematological involvement | Bm2 CB1 MFI | 0,7937 | 0,10 | Cohen's d | 0,9490 |
| hematological involvement | Bm2 CB2 MFI | 0,9682 | -0,01 | Cohen's d | 0,9840 |
| hematological involvement | Bm2p CB1 MFI | 0,8016 | 0,09 | Cohen's d | 0,9490 |
| hematological involvement | Bm2p CB2 MFI | 0,8670 | -0,06 | Cohen's d | 0,9624 |
| hematological involvement | Bm3-4 CB1 MFI | 0,7298 | 0,12 | Cohen's d | 0,9399 |
| hematological involvement | Bm3-4 CB2 MFI | 0,3387 | 0,28 | Cohen's d | 0,8036 |
| hematological involvement | Bm5 CB1 MFI | 0,8910 | -0,06 | Cohen's d | 0,9624 |
| hematological involvement | Bm5 CB2 MFI | 0,9706 | -0,01 | Cohen's d | 0,9840 |
| hematological involvement | B Naive CB1 MFI | 0,6881 | 0,15 | Cohen's d | 0,9336 |
| hematological involvement | B Naive CB2 MFI | 0,9651 | 0,02 | Cohen's d | 0,9840 |
| hematological involvement | B Plasma cells CB1 MFI | 0,7846 | 0,10 | Cohen's d | 0,9490 |
| hematological involvement | B Plasma cells CB2 MFI | 0,1489 | 0,40 | Cohen's d | 0,6570 |
| hematological involvement | B reg CB1 MFI | 0,7484 | 0,11 | Cohen's d | 0,9490 |
| hematological involvement | B reg CB2 MFI | 0,9166 | -0,04 | Cohen's d | 0,9697 |
| hematological involvement | B Sm CB1 MFI | 0,4073 | 0,31 | Cohen's d | 0,8417 |
| hematological involvement | B Sm CB2 MFI | 0,4805 | 0,21 | Cohen's d | 0,8659 |
| hematological involvement | B Transitional  CB1 MFI | 0,8570 | 0,07 | Cohen's d | 0,9624 |
| hematological involvement | B Transitional  CB2 MFI | 0,9131 | -0,04 | Cohen's d | 0,9690 |
| hematological involvement | B USm CB1 MFI | 0,9345 | 0,03 | Cohen's d | 0,9717 |
| hematological involvement | B USm CB2 MFI | 0,8901 | -0,05 | Cohen's d | 0,9624 |
| hematological involvement | eBm5 CB1 MFI | 0,6344 | 0,17 | Cohen's d | 0,9289 |
| hematological involvement | eBm5 CB2 MFI | 0,4692 | 0,23 | Cohen's d | 0,8659 |
| hematological involvement | T cell CB1 MFI | 0,6535 | 0,21 | Cohen's d | 0,9295 |
| hematological involvement | T cell CB2 MFI | 0,1630 | -0,58 | Cohen's d | 0,6791 |
| hematological involvement | T CD4 CB1 MFI | 0,6128 | 0,23 | Cohen's d | 0,9252 |
| hematological involvement | T CD4 CB2 MFI | 0,2241 | -0,47 | Cohen's d | 0,7451 |
| hematological involvement | T CD4 Effectors CB1 MFI | 0,6998 | 0,17 | Cohen's d | 0,9345 |
| hematological involvement | T CD4 Effectors CB2 MFI | 0,4385 | -0,24 | Cohen's d | 0,8659 |
| hematological involvement | T CD4 Effectors Memory CB1 MFI | 0,6139 | 0,22 | Cohen's d | 0,9252 |
| hematological involvement | T CD4 Effectors Memory CB2 MFI | 0,3767 | -0,35 | Cohen's d | 0,8217 |
| hematological involvement | T CD4 MemoryCentral CB1 MFI | 0,6279 | 0,22 | Cohen's d | 0,9278 |
| hematological involvement | T CD4 MemoryCentral CB2 MFI | 0,5156 | -0,22 | Cohen's d | 0,8843 |
| hematological involvement | T CD4 Naive CB1 MFI | 0,5482 | 0,26 | Cohen's d | 0,8943 |
| hematological involvement | T CD4 Naive CB2 MFI | 0,2765 | -0,42 | Cohen's d | 0,7582 |
| hematological involvement | T CD8 CB1 MFI | 0,7967 | 0,13 | Cohen's d | 0,9490 |
| hematological involvement | T CD8 CB2 MFI | 0,2142 | -0,59 | Cohen's d | 0,7301 |
| hematological involvement | T CD8 Effectors CB1 MFI | 0,8658 | 0,08 | Cohen's d | 0,9624 |
| hematological involvement | T CD8 Effectors CB2 MFI | 0,3151 | -0,51 | Cohen's d | 0,7786 |
| hematological involvement | T CD8 Effectors Memory CB1 MFI | 0,8898 | 0,07 | Cohen's d | 0,9624 |
| hematological involvement | T CD8 Effectors Memory CB2 MFI | 0,2998 | -0,48 | Cohen's d | 0,7631 |
| hematological involvement | T CD8 MemoryCentral CB1 MFI | 0,8068 | 0,11 | Cohen's d | 0,9490 |
| hematological involvement | T CD8 MemoryCentral CB2 MFI | 0,3708 | -0,38 | Cohen's d | 0,8211 |
| hematological involvement | T CD8 Naive CB1 MFI | 0,7297 | 0,17 | Cohen's d | 0,9399 |
| hematological involvement | T CD8 Naive CB2 MFI | 0,1520 | -0,69 | Cohen's d | 0,6570 |
| hematological involvement | T reg CB1 MFI | 0,6375 | 0,24 | Cohen's d | 0,8160 |
| hematological involvement | T reg CB2 MFI | 0,4091 | -0,30 | Cohen's d | 0,6943 |
| Immune involvement | B cell CB1 MFI | 0,0845 | -0,70 | Cohen's d | 0,5444 |
| Immune involvement | B cell CB2 MFI | 0,1054 | 0,62 | Cohen's d | 0,5972 |
| Immune involvement | B eUSm CB1 MFI | 0,0490 | -0,87 | Cohen's d | 0,4158 |
| Immune involvement | B eUSm CB2 MFI | 0,1334 | 0,66 | Cohen's d | 0,6489 |
| Immune involvement | Bm1 CB1 MFI | 0,0675 | -0,69 | Cohen's d | 0,5056 |
| Immune involvement | Bm1 CB2 MFI | 0,0667 | 0,86 | Cohen's d | 0,5056 |
| Immune involvement | Bm2 CB1 MFI | 0,0761 | -0,71 | Cohen's d | 0,5131 |
| Immune involvement | Bm2 CB2 MFI | 0,0882 | 0,77 | Cohen's d | 0,5622 |
| Immune involvement | Bm2p CB1 MFI | 0,1373 | -0,52 | Cohen's d | 0,6489 |
| Immune involvement | Bm2p CB2 MFI | 0,0423 | 1,01 | Cohen's d | 0,3909 |
| Immune involvement | Bm3-4 CB1 MFI | 0,1951 | -0,48 | Cohen's d | 0,7176 |
| Immune involvement | Bm3-4 CB2 MFI | 0,0745 | 0,53 | Cohen's d | 0,5131 |
| Immune involvement | Bm5 CB1 MFI | 0,0730 | -0,87 | Cohen's d | 0,5131 |
| Immune involvement | Bm5 CB2 MFI | 0,0719 | 0,62 | Cohen's d | 0,5131 |
| Immune involvement | B Naive CB1 MFI | 0,1444 | -0,54 | Cohen's d | 0,6562 |
| Immune involvement | B Naive CB2 MFI | 0,0688 | 0,90 | Cohen's d | 0,5086 |
| Immune involvement | B Plasma cells CB1 MFI | 0,2315 | -0,43 | Cohen's d | 0,7481 |
| Immune involvement | B Plasma cells CB2 MFI | 0,0709 | 0,49 | Cohen's d | 0,5131 |
| Immune involvement | B reg CB1 MFI | 0,1440 | -0,53 | Cohen's d | 0,6562 |
| Immune involvement | B reg CB2 MFI | 0,0214 | 1,11 | Cohen's d | 0,2488 |
| Immune involvement | B Sm CB1 MFI | 0,0989 | -0,76 | Cohen's d | 0,5842 |
| Immune involvement | B Sm CB2 MFI | 0,1412 | 0,44 | Cohen's d | 0,6557 |
| Immune involvement | B Transitional  CB1 MFI | 0,1736 | -0,47 | Cohen's d | 0,6900 |
| Immune involvement | B Transitional  CB2 MFI | 0,0408 | 1,02 | Cohen's d | 0,3850 |
| Immune involvement | B USm CB1 MFI | 0,0676 | -0,87 | Cohen's d | 0,5056 |
| Immune involvement | B USm CB2 MFI | 0,1529 | 0,65 | Cohen's d | 0,6570 |
| Immune involvement | eBm5 CB1 MFI | 0,0709 | -0,86 | Cohen's d | 0,5131 |
| Immune involvement | eBm5 CB2 MFI | 0,1237 | 0,56 | Cohen's d | 0,6462 |
| Immune involvement | T cell CB1 MFI | 0,0757 | -0,97 | Cohen's d | 0,5131 |
| Immune involvement | T cell CB2 MFI | 0,4068 | 0,29 | Cohen's d | 0,8417 |
| Immune involvement | T CD4 CB1 MFI | 0,0620 | -1,02 | Cohen's d | 0,4927 |
| Immune involvement | T CD4 CB2 MFI | 0,5403 | 0,21 | Cohen's d | 0,8926 |
| Immune involvement | T CD4 Effectors CB1 MFI | 0,0928 | -0,91 | Cohen's d | 0,5677 |
| Immune involvement | T CD4 Effectors CB2 MFI | 0,5161 | 0,24 | Cohen's d | 0,8843 |
| Immune involvement | T CD4 Effectors Memory CB1 MFI | 0,1000 | -0,90 | Cohen's d | 0,5842 |
| Immune involvement | T CD4 Effectors Memory CB2 MFI | 0,6117 | -0,18 | Cohen's d | 0,9252 |
| Immune involvement | T CD4 MemoryCentral CB1 MFI | 0,0713 | -0,98 | Cohen's d | 0,5131 |
| Immune involvement | T CD4 MemoryCentral CB2 MFI | 0,8951 | 0,05 | Cohen's d | 0,9624 |
| Immune involvement | T CD4 Naive CB1 MFI | 0,0494 | -1,07 | Cohen's d | 0,4158 |
| Immune involvement | T CD4 Naive CB2 MFI | 0,0678 | 0,65 | Cohen's d | 0,5056 |
| Immune involvement | T CD8 CB1 MFI | 0,1256 | -0,83 | Cohen's d | 0,6462 |
| Immune involvement | T CD8 CB2 MFI | 0,1470 | 0,51 | Cohen's d | 0,6570 |
| Immune involvement | T CD8 Effectors CB1 MFI | 0,1345 | -0,84 | Cohen's d | 0,6489 |
| Immune involvement | T CD8 Effectors CB2 MFI | 0,1960 | 0,44 | Cohen's d | 0,7176 |
| Immune involvement | T CD8 Effectors Memory CB1 MFI | 0,1472 | -0,79 | Cohen's d | 0,6570 |
| Immune involvement | T CD8 Effectors Memory CB2 MFI | 0,3483 | 0,34 | Cohen's d | 0,8112 |
| Immune involvement | T CD8 MemoryCentral CB1 MFI | 0,1219 | -0,85 | Cohen's d | 0,6462 |
| Immune involvement | T CD8 MemoryCentral CB2 MFI | 0,8745 | 0,06 | Cohen's d | 0,9624 |
| Immune involvement | T CD8 Naive CB1 MFI | 0,0945 | -0,89 | Cohen's d | 0,5736 |
| Immune involvement | T CD8 Naive CB2 MFI | 0,1251 | 0,52 | Cohen's d | 0,6462 |
| Immune involvement | T reg CB1 MFI | 0,0931 | -0,93 | Cohen's d | 0,6887 |
| Immune involvement | T reg CB2 MFI | 0,9388 | -0,03 | Cohen's d | 0,9653 |
| Lupus nephritis | B cell CB1 MFI | 0,5125 | -0,23 | Cohen's d | 0,8843 |
| Lupus nephritis | B cell CB2 MFI | 0,7125 | -0,13 | Cohen's d | 0,9372 |
| Lupus nephritis | B eUSm CB1 MFI | 0,8813 | -0,05 | Cohen's d | 0,9624 |
| Lupus nephritis | B eUSm CB2 MFI | 0,7236 | -0,13 | Cohen's d | 0,9399 |
| Lupus nephritis | Bm1 CB1 MFI | 0,5907 | -0,19 | Cohen's d | 0,9230 |
| Lupus nephritis | Bm1 CB2 MFI | 0,4848 | -0,25 | Cohen's d | 0,8659 |
| Lupus nephritis | Bm2 CB1 MFI | 0,7813 | -0,10 | Cohen's d | 0,9490 |
| Lupus nephritis | Bm2 CB2 MFI | 0,7139 | -0,13 | Cohen's d | 0,9372 |
| Lupus nephritis | Bm2p CB1 MFI | 0,4936 | -0,25 | Cohen's d | 0,8755 |
| Lupus nephritis | Bm2p CB2 MFI | 0,6211 | -0,18 | Cohen's d | 0,9278 |
| Lupus nephritis | Bm3-4 CB1 MFI | 0,4665 | -0,26 | Cohen's d | 0,8659 |
| Lupus nephritis | Bm3-4 CB2 MFI | 0,7645 | 0,11 | Cohen's d | 0,9490 |
| Lupus nephritis | Bm5 CB1 MFI | 0,7114 | -0,13 | Cohen's d | 0,9372 |
| Lupus nephritis | Bm5 CB2 MFI | 0,7849 | -0,10 | Cohen's d | 0,9490 |
| Lupus nephritis | B Naive CB1 MFI | 0,5379 | -0,22 | Cohen's d | 0,8926 |
| Lupus nephritis | B Naive CB2 MFI | 0,5536 | -0,21 | Cohen's d | 0,8997 |
| Lupus nephritis | B Plasma cells CB1 MFI | 0,4229 | -0,29 | Cohen's d | 0,8538 |
| Lupus nephritis | B Plasma cells CB2 MFI | 0,6431 | 0,17 | Cohen's d | 0,9295 |
| Lupus nephritis | B reg CB1 MFI | 0,5471 | -0,22 | Cohen's d | 0,8942 |
| Lupus nephritis | B reg CB2 MFI | 0,7465 | -0,12 | Cohen's d | 0,9485 |
| Lupus nephritis | B Sm CB1 MFI | 0,8401 | -0,07 | Cohen's d | 0,9591 |
| Lupus nephritis | B Sm CB2 MFI | 0,9209 | -0,04 | Cohen's d | 0,9713 |
| Lupus nephritis | B Transitional  CB1 MFI | 0,5740 | -0,20 | Cohen's d | 0,9113 |
| Lupus nephritis | B Transitional  CB2 MFI | 0,7606 | -0,11 | Cohen's d | 0,9490 |
| Lupus nephritis | B USm CB1 MFI | 0,7622 | -0,11 | Cohen's d | 0,9490 |
| Lupus nephritis | B USm CB2 MFI | 0,4382 | -0,28 | Cohen's d | 0,8659 |
| Lupus nephritis | eBm5 CB1 MFI | 0,6808 | -0,15 | Cohen's d | 0,9336 |
| Lupus nephritis | eBm5 CB2 MFI | 0,7516 | -0,11 | Cohen's d | 0,9490 |
| Lupus nephritis | T cell CB1 MFI | 0,7445 | 0,12 | Cohen's d | 0,9473 |
| Lupus nephritis | T cell CB2 MFI | 0,7971 | -0,09 | Cohen's d | 0,9490 |
| Lupus nephritis | T CD4 CB1 MFI | 0,7248 | 0,13 | Cohen's d | 0,9399 |
| Lupus nephritis | T CD4 CB2 MFI | 0,3591 | -0,33 | Cohen's d | 0,8136 |
| Lupus nephritis | T CD4 Effectors CB1 MFI | 0,8179 | 0,08 | Cohen's d | 0,9549 |
| Lupus nephritis | T CD4 Effectors CB2 MFI | 0,5134 | 0,23 | Cohen's d | 0,8843 |
| Lupus nephritis | T CD4 Effectors Memory CB1 MFI | 0,8060 | 0,09 | Cohen's d | 0,9490 |
| Lupus nephritis | T CD4 Effectors Memory CB2 MFI | 0,7938 | -0,09 | Cohen's d | 0,9490 |
| Lupus nephritis | T CD4 MemoryCentral CB1 MFI | 0,7990 | 0,09 | Cohen's d | 0,9490 |
| Lupus nephritis | T CD4 MemoryCentral CB2 MFI | 0,6102 | -0,18 | Cohen's d | 0,9252 |
| Lupus nephritis | T CD4 Naive CB1 MFI | 0,7294 | 0,12 | Cohen's d | 0,9399 |
| Lupus nephritis | T CD4 Naive CB2 MFI | 0,7905 | -0,09 | Cohen's d | 0,9490 |
| Lupus nephritis | T CD8 CB1 MFI | 0,7379 | 0,12 | Cohen's d | 0,9424 |
| Lupus nephritis | T CD8 CB2 MFI | 0,4313 | 0,28 | Cohen's d | 0,8577 |
| Lupus nephritis | T CD8 Effectors CB1 MFI | 0,7243 | 0,13 | Cohen's d | 0,9399 |
| Lupus nephritis | T CD8 Effectors CB2 MFI | 0,4596 | 0,27 | Cohen's d | 0,8659 |
| Lupus nephritis | T CD8 Effectors Memory CB1 MFI | 0,7497 | 0,11 | Cohen's d | 0,9490 |
| Lupus nephritis | T CD8 Effectors Memory CB2 MFI | 0,4529 | 0,27 | Cohen's d | 0,8659 |
| Lupus nephritis | T CD8 MemoryCentral CB1 MFI | 0,7819 | 0,10 | Cohen's d | 0,9490 |
| Lupus nephritis | T CD8 MemoryCentral CB2 MFI | 0,7896 | 0,10 | Cohen's d | 0,9490 |
| Lupus nephritis | T CD8 Naive CB1 MFI | 0,7636 | 0,11 | Cohen's d | 0,9490 |
| Lupus nephritis | T CD8 Naive CB2 MFI | 0,4159 | 0,29 | Cohen's d | 0,8417 |
| Lupus nephritis | T reg CB1 MFI | 0,2074 | 0,56 | Cohen's d | 0,6943 |
| Lupus nephritis | T reg CB2 MFI | 0,4235 | -0,33 | Cohen's d | 0,6943 |
| Mucosal involvement | B cell CB1 MFI | 0,4617 | 0,67 | Cohen's d | 0,8659 |
| Mucosal involvement | B cell CB2 MFI | 0,9916 | -0,01 | Cohen's d | 0,9931 |
| Mucosal involvement | B eUSm CB1 MFI | 0,3618 | 0,87 | Cohen's d | 0,8158 |
| Mucosal involvement | B eUSm CB2 MFI | 0,6823 | -0,41 | Cohen's d | 0,9336 |
| Mucosal involvement | Bm1 CB1 MFI | 0,4531 | 0,55 | Cohen's d | 0,8659 |
| Mucosal involvement | Bm1 CB2 MFI | 0,6674 | -0,46 | Cohen's d | 0,9316 |
| Mucosal involvement | Bm2 CB1 MFI | 0,4798 | 0,59 | Cohen's d | 0,8659 |
| Mucosal involvement | Bm2 CB2 MFI | 0,5019 | -0,81 | Cohen's d | 0,8800 |
| Mucosal involvement | Bm2p CB1 MFI | 0,7273 | 0,24 | Cohen's d | 0,9399 |
| Mucosal involvement | Bm2p CB2 MFI | 0,4559 | -1,02 | Cohen's d | 0,8659 |
| Mucosal involvement | Bm3-4 CB1 MFI | 0,5885 | 0,42 | Cohen's d | 0,9213 |
| Mucosal involvement | Bm3-4 CB2 MFI | 0,4779 | -0,39 | Cohen's d | 0,8659 |
| Mucosal involvement | Bm5 CB1 MFI | 0,4144 | 0,66 | Cohen's d | 0,8417 |
| Mucosal involvement | Bm5 CB2 MFI | 0,6543 | -0,33 | Cohen's d | 0,9295 |
| Mucosal involvement | B Naive CB1 MFI | 0,5654 | 0,42 | Cohen's d | 0,9039 |
| Mucosal involvement | B Naive CB2 MFI | 0,4686 | -0,97 | Cohen's d | 0,8659 |
| Mucosal involvement | B Plasma cells CB1 MFI | 0,8956 | 0,09 | Cohen's d | 0,9624 |
| Mucosal involvement | B Plasma cells CB2 MFI | 0,4690 | -0,16 | Cohen's d | 0,8659 |
| Mucosal involvement | B reg CB1 MFI | 0,5798 | 0,43 | Cohen's d | 0,9127 |
| Mucosal involvement | B reg CB2 MFI | 0,4562 | -0,84 | Cohen's d | 0,8659 |
| Mucosal involvement | B Sm CB1 MFI | 0,3959 | 0,92 | Cohen's d | 0,8351 |
| Mucosal involvement | B Sm CB2 MFI | 0,6831 | 0,10 | Cohen's d | 0,9336 |
| Mucosal involvement | B Transitional  CB1 MFI | 0,7157 | 0,25 | Cohen's d | 0,9372 |
| Mucosal involvement | B Transitional  CB2 MFI | 0,4114 | -1,14 | Cohen's d | 0,8417 |
| Mucosal involvement | B USm CB1 MFI | 0,4708 | 0,48 | Cohen's d | 0,8659 |
| Mucosal involvement | B USm CB2 MFI | 0,6881 | -0,42 | Cohen's d | 0,9336 |
| Mucosal involvement | eBm5 CB1 MFI | 0,3882 | 0,90 | Cohen's d | 0,8281 |
| Mucosal involvement | eBm5 CB2 MFI | 0,6508 | -0,37 | Cohen's d | 0,9295 |
| Mucosal involvement | T cell CB1 MFI | 0,2001 | 1,29 | Cohen's d | 0,7176 |
| Mucosal involvement | T cell CB2 MFI | 0,0010 | -0,85 | Cohen's d | 0,0170 |
| Mucosal involvement | T CD4 CB1 MFI | 0,2034 | 1,27 | Cohen's d | 0,7176 |
| Mucosal involvement | T CD4 CB2 MFI | 0,0005 | -0,80 | Cohen's d | 0,0115 |
| Mucosal involvement | T CD4 Effectors CB1 MFI | 0,1398 | 1,52 | Cohen's d | 0,6528 |
| Mucosal involvement | T CD4 Effectors CB2 MFI | 0,0996 | -0,73 | Cohen's d | 0,5842 |
| Mucosal involvement | T CD4 Effectors Memory CB1 MFI | 0,2011 | 1,18 | Cohen's d | 0,7176 |
| Mucosal involvement | T CD4 Effectors Memory CB2 MFI | 0,4430 | -0,30 | Cohen's d | 0,8659 |
| Mucosal involvement | T CD4 MemoryCentral CB1 MFI | 0,2019 | 1,28 | Cohen's d | 0,7176 |
| Mucosal involvement | T CD4 MemoryCentral CB2 MFI | 0,0913 | -0,50 | Cohen's d | 0,5622 |
| Mucosal involvement | T CD4 Naive CB1 MFI | 0,2032 | 1,37 | Cohen's d | 0,7176 |
| Mucosal involvement | T CD4 Naive CB2 MFI | 0,1318 | -0,86 | Cohen's d | 0,6489 |
| Mucosal involvement | T CD8 CB1 MFI | 0,1711 | 1,34 | Cohen's d | 0,6900 |
| Mucosal involvement | T CD8 CB2 MFI | 0,0004 | -0,87 | Cohen's d | 0,0111 |
| Mucosal involvement | T CD8 Effectors CB1 MFI | 0,1732 | 1,48 | Cohen's d | 0,6900 |
| Mucosal involvement | T CD8 Effectors CB2 MFI | 0,2085 | -0,58 | Cohen's d | 0,7176 |
| Mucosal involvement | T CD8 Effectors Memory CB1 MFI | 0,2393 | 1,26 | Cohen's d | 0,7572 |
| Mucosal involvement | T CD8 Effectors Memory CB2 MFI | 0,2690 | -0,54 | Cohen's d | 0,7572 |
| Mucosal involvement | T CD8 MemoryCentral CB1 MFI | 0,1993 | 1,36 | Cohen's d | 0,7176 |
| Mucosal involvement | T CD8 MemoryCentral CB2 MFI | 0,2071 | -0,49 | Cohen's d | 0,7176 |
| Mucosal involvement | T CD8 Naive CB1 MFI | 0,1529 | 1,29 | Cohen's d | 0,6570 |
| Mucosal involvement | T CD8 Naive CB2 MFI | 0,0005 | -0,98 | Cohen's d | 0,0112 |
| Mucosal involvement | T reg CB1 MFI | 0,3307 | 0,82 | Cohen's d | 0,6943 |
| Mucosal involvement | T reg CB2 MFI | 0,2821 | -0,27 | Cohen's d | 0,6943 |
| Mycophenolate Mofetil | B cell CB1 MFI | 0,4298 | -0,30 | Cohen's d | 0,8577 |
| Mycophenolate Mofetil | B cell CB2 MFI | 0,0397 | 0,79 | Cohen's d | 0,3832 |
| Mycophenolate Mofetil | B eUSm CB1 MFI | 0,3097 | -0,39 | Cohen's d | 0,7720 |
| Mycophenolate Mofetil | B eUSm CB2 MFI | 0,0363 | 0,80 | Cohen's d | 0,3627 |
| Mycophenolate Mofetil | Bm1 CB1 MFI | 0,4063 | -0,32 | Cohen's d | 0,8417 |
| Mycophenolate Mofetil | Bm1 CB2 MFI | 0,0128 | 0,95 | Cohen's d | 0,1578 |
| Mycophenolate Mofetil | Bm2 CB1 MFI | 0,3071 | -0,39 | Cohen's d | 0,7702 |
| Mycophenolate Mofetil | Bm2 CB2 MFI | 0,0839 | 0,65 | Cohen's d | 0,5444 |
| Mycophenolate Mofetil | Bm2p CB1 MFI | 0,5097 | -0,26 | Cohen's d | 0,8823 |
| Mycophenolate Mofetil | Bm2p CB2 MFI | 0,0283 | 0,80 | Cohen's d | 0,2968 |
| Mycophenolate Mofetil | Bm3-4 CB1 MFI | 0,3586 | -0,36 | Cohen's d | 0,8136 |
| Mycophenolate Mofetil | Bm3-4 CB2 MFI | 0,1041 | 0,67 | Cohen's d | 0,5941 |
| Mycophenolate Mofetil | Bm5 CB1 MFI | 0,4535 | -0,28 | Cohen's d | 0,8659 |
| Mycophenolate Mofetil | Bm5 CB2 MFI | 0,1154 | 0,62 | Cohen's d | 0,6286 |
| Mycophenolate Mofetil | B Naive CB1 MFI | 0,5749 | -0,22 | Cohen's d | 0,9113 |
| Mycophenolate Mofetil | B Naive CB2 MFI | 0,0141 | 0,91 | Cohen's d | 0,1711 |
| Mycophenolate Mofetil | B Plasma cells CB1 MFI | 0,3025 | -0,41 | Cohen's d | 0,7653 |
| Mycophenolate Mofetil | B Plasma cells CB2 MFI | 0,0669 | 0,80 | Cohen's d | 0,5056 |
| Mycophenolate Mofetil | B reg CB1 MFI | 0,4842 | -0,27 | Cohen's d | 0,8659 |
| Mycophenolate Mofetil | B reg CB2 MFI | 0,0217 | 0,87 | Cohen's d | 0,2496 |
| Mycophenolate Mofetil | B Sm CB1 MFI | 0,5158 | -0,24 | Cohen's d | 0,8843 |
| Mycophenolate Mofetil | B Sm CB2 MFI | 0,2518 | 0,46 | Cohen's d | 0,7572 |
| Mycophenolate Mofetil | B Transitional  CB1 MFI | 0,3717 | -0,35 | Cohen's d | 0,8211 |
| Mycophenolate Mofetil | B Transitional  CB2 MFI | 0,0242 | 0,83 | Cohen's d | 0,2634 |
| Mycophenolate Mofetil | B USm CB1 MFI | 0,4776 | -0,28 | Cohen's d | 0,8659 |
| Mycophenolate Mofetil | B USm CB2 MFI | 0,0402 | 0,77 | Cohen's d | 0,3842 |
| Mycophenolate Mofetil | eBm5 CB1 MFI | 0,5306 | -0,24 | Cohen's d | 0,8905 |
| Mycophenolate Mofetil | eBm5 CB2 MFI | 0,0908 | 0,66 | Cohen's d | 0,5622 |
| Mycophenolate Mofetil | T cellCB1 MFI | 0,5286 | -0,23 | Cohen's d | 0,8905 |
| Mycophenolate Mofetil | T cell CB2 MFI | 0,4319 | 0,29 | Cohen's d | 0,8577 |
| Mycophenolate Mofetil | T CD4 CB1 MFI | 0,6409 | -0,17 | Cohen's d | 0,9295 |
| Mycophenolate Mofetil | T CD4 CB2 MFI | 0,6917 | 0,15 | Cohen's d | 0,9336 |
| Mycophenolate Mofetil | T CD4 Effectors CB1 MFI | 0,2929 | -0,38 | Cohen's d | 0,7631 |
| Mycophenolate Mofetil | T CD4 Effectors CB2 MFI | 0,4579 | 0,29 | Cohen's d | 0,8659 |
| Mycophenolate Mofetil | T CD4 Effectors Memory CB1 MFI | 0,6125 | -0,19 | Cohen's d | 0,9252 |
| Mycophenolate Mofetil | T CD4 Effectors Memory CB2 MFI | 0,3687 | 0,34 | Cohen's d | 0,8211 |
| Mycophenolate Mofetil | T CD4 MemoryCentral CB1 MFI | 0,6397 | -0,17 | Cohen's d | 0,9295 |
| Mycophenolate Mofetil | T CD4 MemoryCentral CB2 MFI | 0,5494 | 0,23 | Cohen's d | 0,8946 |
| Mycophenolate Mofetil | T CD4 Naive CB1 MFI | 0,6202 | -0,18 | Cohen's d | 0,9278 |
| Mycophenolate Mofetil | T CD4 Naive CB2 MFI | 0,3739 | 0,34 | Cohen's d | 0,8211 |
| Mycophenolate Mofetil | T CD8 CB1 MFI | 0,3837 | -0,31 | Cohen's d | 0,8278 |
| Mycophenolate Mofetil | T CD8 CB2 MFI | 0,2278 | 0,45 | Cohen's d | 0,7475 |
| Mycophenolate Mofetil | T CD8 Effectors CB1 MFI | 0,3261 | -0,35 | Cohen's d | 0,7890 |
| Mycophenolate Mofetil | T CD8 Effectors CB2 MFI | 0,2997 | 0,38 | Cohen's d | 0,7631 |
| Mycophenolate Mofetil | T CD8 Effectors Memory CB1 MFI | 0,3016 | -0,37 | Cohen's d | 0,7653 |
| Mycophenolate Mofetil | T CD8 Effectors Memory CB2 MFI | 0,3880 | 0,31 | Cohen's d | 0,8281 |
| Mycophenolate Mofetil | T CD8 MemoryCentral CB1 MFI | 0,4073 | -0,30 | Cohen's d | 0,8417 |
| Mycophenolate Mofetil | T CD8 MemoryCentral CB2 MFI | 0,5284 | 0,22 | Cohen's d | 0,8905 |
| Mycophenolate Mofetil | T CD8 Naive CB1 MFI | 0,4144 | -0,30 | Cohen's d | 0,8417 |
| Mycophenolate Mofetil | T CD8 Naive CB2 MFI | 0,2034 | 0,49 | Cohen's d | 0,7176 |
| Mycophenolate Mofetil | T reg CB1 MFI | 0,6042 | 0,22 | Cohen's d | 0,8056 |
| Mycophenolate Mofetil | T reg CB2 MFI | 0,9181 | 0,05 | Cohen's d | 0,9653 |
| Psychiatric involvement | B cell CB1 MFI | 0,9919 | 0,00 | Cohen's d | 0,9931 |
| Psychiatric involvement | B cell CB2 MFI | 0,4672 | 0,32 | Cohen's d | 0,8659 |
| Psychiatric involvement | B eUSm CB1 MFI | 0,2060 | -0,44 | Cohen's d | 0,7176 |
| Psychiatric involvement | B eUSm CB2 MFI | 0,7694 | 0,16 | Cohen's d | 0,9490 |
| Psychiatric involvement | Bm1 CB1 MFI | 0,8910 | -0,05 | Cohen's d | 0,9624 |
| Psychiatric involvement | Bm1 CB2 MFI | 0,5312 | 0,29 | Cohen's d | 0,8905 |
| Psychiatric involvement | Bm2 CB1 MFI | 0,2655 | -0,37 | Cohen's d | 0,7572 |
| Psychiatric involvement | Bm2 CB2 MFI | 0,9338 | 0,05 | Cohen's d | 0,9717 |
| Psychiatric involvement | Bm2p CB1 MFI | 0,8678 | 0,10 | Cohen's d | 0,9624 |
| Psychiatric involvement | Bm2p CB2 MFI | 0,3402 | 0,39 | Cohen's d | 0,8036 |
| Psychiatric involvement | Bm3-4 CB1 MFI | 0,6582 | 0,24 | Cohen's d | 0,9295 |
| Psychiatric involvement | Bm3-4 CB2 MFI | 0,4629 | 0,25 | Cohen's d | 0,8659 |
| Psychiatric involvement | Bm5 CB1 MFI | 0,7691 | 0,16 | Cohen's d | 0,9490 |
| Psychiatric involvement | Bm5 CB2 MFI | 0,1800 | 0,41 | Cohen's d | 0,6976 |
| Psychiatric involvement | B Naive CB1 MFI | 0,9480 | 0,03 | Cohen's d | 0,9744 |
| Psychiatric involvement | B Naive CB2 MFI | 0,5021 | 0,29 | Cohen's d | 0,8800 |
| Psychiatric involvement | B Plasma cells CB1 MFI | 0,6196 | 0,19 | Cohen's d | 0,9278 |
| Psychiatric involvement | B Plasma cells CB2 MFI | 0,3975 | -0,22 | Cohen's d | 0,8363 |
| Psychiatric involvement | B reg CB1 MFI | 0,9520 | 0,03 | Cohen's d | 0,9773 |
| Psychiatric involvement | B reg CB2 MFI | 0,4721 | 0,25 | Cohen's d | 0,8659 |
| Psychiatric involvement | B Sm CB1 MFI | 0,6809 | 0,20 | Cohen's d | 0,9336 |
| Psychiatric involvement | B Sm CB2 MFI | 0,6270 | 0,20 | Cohen's d | 0,9278 |
| Psychiatric involvement | B Transitional  CB1 MFI | 0,9222 | 0,05 | Cohen's d | 0,9713 |
| Psychiatric involvement | B Transitional  CB2 MFI | 0,2761 | 0,40 | Cohen's d | 0,7582 |
| Psychiatric involvement | B USm CB1 MFI | 0,7829 | -0,09 | Cohen's d | 0,9490 |
| Psychiatric involvement | B USm CB2 MFI | 0,3734 | 0,42 | Cohen's d | 0,8211 |
| Psychiatric involvement | eBm5 CB1 MFI | 0,7586 | 0,16 | Cohen's d | 0,9490 |
| Psychiatric involvement | eBm5 CB2 MFI | 0,3934 | 0,35 | Cohen's d | 0,8342 |
| Psychiatric involvement | T cell CB1 MFI | 0,7949 | 0,10 | Cohen's d | 0,9490 |
| Psychiatric involvement | T cell CB2 MFI | 0,8935 | -0,08 | Cohen's d | 0,9624 |
| Psychiatric involvement | T CD4 CB1 MFI | 0,8611 | 0,08 | Cohen's d | 0,9624 |
| Psychiatric involvement | T CD4 CB2 MFI | 0,8166 | 0,16 | Cohen's d | 0,9548 |
| Psychiatric involvement | T CD4 Effectors CB1 MFI | 0,7776 | -0,08 | Cohen's d | 0,9490 |
| Psychiatric involvement | T CD4 Effectors CB2 MFI | 0,6360 | -0,23 | Cohen's d | 0,9289 |
| Psychiatric involvement | T CD4 Effectors Memory CB1 MFI | 0,7598 | 0,13 | Cohen's d | 0,9490 |
| Psychiatric involvement | T CD4 Effectors Memory CB2 MFI | 0,9347 | -0,05 | Cohen's d | 0,9717 |
| Psychiatric involvement | T CD4 MemoryCentral CB1 MFI | 0,8621 | 0,08 | Cohen's d | 0,9624 |
| Psychiatric involvement | T CD4 MemoryCentral CB2 MFI | 0,6638 | 0,32 | Cohen's d | 0,9295 |
| Psychiatric involvement | T CD4 Naive CB1 MFI | 0,8462 | 0,08 | Cohen's d | 0,9601 |
| Psychiatric involvement | T CD4 Naive CB2 MFI | 0,8548 | 0,09 | Cohen's d | 0,9624 |
| Psychiatric involvement | T CD8 CB1 MFI | 0,7309 | 0,12 | Cohen's d | 0,9399 |
| Psychiatric involvement | T CD8 CB2 MFI | 0,5420 | -0,31 | Cohen's d | 0,8926 |
| Psychiatric involvement | T CD8 Effectors CB1 MFI | 0,7085 | 0,12 | Cohen's d | 0,9372 |
| Psychiatric involvement | T CD8 Effectors CB2 MFI | 0,5770 | -0,26 | Cohen's d | 0,9116 |
| Psychiatric involvement | T CD8 Effectors Memory CB1 MFI | 0,6482 | 0,16 | Cohen's d | 0,9295 |
| Psychiatric involvement | T CD8 Effectors Memory CB2 MFI | 0,4908 | -0,34 | Cohen's d | 0,8728 |
| Psychiatric involvement | T CD8 MemoryCentral CB1 MFI | 0,8140 | 0,09 | Cohen's d | 0,9543 |
| Psychiatric involvement | T CD8 MemoryCentral CB2 MFI | 0,6888 | -0,21 | Cohen's d | 0,9336 |
| Psychiatric involvement | T CD8 Naive CB1 MFI | 0,7975 | 0,09 | Cohen's d | 0,9490 |
| Psychiatric involvement | T CD8 Naive CB2 MFI | 0,4959 | -0,32 | Cohen's d | 0,8763 |
| Psychiatric involvement | T reg CB1 MFI | 0,7967 | -0,12 | Cohen's d | 0,9293 |
| Psychiatric involvement | T reg CB2 MFI | 0,8131 | 0,17 | Cohen's d | 0,9293 |
| Pulmonary involvement | B cell CB1 MFI | 0,2010 | -0,58 | Cohen's d | 0,7176 |
| Pulmonary involvement | B cell CB2 MFI | 0,7691 | 0,13 | Cohen's d | 0,9490 |
| Pulmonary involvement | B eUSm CB1 MFI | 0,3579 | -0,44 | Cohen's d | 0,8136 |
| Pulmonary involvement | B eUSm CB2 MFI | 0,2756 | 0,40 | Cohen's d | 0,7582 |
| Pulmonary involvement | Bm1 CB1 MFI | 0,3503 | -0,54 | Cohen's d | 0,8116 |
| Pulmonary involvement | Bm1 CB2 MFI | 0,1332 | 0,65 | Cohen's d | 0,6489 |
| Pulmonary involvement | Bm2 CB1 MFI | 0,3208 | -0,47 | Cohen's d | 0,7842 |
| Pulmonary involvement | Bm2 CB2 MFI | 0,3331 | 0,33 | Cohen's d | 0,7952 |
| Pulmonary involvement | Bm2p CB1 MFI | 0,1821 | -0,58 | Cohen's d | 0,6976 |
| Pulmonary involvement | Bm2p CB2 MFI | 0,4432 | 0,31 | Cohen's d | 0,8659 |
| Pulmonary involvement | Bm3-4 CB1 MFI | 0,1552 | -0,57 | Cohen's d | 0,6570 |
| Pulmonary involvement | Bm3-4 CB2 MFI | 0,8084 | 0,11 | Cohen's d | 0,9490 |
| Pulmonary involvement | Bm5 CB1 MFI | 0,1559 | -0,48 | Cohen's d | 0,6570 |
| Pulmonary involvement | Bm5 CB2 MFI | 0,9471 | 0,03 | Cohen's d | 0,9744 |
| Pulmonary involvement | B Naive CB1 MFI | 0,2308 | -0,57 | Cohen's d | 0,7481 |
| Pulmonary involvement | B Naive CB2 MFI | 0,4997 | 0,25 | Cohen's d | 0,8800 |
| Pulmonary involvement | B Plasma cells CB1 MFI | 0,5733 | -0,34 | Cohen's d | 0,9113 |
| Pulmonary involvement | B Plasma cells CB2 MFI | 0,2703 | -0,26 | Cohen's d | 0,7572 |
| Pulmonary involvement | B reg CB1 MFI | 0,1748 | -0,57 | Cohen's d | 0,6900 |
| Pulmonary involvement | B reg CB2 MFI | 0,5295 | 0,24 | Cohen's d | 0,8905 |
| Pulmonary involvement | B Sm CB1 MFI | 0,0746 | -0,55 | Cohen's d | 0,5131 |
| Pulmonary involvement | B Sm CB2 MFI | 0,7658 | -0,10 | Cohen's d | 0,9490 |
| Pulmonary involvement | B Transitional  CB1 MFI | 0,1744 | -0,60 | Cohen's d | 0,6900 |
| Pulmonary involvement | B Transitional  CB2 MFI | 0,4803 | 0,29 | Cohen's d | 0,8659 |
| Pulmonary involvement | B USm CB1 MFI | 0,2459 | -0,54 | Cohen's d | 0,7572 |
| Pulmonary involvement | B USm CB2 MFI | 0,1477 | 0,73 | Cohen's d | 0,6570 |
| Pulmonary involvement | eBm5 CB1 MFI | 0,1326 | -0,56 | Cohen's d | 0,6489 |
| Pulmonary involvement | eBm5 CB2 MFI | 0,7977 | 0,09 | Cohen's d | 0,9490 |
| Pulmonary involvement | T cell CB1 MFI | 0,2593 | -0,30 | Cohen's d | 0,7572 |
| Pulmonary involvement | T cell CB2 MFI | 0,4427 | 0,76 | Cohen's d | 0,8659 |
| Pulmonary involvement | T CD4 CB1 MFI | 0,2051 | -0,33 | Cohen's d | 0,7176 |
| Pulmonary involvement | T CD4 CB2 MFI | 0,4944 | 0,87 | Cohen's d | 0,8755 |
| Pulmonary involvement | T CD4 Effectors CB1 MFI | 0,3686 | -0,34 | Cohen's d | 0,8211 |
| Pulmonary involvement | T CD4 Effectors CB2 MFI | 0,7916 | -0,15 | Cohen's d | 0,9490 |
| Pulmonary involvement | T CD4 Effectors Memory CB1 MFI | 0,3793 | -0,26 | Cohen's d | 0,8225 |
| Pulmonary involvement | T CD4 Effectors Memory CB2 MFI | 0,3504 | 1,07 | Cohen's d | 0,8116 |
| Pulmonary involvement | T CD4 MemoryCentral CB1 MFI | 0,2605 | -0,32 | Cohen's d | 0,7572 |
| Pulmonary involvement | T CD4 MemoryCentral CB2 MFI | 0,3599 | 1,40 | Cohen's d | 0,8136 |
| Pulmonary involvement | T CD4 Naive CB1 MFI | 0,1837 | -0,34 | Cohen's d | 0,6976 |
| Pulmonary involvement | T CD4 Naive CB2 MFI | 0,2387 | 0,80 | Cohen's d | 0,7572 |
| Pulmonary involvement | T CD8 CB1 MFI | 0,4307 | -0,28 | Cohen's d | 0,8577 |
| Pulmonary involvement | T CD8 CB2 MFI | 0,4078 | 0,44 | Cohen's d | 0,8417 |
| Pulmonary involvement | T CD8 Effectors CB1 MFI | 0,7167 | -0,16 | Cohen's d | 0,9372 |
| Pulmonary involvement | T CD8 Effectors CB2 MFI | 0,3524 | 0,61 | Cohen's d | 0,8136 |
| Pulmonary involvement | T CD8 Effectors Memory CB1 MFI | 0,8293 | -0,09 | Cohen's d | 0,9581 |
| Pulmonary involvement | T CD8 Effectors Memory CB2 MFI | 0,3124 | 0,68 | Cohen's d | 0,7765 |
| Pulmonary involvement | T CD8 MemoryCentral CB1 MFI | 0,6573 | -0,17 | Cohen's d | 0,9295 |
| Pulmonary involvement | T CD8 MemoryCentral CB2 MFI | 0,1374 | 1,02 | Cohen's d | 0,6489 |
| Pulmonary involvement | T CD8 Naive CB1 MFI | 0,3426 | -0,28 | Cohen's d | 0,8049 |
| Pulmonary involvement | T CD8 Naive CB2 MFI | 0,0789 | 0,56 | Cohen's d | 0,5256 |
| Pulmonary involvement | T reg CB1 MFI | 0,1633 | -0,66 | Cohen's d | 0,6943 |
| Pulmonary involvement | T reg CB2 MFI | 0,3705 | 1,89 | Cohen's d | 0,6943 |
| Remission status | B cell CB1 MFI | 0,0000 | -1,84 | Cohen's d | 0,0053 |
| Remission status | B cell CB2 MFI | 0,0083 | 1,00 | Cohen's d | 0,1063 |
| Remission status | B eUSm CB1 MFI | 0,0005 | -1,39 | Cohen's d | 0,0111 |
| Remission status | B eUSm CB2 MFI | 0,0146 | 0,92 | Cohen's d | 0,1750 |
| Remission status | Bm1 CB1 MFI | 0,0000 | -1,72 | Cohen's d | 0,0056 |
| Remission status | Bm1 CB2 MFI | 0,0188 | 0,88 | Cohen's d | 0,2217 |
| Remission status | Bm2 CB1 MFI | 0,0000 | -1,74 | Cohen's d | 0,0056 |
| Remission status | Bm2 CB2 MFI | 0,0029 | 1,17 | Cohen's d | 0,0450 |
| Remission status | Bm2p CB1 MFI | 0,0001 | -1,60 | Cohen's d | 0,0074 |
| Remission status | Bm2p CB2 MFI | 0,0034 | 1,13 | Cohen's d | 0,0489 |
| Remission status | Bm3-4 CB1 MFI | 0,0002 | -1,53 | Cohen's d | 0,0084 |
| Remission status | Bm3-4 CB2 MFI | 0,0505 | 0,73 | Cohen's d | 0,4209 |
| Remission status | Bm5 CB1 MFI | 0,0001 | -1,69 | Cohen's d | 0,0074 |
| Remission status | Bm5 CB2 MFI | 0,1176 | 0,57 | Cohen's d | 0,6368 |
| Remission status | B Naive CB1 MFI | 0,0000 | -1,83 | Cohen's d | 0,0053 |
| Remission status | B Naive CB2 MFI | 0,0033 | 1,14 | Cohen's d | 0,0489 |
| Remission status | B Plasma cells CB1 MFI | 0,0034 | -1,15 | Cohen's d | 0,0489 |
| Remission status | B Plasma cells CB2 MFI | 0,2249 | 0,44 | Cohen's d | 0,7451 |
| Remission status | B reg CB1 MFI | 0,0000 | -1,80 | Cohen's d | 0,0053 |
| Remission status | B reg CB2 MFI | 0,0325 | 0,79 | Cohen's d | 0,3366 |
| Remission status | B Sm CB1 MFI | 0,0003 | -1,45 | Cohen's d | 0,0101 |
| Remission status | B Sm CB2 MFI | 0,1010 | 0,61 | Cohen's d | 0,5842 |
| Remission status | B Transitional  CB1 MFI | 0,0000 | -1,69 | Cohen's d | 0,0056 |
| Remission status | B Transitional  CB2 MFI | 0,0051 | 1,07 | Cohen's d | 0,0676 |
| Remission status | B USm CB1 MFI | 0,0002 | -1,52 | Cohen's d | 0,0084 |
| Remission status | B USm CB2 MFI | 0,0536 | 0,71 | Cohen's d | 0,4383 |
| Remission status | eBm5 CB1 MFI | 0,0000 | -1,74 | Cohen's d | 0,0056 |
| Remission status | eBm5 CB2 MFI | 0,0231 | 0,85 | Cohen's d | 0,2624 |
| Remission status | T cell CB1 MFI | 0,0001 | -1,58 | Cohen's d | 0,0083 |
| Remission status | T cell CB2 MFI | 0,0476 | 0,74 | Cohen's d | 0,4158 |
| Remission status | T CD4 CB1 MFI | 0,0001 | -1,63 | Cohen's d | 0,0075 |
| Remission status | T CD4 CB2 MFI | 0,0277 | 0,83 | Cohen's d | 0,2942 |
| Remission status | T CD4 Effectors CB1 MFI | 0,0003 | -1,46 | Cohen's d | 0,0101 |
| Remission status | T CD4 Effectors CB2 MFI | 0,0010 | 1,30 | Cohen's d | 0,0170 |
| Remission status | T CD4 Effectors Memory CB1 MFI | 0,0001 | -1,60 | Cohen's d | 0,0079 |
| Remission status | T CD4 Effectors Memory CB2 MFI | 0,5631 | 0,21 | Cohen's d | 0,9039 |
| Remission status | T CD4 MemoryCentral CB1 MFI | 0,0001 | -1,67 | Cohen's d | 0,0074 |
| Remission status | T CD4 MemoryCentral CB2 MFI | 0,5988 | 0,19 | Cohen's d | 0,9252 |
| Remission status | T CD4 Naive CB1 MFI | 0,0002 | -1,55 | Cohen's d | 0,0084 |
| Remission status | T CD4 Naive CB2 MFI | 0,0043 | 1,09 | Cohen's d | 0,0580 |
| Remission status | T CD8 CB1 MFI | 0,0003 | -1,46 | Cohen's d | 0,0101 |
| Remission status | T CD8 CB2 MFI | 0,0899 | 0,62 | Cohen's d | 0,5622 |
| Remission status | T CD8 Effectors CB1 MFI | 0,0005 | -1,39 | Cohen's d | 0,0112 |
| Remission status | T CD8 Effectors CB2 MFI | 0,3191 | 0,36 | Cohen's d | 0,7839 |
| Remission status | T CD8 Effectors Memory CB1 MFI | 0,0002 | -1,48 | Cohen's d | 0,0096 |
| Remission status | T CD8 Effectors Memory CB2 MFI | 0,3223 | 0,36 | Cohen's d | 0,7850 |
| Remission status | T CD8 MemoryCentral CB1 MFI | 0,0002 | -1,53 | Cohen's d | 0,0084 |
| Remission status | T CD8 MemoryCentral CB2 MFI | 0,4440 | 0,28 | Cohen's d | 0,8659 |
| Remission status | T CD8 Naive CB1 MFI | 0,0002 | -1,49 | Cohen's d | 0,0092 |
| Remission status | T CD8 Naive CB2 MFI | 0,0240 | 0,84 | Cohen's d | 0,2634 |
| Remission status | T reg CB1 MFI | 0,0001 | -1,66 | Cohen's d | 0,0025 |
| Remission status | T reg CB2 MFI | 0,1596 | 0,51 | Cohen's d | 0,6943 |
| Renal involvement | B cell CB1 MFI | 0,3295 | -0,35 | Cohen's d | 0,7896 |
| Renal involvement | B cell CB2 MFI | 0,5721 | 0,20 | Cohen's d | 0,9113 |
| Renal involvement | B eUSm CB1 MFI | 0,7977 | -0,09 | Cohen's d | 0,9490 |
| Renal involvement | B eUSm CB2 MFI | 0,4859 | 0,25 | Cohen's d | 0,8659 |
| Renal involvement | Bm1 CB1 MFI | 0,4112 | -0,28 | Cohen's d | 0,8417 |
| Renal involvement | Bm1 CB2 MFI | 0,9911 | 0,00 | Cohen's d | 0,9931 |
| Renal involvement | Bm2 CB1 MFI | 0,5965 | -0,18 | Cohen's d | 0,9252 |
| Renal involvement | Bm2 CB2 MFI | 0,6118 | 0,18 | Cohen's d | 0,9252 |
| Renal involvement | Bm2p CB1 MFI | 0,2726 | -0,40 | Cohen's d | 0,7572 |
| Renal involvement | Bm2p CB2 MFI | 0,8841 | 0,05 | Cohen's d | 0,9624 |
| Renal involvement | Bm3-4 CB1 MFI | 0,2549 | -0,42 | Cohen's d | 0,7572 |
| Renal involvement | Bm3-4 CB2 MFI | 0,3267 | 0,32 | Cohen's d | 0,7890 |
| Renal involvement | Bm5 CB1 MFI | 0,4746 | -0,26 | Cohen's d | 0,8659 |
| Renal involvement | Bm5 CB2 MFI | 0,6059 | 0,17 | Cohen's d | 0,9252 |
| Renal involvement | B Naive CB1 MFI | 0,3479 | -0,34 | Cohen's d | 0,8112 |
| Renal involvement | B Naive CB2 MFI | 0,7603 | 0,11 | Cohen's d | 0,9490 |
| Renal involvement | B Plasma cells CB1 MFI | 0,4025 | -0,29 | Cohen's d | 0,8417 |
| Renal involvement | B Plasma cells CB2 MFI | 0,5611 | 0,20 | Cohen's d | 0,9039 |
| Renal involvement | B reg CB1 MFI | 0,3298 | -0,35 | Cohen's d | 0,7896 |
| Renal involvement | B reg CB2 MFI | 0,8381 | 0,07 | Cohen's d | 0,9587 |
| Renal involvement | B Sm CB1 MFI | 0,8340 | -0,08 | Cohen's d | 0,9587 |
| Renal involvement | B Sm CB2 MFI | 0,4586 | 0,24 | Cohen's d | 0,8659 |
| Renal involvement | B Transitional  CB1 MFI | 0,3533 | -0,33 | Cohen's d | 0,8136 |
| Renal involvement | B Transitional  CB2 MFI | 0,8771 | 0,05 | Cohen's d | 0,9624 |
| Renal involvement | B USm CB1 MFI | 0,6481 | -0,15 | Cohen's d | 0,9295 |
| Renal involvement | B USm CB2 MFI | 0,9049 | 0,04 | Cohen's d | 0,9675 |
| Renal involvement | eBm5 CB1 MFI | 0,5979 | -0,19 | Cohen's d | 0,9252 |
| Renal involvement | eBm5 CB2 MFI | 0,4749 | 0,24 | Cohen's d | 0,8659 |
| Renal involvement | T cell CB1 MFI | 0,8376 | 0,07 | Cohen's d | 0,9587 |
| Renal involvement | T cell CB2 MFI | 0,9859 | -0,01 | Cohen's d | 0,9931 |
| Renal involvement | T CD4 CB1 MFI | 0,8716 | 0,06 | Cohen's d | 0,9624 |
| Renal involvement | T CD4 CB2 MFI | 0,6776 | -0,16 | Cohen's d | 0,9336 |
| Renal involvement | T CD4 Effectors CB1 MFI | 0,9153 | -0,04 | Cohen's d | 0,9697 |
| Renal involvement | T CD4 Effectors CB2 MFI | 0,2583 | 0,39 | Cohen's d | 0,7572 |
| Renal involvement | T CD4 Effectors Memory CB1 MFI | 0,9724 | 0,01 | Cohen's d | 0,9840 |
| Renal involvement | T CD4 Effectors Memory CB2 MFI | 0,8380 | 0,07 | Cohen's d | 0,9587 |
| Renal involvement | T CD4 MemoryCentral CB1 MFI | 0,9617 | 0,02 | Cohen's d | 0,9840 |
| Renal involvement | T CD4 MemoryCentral CB2 MFI | 0,9439 | -0,03 | Cohen's d | 0,9737 |
| Renal involvement | T CD4 Naive CB1 MFI | 0,8831 | 0,05 | Cohen's d | 0,9624 |
| Renal involvement | T CD4 Naive CB2 MFI | 0,8897 | -0,05 | Cohen's d | 0,9624 |
| Renal involvement | T CD8 CB1 MFI | 0,8874 | 0,05 | Cohen's d | 0,9624 |
| Renal involvement | T CD8 CB2 MFI | 0,5440 | 0,21 | Cohen's d | 0,8926 |
| Renal involvement | T CD8 Effectors CB1 MFI | 0,8905 | 0,05 | Cohen's d | 0,9624 |
| Renal involvement | T CD8 Effectors CB2 MFI | 0,5657 | 0,20 | Cohen's d | 0,9039 |
| Renal involvement | T CD8 Effectors Memory CB1 MFI | 0,9125 | 0,04 | Cohen's d | 0,9690 |
| Renal involvement | T CD8 Effectors Memory CB2 MFI | 0,6888 | 0,14 | Cohen's d | 0,9336 |
| Renal involvement | T CD8 MemoryCentral CB1 MFI | 0,9915 | 0,00 | Cohen's d | 0,9931 |
| Renal involvement | T CD8 MemoryCentral CB2 MFI | 0,8248 | 0,08 | Cohen's d | 0,9577 |
| Renal involvement | T CD8 Naive CB1 MFI | 0,9091 | 0,04 | Cohen's d | 0,9690 |
| Renal involvement | T CD8 Naive CB2 MFI | 0,4157 | 0,28 | Cohen's d | 0,8417 |
| Renal involvement | T reg CB1 MFI | 0,3440 | 0,39 | Cohen's d | 0,6943 |
| Renal involvement | T reg CB2 MFI | 0,5162 | -0,26 | Cohen's d | 0,7509 |
| Serosal involvement | B cell CB1 MFI | 0,7127 | 0,13 | Cohen's d | 0,9372 |
| Serosal involvement | B cell CB2 MFI | 0,1903 | -0,59 | Cohen's d | 0,7158 |
| Serosal involvement | B eUSm CB1 MFI | 0,8761 | 0,05 | Cohen's d | 0,9624 |
| Serosal involvement | B eUSm CB2 MFI | 0,2937 | -0,48 | Cohen's d | 0,7631 |
| Serosal involvement | Bm1 CB1 MFI | 0,9220 | 0,04 | Cohen's d | 0,9713 |
| Serosal involvement | Bm1 CB2 MFI | 0,1320 | -0,70 | Cohen's d | 0,6489 |
| Serosal involvement | Bm2 CB1 MFI | 0,6874 | 0,15 | Cohen's d | 0,9336 |
| Serosal involvement | Bm2 CB2 MFI | 0,3456 | -0,39 | Cohen's d | 0,8092 |
| Serosal involvement | Bm2p CB1 MFI | 0,7363 | 0,12 | Cohen's d | 0,9424 |
| Serosal involvement | Bm2p CB2 MFI | 0,3674 | -0,38 | Cohen's d | 0,8211 |
| Serosal involvement | Bm3-4 CB1 MFI | 0,4435 | 0,31 | Cohen's d | 0,8659 |
| Serosal involvement | Bm3-4 CB2 MFI | 0,3857 | -0,28 | Cohen's d | 0,8278 |
| Serosal involvement | Bm5 CB1 MFI | 0,6360 | 0,24 | Cohen's d | 0,9289 |
| Serosal involvement | Bm5 CB2 MFI | 0,2484 | -0,43 | Cohen's d | 0,7572 |
| Serosal involvement | B Naive CB1 MFI | 0,8466 | 0,07 | Cohen's d | 0,9601 |
| Serosal involvement | B Naive CB2 MFI | 0,2670 | -0,49 | Cohen's d | 0,7572 |
| Serosal involvement | B Plasma cells CB1 MFI | 0,2660 | 0,44 | Cohen's d | 0,7572 |
| Serosal involvement | B Plasma cells CB2 MFI | 0,7146 | -0,14 | Cohen's d | 0,9372 |
| Serosal involvement | B reg CB1 MFI | 0,7355 | 0,12 | Cohen's d | 0,9424 |
| Serosal involvement | B reg CB2 MFI | 0,3691 | -0,37 | Cohen's d | 0,8211 |
| Serosal involvement | B Sm CB1 MFI | 0,5650 | 0,22 | Cohen's d | 0,9039 |
| Serosal involvement | B Sm CB2 MFI | 0,0844 | -0,56 | Cohen's d | 0,5444 |
| Serosal involvement | B Transitional  CB1 MFI | 0,5401 | 0,23 | Cohen's d | 0,8926 |
| Serosal involvement | B Transitional  CB2 MFI | 0,4665 | -0,31 | Cohen's d | 0,8659 |
| Serosal involvement | B USm CB1 MFI | 0,8416 | 0,07 | Cohen's d | 0,9591 |
| Serosal involvement | B USm CB2 MFI | 0,2424 | -0,51 | Cohen's d | 0,7572 |
| Serosal involvement | eBm5 CB1 MFI | 0,5833 | 0,21 | Cohen's d | 0,9164 |
| Serosal involvement | eBm5 CB2 MFI | 0,2962 | -0,37 | Cohen's d | 0,7631 |
| Serosal involvement | T cell CB1 MFI | 0,6287 | -0,27 | Cohen's d | 0,9278 |
| Serosal involvement | T cell CB2 MFI | 0,2725 | 0,49 | Cohen's d | 0,7572 |
| Serosal involvement | T CD4 CB1 MFI | 0,5296 | -0,34 | Cohen's d | 0,8905 |
| Serosal involvement | T CD4 CB2 MFI | 0,3133 | 0,37 | Cohen's d | 0,7765 |
| Serosal involvement | T CD4 Effectors CB1 MFI | 0,5645 | -0,34 | Cohen's d | 0,9039 |
| Serosal involvement | T CD4 Effectors CB2 MFI | 0,2971 | 0,64 | Cohen's d | 0,7631 |
| Serosal involvement | T CD4 Effectors Memory CB1 MFI | 0,5854 | -0,30 | Cohen's d | 0,9181 |
| Serosal involvement | T CD4 Effectors Memory CB2 MFI | 0,1241 | 0,76 | Cohen's d | 0,6462 |
| Serosal involvement | T CD4 MemoryCentral CB1 MFI | 0,5292 | -0,34 | Cohen's d | 0,8905 |
| Serosal involvement | T CD4 MemoryCentral CB2 MFI | 0,0902 | 0,67 | Cohen's d | 0,5622 |
| Serosal involvement | T CD4 Naive CB1 MFI | 0,4298 | -0,43 | Cohen's d | 0,8577 |
| Serosal involvement | T CD4 Naive CB2 MFI | 0,2627 | 0,60 | Cohen's d | 0,7572 |
| Serosal involvement | T CD8 CB1 MFI | 0,6602 | -0,26 | Cohen's d | 0,9295 |
| Serosal involvement | T CD8 CB2 MFI | 0,2156 | 0,66 | Cohen's d | 0,7301 |
| Serosal involvement | T CD8 Effectors CB1 MFI | 0,6880 | -0,23 | Cohen's d | 0,9336 |
| Serosal involvement | T CD8 Effectors CB2 MFI | 0,1199 | 0,95 | Cohen's d | 0,6448 |
| Serosal involvement | T CD8 Effectors Memory CB1 MFI | 0,6624 | -0,26 | Cohen's d | 0,9295 |
| Serosal involvement | T CD8 Effectors Memory CB2 MFI | 0,1233 | 0,96 | Cohen's d | 0,6462 |
| Serosal involvement | T CD8 MemoryCentral CB1 MFI | 0,6352 | -0,27 | Cohen's d | 0,9289 |
| Serosal involvement | T CD8 MemoryCentral CB2 MFI | 0,2864 | 0,54 | Cohen's d | 0,7631 |
| Serosal involvement | T CD8 Naive CB1 MFI | 0,5654 | -0,35 | Cohen's d | 0,9039 |
| Serosal involvement | T CD8 Naive CB2 MFI | 0,2441 | 0,71 | Cohen's d | 0,7572 |
| Serosal involvement | T reg CB1 MFI | 0,7368 | -0,15 | Cohen's d | 0,9069 |
| Serosal involvement | T reg CB2 MFI | 0,2612 | 0,53 | Cohen's d | 0,6943 |
| Skin involvement | B cell CB1 MFI | 0,2061 | -0,46 | Cohen's d | 0,7176 |
| Skin involvement | B cell CB2 MFI | 0,7864 | 0,10 | Cohen's d | 0,9490 |
| Skin involvement | B eUSm CB1 MFI | 0,1019 | -0,60 | Cohen's d | 0,5852 |
| Skin involvement | B eUSm CB2 MFI | 0,8216 | 0,08 | Cohen's d | 0,9567 |
| Skin involvement | Bm1 CB1 MFI | 0,1351 | -0,54 | Cohen's d | 0,6489 |
| Skin involvement | Bm1 CB2 MFI | 0,3722 | 0,32 | Cohen's d | 0,8211 |
| Skin involvement | Bm2 CB1 MFI | 0,0889 | -0,62 | Cohen's d | 0,5622 |
| Skin involvement | Bm2 CB2 MFI | 0,9479 | -0,02 | Cohen's d | 0,9744 |
| Skin involvement | Bm2p CB1 MFI | 0,2605 | -0,41 | Cohen's d | 0,7572 |
| Skin involvement | Bm2p CB2 MFI | 0,6961 | 0,14 | Cohen's d | 0,9336 |
| Skin involvement | Bm3-4 CB1 MFI | 0,1328 | -0,55 | Cohen's d | 0,6489 |
| Skin involvement | Bm3-4 CB2 MFI | 0,7972 | 0,09 | Cohen's d | 0,9490 |
| Skin involvement | Bm5 CB1 MFI | 0,2661 | -0,40 | Cohen's d | 0,7572 |
| Skin involvement | Bm5 CB2 MFI | 0,9685 | -0,01 | Cohen's d | 0,9840 |
| Skin involvement | B Naive CB1 MFI | 0,2255 | -0,44 | Cohen's d | 0,7451 |
| Skin involvement | B Naive CB2 MFI | 0,8834 | 0,05 | Cohen's d | 0,9624 |
| Skin involvement | B Plasma cells CB1 MFI | 0,2999 | -0,37 | Cohen's d | 0,7631 |
| Skin involvement | B Plasma cells CB2 MFI | 0,7760 | 0,10 | Cohen's d | 0,9490 |
| Skin involvement | B reg CB1 MFI | 0,2967 | -0,38 | Cohen's d | 0,7631 |
| Skin involvement | B reg CB2 MFI | 0,3936 | 0,31 | Cohen's d | 0,8342 |
| Skin involvement | B Sm CB1 MFI | 0,3091 | -0,37 | Cohen's d | 0,7720 |
| Skin involvement | B Sm CB2 MFI | 0,7723 | 0,10 | Cohen's d | 0,9490 |
| Skin involvement | B Transitional  CB1 MFI | 0,2094 | -0,45 | Cohen's d | 0,7177 |
| Skin involvement | B Transitional  CB2 MFI | 0,6953 | 0,14 | Cohen's d | 0,9336 |
| Skin involvement | B USm CB1 MFI | 0,0756 | -0,65 | Cohen's d | 0,5131 |
| Skin involvement | B USm CB2 MFI | 0,5171 | 0,23 | Cohen's d | 0,8843 |
| Skin involvement | eBm5 CB1 MFI | 0,3072 | -0,37 | Cohen's d | 0,7702 |
| Skin involvement | eBm5 CB2 MFI | 0,8418 | -0,07 | Cohen's d | 0,9591 |
| Skin involvement | T cell CB1 MFI | 0,9348 | -0,03 | Cohen's d | 0,9717 |
| Skin involvement | T cell CB2 MFI | 0,5757 | -0,20 | Cohen's d | 0,9113 |
| Skin involvement | T CD4 CB1 MFI | 0,8821 | -0,05 | Cohen's d | 0,9624 |
| Skin involvement | T CD4 CB2 MFI | 0,9357 | -0,03 | Cohen's d | 0,9717 |
| Skin involvement | T CD4 Effectors CB1 MFI | 0,8248 | -0,08 | Cohen's d | 0,9577 |
| Skin involvement | T CD4 Effectors CB2 MFI | 0,8817 | 0,05 | Cohen's d | 0,9624 |
| Skin involvement | T CD4 Effectors Memory CB1 MFI | 0,9366 | 0,03 | Cohen's d | 0,9717 |
| Skin involvement | T CD4 Effectors Memory CB2 MFI | 0,6537 | 0,16 | Cohen's d | 0,9295 |
| Skin involvement | T CD4 MemoryCentral CB1 MFI | 0,9260 | -0,03 | Cohen's d | 0,9717 |
| Skin involvement | T CD4 MemoryCentral CB2 MFI | 0,5615 | 0,21 | Cohen's d | 0,9039 |
| Skin involvement | T CD4 Naive CB1 MFI | 0,8299 | -0,08 | Cohen's d | 0,9581 |
| Skin involvement | T CD4 Naive CB2 MFI | 0,8291 | 0,08 | Cohen's d | 0,9581 |
| Skin involvement | T CD8 CB1 MFI | 0,8946 | 0,05 | Cohen's d | 0,9624 |
| Skin involvement | T CD8 CB2 MFI | 0,2853 | -0,39 | Cohen's d | 0,7631 |
| Skin involvement | T CD8 Effectors CB1 MFI | 0,7165 | 0,13 | Cohen's d | 0,9372 |
| Skin involvement | T CD8 Effectors CB2 MFI | 0,9695 | 0,01 | Cohen's d | 0,9840 |
| Skin involvement | T CD8 Effectors Memory CB1 MFI | 0,6091 | 0,18 | Cohen's d | 0,9252 |
| Skin involvement | T CD8 Effectors Memory CB2 MFI | 0,6606 | 0,16 | Cohen's d | 0,9295 |
| Skin involvement | T CD8 MemoryCentral CB1 MFI | 0,7652 | 0,11 | Cohen's d | 0,9490 |
| Skin involvement | T CD8 MemoryCentral CB2 MFI | 0,8049 | 0,09 | Cohen's d | 0,9490 |
| Skin involvement | T CD8 Naive CB1 MFI | 0,9385 | -0,03 | Cohen's d | 0,9717 |
| Skin involvement | T CD8 Naive CB2 MFI | 0,3363 | -0,35 | Cohen's d | 0,8007 |
| Skin involvement | T reg CB1 MFI | 0,4299 | -0,32 | Cohen's d | 0,6943 |
| Skin involvement | T reg CB2 MFI | 0,4339 | 0,32 | Cohen's d | 0,6943 |
| Steroids | B cell CB1 MFI | 0,4859 | -0,24 | Cohen's d | 0,8659 |
| Steroids | B cell CB2 MFI | 0,1720 | 0,45 | Cohen's d | 0,6900 |
| Steroids | B eUSm CB1 MFI | 0,1096 | -0,55 | Cohen's d | 0,6130 |
| Steroids | B eUSm CB2 MFI | 0,5076 | 0,20 | Cohen's d | 0,8823 |
| Steroids | Bm1 CB1 MFI | 0,3404 | -0,31 | Cohen's d | 0,8036 |
| Steroids | Bm1 CB2 MFI | 0,5333 | 0,20 | Cohen's d | 0,8905 |
| Steroids | Bm2 CB1 MFI | 0,2454 | -0,40 | Cohen's d | 0,7572 |
| Steroids | Bm2 CB2 MFI | 0,6319 | 0,15 | Cohen's d | 0,9289 |
| Steroids | Bm2p CB1 MFI | 0,4122 | -0,25 | Cohen's d | 0,8417 |
| Steroids | Bm2p CB2 MFI | 0,4498 | 0,24 | Cohen's d | 0,8659 |
| Steroids | Bm3-4 CB1 MFI | 0,8723 | 0,05 | Cohen's d | 0,9624 |
| Steroids | Bm3-4 CB2 MFI | 0,2011 | 0,35 | Cohen's d | 0,7176 |
| Steroids | Bm5 CB1 MFI | 0,9588 | -0,02 | Cohen's d | 0,9831 |
| Steroids | Bm5 CB2 MFI | 0,0515 | 0,63 | Cohen's d | 0,4250 |
| Steroids | B Naive CB1 MFI | 0,4127 | -0,26 | Cohen's d | 0,8417 |
| Steroids | B Naive CB2 MFI | 0,4753 | 0,24 | Cohen's d | 0,8659 |
| Steroids | B Plasma cells CB1 MFI | 0,2975 | -0,37 | Cohen's d | 0,7631 |
| Steroids | B Plasma cells CB2 MFI | 0,6267 | 0,17 | Cohen's d | 0,9278 |
| Steroids | B reg CB1 MFI | 0,3887 | -0,26 | Cohen's d | 0,8281 |
| Steroids | B reg CB2 MFI | 0,3951 | 0,28 | Cohen's d | 0,8351 |
| Steroids | B Sm CB1 MFI | 0,6618 | -0,16 | Cohen's d | 0,9295 |
| Steroids | B Sm CB2 MFI | 0,1518 | 0,39 | Cohen's d | 0,6570 |
| Steroids | B Transitional  CB1 MFI | 0,3769 | -0,27 | Cohen's d | 0,8217 |
| Steroids | B Transitional  CB2 MFI | 0,4466 | 0,25 | Cohen's d | 0,8659 |
| Steroids | B USm CB1 MFI | 0,5418 | -0,19 | Cohen's d | 0,8926 |
| Steroids | B USm CB2 MFI | 0,3245 | 0,36 | Cohen's d | 0,7881 |
| Steroids | eBm5 CB1 MFI | 0,6451 | -0,16 | Cohen's d | 0,9295 |
| Steroids | eBm5 CB2 MFI | 0,1373 | 0,44 | Cohen's d | 0,6489 |
| Steroids | T cell CB1 MFI | 0,9290 | -0,03 | Cohen's d | 0,9717 |
| Steroids | T cell CB2 MFI | 0,2621 | 0,39 | Cohen's d | 0,7572 |
| Steroids | T CD4 CB1 MFI | 0,9712 | -0,01 | Cohen's d | 0,9840 |
| Steroids | T CD4 CB2 MFI | 0,2402 | 0,39 | Cohen's d | 0,7572 |
| Steroids | T CD4 Effectors CB1 MFI | 0,8905 | -0,06 | Cohen's d | 0,9624 |
| Steroids | T CD4 Effectors CB2 MFI | 0,7154 | -0,20 | Cohen's d | 0,9372 |
| Steroids | T CD4 Effectors Memory CB1 MFI | 0,9172 | 0,04 | Cohen's d | 0,9697 |
| Steroids | T CD4 Effectors Memory CB2 MFI | 0,4489 | 0,22 | Cohen's d | 0,8659 |
| Steroids | T CD4 MemoryCentral CB1 MFI | 0,9129 | -0,04 | Cohen's d | 0,9690 |
| Steroids | T CD4 MemoryCentral CB2 MFI | 0,3780 | 0,28 | Cohen's d | 0,8217 |
| Steroids | T CD4 Naive CB1 MFI | 0,9715 | 0,01 | Cohen's d | 0,9840 |
| Steroids | T CD4 Naive CB2 MFI | 0,8695 | 0,08 | Cohen's d | 0,9624 |
| Steroids | T CD8 CB1 MFI | 0,7743 | -0,10 | Cohen's d | 0,9490 |
| Steroids | T CD8 CB2 MFI | 0,5002 | 0,25 | Cohen's d | 0,8800 |
| Steroids | T CD8 Effectors CB1 MFI | 0,6529 | -0,15 | Cohen's d | 0,9295 |
| Steroids | T CD8 Effectors CB2 MFI | 0,4301 | 0,30 | Cohen's d | 0,8577 |
| Steroids | T CD8 Effectors Memory CB1 MFI | 0,8307 | -0,07 | Cohen's d | 0,9581 |
| Steroids | T CD8 Effectors Memory CB2 MFI | 0,6571 | 0,16 | Cohen's d | 0,9295 |
| Steroids | T CD8 MemoryCentral CB1 MFI | 0,8080 | -0,08 | Cohen's d | 0,9490 |
| Steroids | T CD8 MemoryCentral CB2 MFI | 0,2847 | 0,34 | Cohen's d | 0,7631 |
| Steroids | T CD8 Naive CB1 MFI | 0,9020 | -0,04 | Cohen's d | 0,9675 |
| Steroids | T CD8 Naive CB2 MFI | 0,4823 | 0,26 | Cohen's d | 0,8659 |
| Steroids | T reg CB1 MFI | 0,5919 | 0,18 | Cohen's d | 0,8056 |
| Steroids | T reg CB2 MFI | 0,9181 | 0,04 | Cohen's d | 0,9653 |

1. **Correlation** **between laboratory and numeric clinical variables**

| **Clinical variable** | **Laboratory variable** | **Correlation Coefficient** | **p value** | **FDR** |
| --- | --- | --- | --- | --- |
| Age | B cell CB1 MFI | 0,3077 | 0,0866 | 0,2746 |
| Age | B cell CB2 MFI | -0,2441 | 0,1782 | 0,3786 |
| Age | B eUSm CB1 MFI | 0,2114 | 0,2455 | 0,4587 |
| Age | B eUSm CB2 MFI | -0,2704 | 0,1344 | 0,3324 |
| Age | Bm1 CB1 MFI | 0,3059 | 0,0886 | 0,2789 |
| Age | Bm1 CB2 MFI | -0,2912 | 0,1059 | 0,3065 |
| Age | Bm2 CB1 MFI | 0,2631 | 0,1456 | 0,3488 |
| Age | Bm2 CB2 MFI | -0,3802 | 0,0318 | 0,1702 |
| Age | Bm2p CB1 MFI | 0,2712 | 0,1333 | 0,3324 |
| Age | Bm2p CB2 MFI | -0,3261 | 0,0685 | 0,2544 |
| Age | Bm3-4 CB1 MFI | 0,2793 | 0,1216 | 0,3249 |
| Age | Bm3-4 CB2 MFI | -0,2660 | 0,1411 | 0,3433 |
| Age | Bm5 CB1 MFI | 0,3551 | 0,0461 | 0,2061 |
| Age | Bm5 CB2 MFI | -0,2110 | 0,2463 | 0,4587 |
| Age | B Naive CB1 MFI | 0,2917 | 0,1052 | 0,3065 |
| Age | B Naive CB2 MFI | -0,3925 | 0,0263 | 0,1621 |
| Age | B Plasma Cells CB1 MFI | 0,1958 | 0,2827 | 0,4932 |
| Age | B Plasma Cells CB2 MFI | -0,0840 | 0,6474 | 0,8138 |
| Age | B reg CB1 MFI | 0,2683 | 0,1376 | 0,3366 |
| Age | B reg CB2 MFI | -0,3046 | 0,0901 | 0,2812 |
| Age | B Sm CB1 MFI | 0,1908 | 0,2954 | 0,5036 |
| Age | B Sm CB2 MFI | -0,1275 | 0,4866 | 0,6843 |
| Age | BTransitional CB1 MFI | 0,2513 | 0,1653 | 0,3669 |
| Age | BTransitional CB2 MFI | -0,3250 | 0,0695 | 0,2544 |
| Age | B USm CB1 MFI | 0,3201 | 0,0741 | 0,2586 |
| Age | B USm CB2 MFI | -0,2160 | 0,2351 | 0,4541 |
| Age | eBm5 CB1 MFI | 0,2576 | 0,1546 | 0,3567 |
| Age | eBm5 CB2 MFI | -0,2461 | 0,1746 | 0,3759 |
| Age | T cell CB1 MFI | 0,0900 | 0,6242 | 0,8002 |
| Age | T cell CB2 MFI | 0,0433 | 0,8139 | 0,8915 |
| Age | T CD4 CB1 MFI | 0,0791 | 0,6670 | 0,8177 |
| Age | T CD4 CB2 MFI | -0,0521 | 0,7769 | 0,8697 |
| Age | T CD4 Effectors CB1 MFI | 0,0820 | 0,6554 | 0,8170 |
| Age | T CD4 Effectors CB2 MFI | -0,1794 | 0,3259 | 0,5333 |
| Age | T CD4 EffectorsMemory CB1 MFI | 0,0728 | 0,6923 | 0,8307 |
| Age | T CD4 EffectorsMemory CB2 MFI | -0,0563 | 0,7594 | 0,8608 |
| Age | T CD4 MemoryCentral CB1 MFI | 0,0844 | 0,6460 | 0,8138 |
| Age | T CD4 MemoryCentral CB2 MFI | -0,0200 | 0,9135 | 0,9394 |
| Age | T CD4 Naive CB1 MFI | 0,0965 | 0,5992 | 0,7880 |
| Age | T CD4 Naive CB2 MFI | 0,0290 | 0,8748 | 0,9198 |
| Age | T CD8 CB1 MFI | 0,1279 | 0,4854 | 0,6843 |
| Age | T CD8 CB2 MFI | 0,1734 | 0,3425 | 0,5504 |
| Age | T CD8 Effectors CB1 MFI | 0,0323 | 0,8607 | 0,9131 |
| Age | T CD8 Effectors CB2 MFI | 0,0657 | 0,7209 | 0,8450 |
| Age | T CD8 EffectorsMemory CB1 MFI | 0,0630 | 0,7321 | 0,8450 |
| Age | T CD8 EffectorsMemory CB2 MFI | 0,0800 | 0,6633 | 0,8177 |
| Age | T CD8 MemoryCentral CB1 MFI | 0,1110 | 0,5452 | 0,7503 |
| Age | T CD8 MemoryCentral CB2 MFI | 0,1458 | 0,4259 | 0,6321 |
| Age | T CD8 Naive CB1 MFI | 0,0820 | 0,6554 | 0,8170 |
| Age | T CD8 Naive CB2 MFI | 0,0066 | 0,9714 | 0,9823 |
| Age | T reg CB1 MFI | 0,0621 | 0,7682 | 0,9218 |
| Age | T reg CB2 MFI | 0,1319 | 0,5298 | 0,7947 |
| C3 | B cell CB1 MFI | 0,3193 | 0,0748 | 0,2586 |
| C3 | B cell CB2 MFI | -0,3185 | 0,0756 | 0,2586 |
| C3 | B eUSm CB1 MFI | 0,4642 | 0,0074 | 0,0906 |
| C3 | B eUSm CB2 MFI | -0,3115 | 0,0827 | 0,2688 |
| C3 | Bm1 CB1 MFI | 0,3384 | 0,0582 | 0,2401 |
| C3 | Bm1 CB2 MFI | -0,2865 | 0,1119 | 0,3127 |
| C3 | Bm2 CB1 MFI | 0,4227 | 0,0159 | 0,1265 |
| C3 | Bm2 CB2 MFI | -0,3335 | 0,0622 | 0,2446 |
| C3 | Bm2p CB1 MFI | 0,3102 | 0,0840 | 0,2691 |
| C3 | Bm2p CB2 MFI | -0,4185 | 0,0171 | 0,1282 |
| C3 | Bm3-4 CB1 MFI | 0,2898 | 0,1076 | 0,3065 |
| C3 | Bm3-4 CB2 MFI | -0,2781 | 0,1233 | 0,3249 |
| C3 | Bm5 CB1 MFI | 0,3217 | 0,0726 | 0,2583 |
| C3 | Bm5 CB2 MFI | -0,3298 | 0,0653 | 0,2511 |
| C3 | B Naive CB1 MFI | 0,3351 | 0,0608 | 0,2423 |
| C3 | B Naive CB2 MFI | -0,3810 | 0,0315 | 0,1702 |
| C3 | B Plasma Cells CB1 MFI | 0,2236 | 0,2186 | 0,4296 |
| C3 | B Plasma Cells CB2 MFI | -0,1773 | 0,3317 | 0,5370 |
| C3 | B reg CB1 MFI | 0,3538 | 0,0470 | 0,2061 |
| C3 | B reg CB2 MFI | -0,2869 | 0,1113 | 0,3127 |
| C3 | B Sm CB1 MFI | 0,1936 | 0,2884 | 0,4973 |
| C3 | B Sm CB2 MFI | -0,1450 | 0,4284 | 0,6321 |
| C3 | BTransitional CB1 MFI | 0,3183 | 0,0759 | 0,2586 |
| C3 | BTransitional CB2 MFI | -0,3599 | 0,0431 | 0,2019 |
| C3 | B USm CB1 MFI | 0,3128 | 0,0813 | 0,2688 |
| C3 | B USm CB2 MFI | -0,3362 | 0,0599 | 0,2423 |
| C3 | eBm5 CB1 MFI | 0,2730 | 0,1307 | 0,3324 |
| C3 | eBm5 CB2 MFI | -0,3456 | 0,0527 | 0,2239 |
| C3 | T cell CB1 MFI | 0,2869 | 0,1113 | 0,3127 |
| C3 | T cell CB2 MFI | -0,3243 | 0,0702 | 0,2547 |
| C3 | T CD4 CB1 MFI | 0,3014 | 0,0937 | 0,2868 |
| C3 | T CD4 CB2 MFI | -0,4074 | 0,0207 | 0,1431 |
| C3 | T CD4 Effectors CB1 MFI | 0,2565 | 0,1565 | 0,3567 |
| C3 | T CD4 Effectors CB2 MFI | -0,3529 | 0,0476 | 0,2061 |
| C3 | T CD4 EffectorsMemory CB1 MFI | 0,2941 | 0,1023 | 0,3029 |
| C3 | T CD4 EffectorsMemory CB2 MFI | -0,0378 | 0,8374 | 0,9027 |
| C3 | T CD4 MemoryCentral CB1 MFI | 0,3214 | 0,0729 | 0,2583 |
| C3 | T CD4 MemoryCentral CB2 MFI | -0,1542 | 0,3995 | 0,6042 |
| C3 | T CD4 Naive CB1 MFI | 0,2780 | 0,1235 | 0,3249 |
| C3 | T CD4 Naive CB2 MFI | -0,3547 | 0,0464 | 0,2061 |
| C3 | T CD8 CB1 MFI | 0,2577 | 0,1544 | 0,3567 |
| C3 | T CD8 CB2 MFI | -0,1159 | 0,5277 | 0,7284 |
| C3 | T CD8 Effectors CB1 MFI | 0,2097 | 0,2494 | 0,4587 |
| C3 | T CD8 Effectors CB2 MFI | 0,0356 | 0,8468 | 0,9029 |
| C3 | T CD8 EffectorsMemory CB1 MFI | 0,2743 | 0,1287 | 0,3324 |
| C3 | T CD8 EffectorsMemory CB2 MFI | -0,0070 | 0,9698 | 0,9823 |
| C3 | T CD8 MemoryCentral CB1 MFI | 0,2692 | 0,1362 | 0,3349 |
| C3 | T CD8 MemoryCentral CB2 MFI | -0,0875 | 0,6341 | 0,8038 |
| C3 | T CD8 Naive CB1 MFI | 0,2717 | 0,1325 | 0,3324 |
| C3 | T CD8 Naive CB2 MFI | -0,2440 | 0,1784 | 0,3786 |
| C3 | T reg CB1 MFI | 0,3269 | 0,1107 | 0,3321 |
| C3 | T reg CB2 MFI | -0,3889 | 0,0547 | 0,2460 |
| C4 | B cell CB1 MFI | 0,2587 | 0,1529 | 0,3567 |
| C4 | B cell CB2 MFI | -0,2061 | 0,2577 | 0,4639 |
| C4 | B eUSm CB1 MFI | 0,4573 | 0,0085 | 0,1006 |
| C4 | B eUSm CB2 MFI | -0,2348 | 0,1957 | 0,4086 |
| C4 | Bm1 CB1 MFI | 0,2559 | 0,1574 | 0,3567 |
| C4 | Bm1 CB2 MFI | -0,2244 | 0,2170 | 0,4296 |
| C4 | Bm2 CB1 MFI | 0,3898 | 0,0274 | 0,1628 |
| C4 | Bm2 CB2 MFI | -0,2299 | 0,2057 | 0,4177 |
| C4 | Bm2p CB1 MFI | 0,2768 | 0,1252 | 0,3275 |
| C4 | Bm2p CB2 MFI | -0,2816 | 0,1184 | 0,3249 |
| C4 | Bm3-4 CB1 MFI | 0,2225 | 0,2209 | 0,4322 |
| C4 | Bm3-4 CB2 MFI | -0,1675 | 0,3595 | 0,5630 |
| C4 | Bm5 CB1 MFI | 0,2414 | 0,1831 | 0,3869 |
| C4 | Bm5 CB2 MFI | -0,2312 | 0,2031 | 0,4172 |
| C4 | B Naive CB1 MFI | 0,2792 | 0,1217 | 0,3249 |
| C4 | B Naive CB2 MFI | -0,3000 | 0,0953 | 0,2878 |
| C4 | B Plasma Cells CB1 MFI | 0,1614 | 0,3776 | 0,5799 |
| C4 | B Plasma Cells CB2 MFI | -0,1038 | 0,5717 | 0,7719 |
| C4 | B reg CB1 MFI | 0,3111 | 0,0830 | 0,2688 |
| C4 | B reg CB2 MFI | -0,1660 | 0,3638 | 0,5645 |
| C4 | B Sm CB1 MFI | 0,1952 | 0,2844 | 0,4941 |
| C4 | B Sm CB2 MFI | -0,0424 | 0,8179 | 0,8915 |
| C4 | BTransitional CB1 MFI | 0,2903 | 0,1070 | 0,3065 |
| C4 | BTransitional CB2 MFI | -0,2462 | 0,1744 | 0,3759 |
| C4 | B USm CB1 MFI | 0,2631 | 0,1457 | 0,3488 |
| C4 | B USm CB2 MFI | -0,2497 | 0,1682 | 0,3687 |
| C4 | eBm5 CB1 MFI | 0,2126 | 0,2427 | 0,4587 |
| C4 | eBm5 CB2 MFI | -0,2493 | 0,1688 | 0,3687 |
| C4 | T cell CB1 MFI | 0,1420 | 0,4382 | 0,6402 |
| C4 | T cell CB2 MFI | -0,2948 | 0,1014 | 0,3023 |
| C4 | T CD4 CB1 MFI | 0,1513 | 0,4083 | 0,6104 |
| C4 | T CD4 CB2 MFI | -0,3535 | 0,0472 | 0,2061 |
| C4 | T CD4 Effectors CB1 MFI | 0,1003 | 0,5848 | 0,7824 |
| C4 | T CD4 Effectors CB2 MFI | -0,3528 | 0,0476 | 0,2061 |
| C4 | T CD4 EffectorsMemory CB1 MFI | 0,1711 | 0,3492 | 0,5534 |
| C4 | T CD4 EffectorsMemory CB2 MFI | -0,0593 | 0,7473 | 0,8536 |
| C4 | T CD4 MemoryCentral CB1 MFI | 0,1706 | 0,3505 | 0,5534 |
| C4 | T CD4 MemoryCentral CB2 MFI | -0,1299 | 0,4786 | 0,6773 |
| C4 | T CD4 Naive CB1 MFI | 0,1270 | 0,4886 | 0,6850 |
| C4 | T CD4 Naive CB2 MFI | -0,2726 | 0,1312 | 0,3324 |
| C4 | T CD8 CB1 MFI | 0,1260 | 0,4919 | 0,6874 |
| C4 | T CD8 CB2 MFI | -0,0679 | 0,7120 | 0,8450 |
| C4 | T CD8 Effectors CB1 MFI | 0,1043 | 0,5699 | 0,7719 |
| C4 | T CD8 Effectors CB2 MFI | 0,0456 | 0,8043 | 0,8915 |
| C4 | T CD8 EffectorsMemory CB1 MFI | 0,1626 | 0,3740 | 0,5784 |
| C4 | T CD8 EffectorsMemory CB2 MFI | 0,0418 | 0,8202 | 0,8915 |
| C4 | T CD8 MemoryCentral CB1 MFI | 0,1337 | 0,4658 | 0,6655 |
| C4 | T CD8 MemoryCentral CB2 MFI | 0,0076 | 0,9670 | 0,9823 |
| C4 | T CD8 Naive CB1 MFI | 0,1092 | 0,5520 | 0,7528 |
| C4 | T CD8 Naive CB2 MFI | -0,2093 | 0,2502 | 0,4587 |
| C4 | T reg CB1 MFI | 0,2432 | 0,2415 | 0,4654 |
| C4 | T reg CB2 MFI | -0,4310 | 0,0315 | 0,2242 |
| Hemoglobin | B cell CB1 MFI | 0,4222 | 0,0161 | 0,1265 |
| Hemoglobin | B cell CB2 MFI | -0,0369 | 0,8412 | 0,9029 |
| Hemoglobin | B eUSm CB1 MFI | 0,5042 | 0,0033 | 0,0523 |
| Hemoglobin | B eUSm CB2 MFI | -0,0378 | 0,8373 | 0,9027 |
| Hemoglobin | Bm1 CB1 MFI | 0,4505 | 0,0097 | 0,1018 |
| Hemoglobin | Bm1 CB2 MFI | -0,1049 | 0,5677 | 0,7718 |
| Hemoglobin | Bm2 CB1 MFI | 0,5108 | 0,0028 | 0,0487 |
| Hemoglobin | Bm2 CB2 MFI | -0,1099 | 0,5494 | 0,7528 |
| Hemoglobin | Bm2p CB1 MFI | 0,3578 | 0,0444 | 0,2042 |
| Hemoglobin | Bm2p CB2 MFI | -0,2599 | 0,1508 | 0,3566 |
| Hemoglobin | Bm3-4 CB1 MFI | 0,3520 | 0,0482 | 0,2066 |
| Hemoglobin | Bm3-4 CB2 MFI | -0,0639 | 0,7281 | 0,8450 |
| Hemoglobin | Bm5 CB1 MFI | 0,4420 | 0,0113 | 0,1131 |
| Hemoglobin | Bm5 CB2 MFI | -0,0629 | 0,7323 | 0,8450 |
| Hemoglobin | B Naive CB1 MFI | 0,4232 | 0,0158 | 0,1265 |
| Hemoglobin | B Naive CB2 MFI | -0,1363 | 0,4570 | 0,6591 |
| Hemoglobin | B Plasma Cells CB1 MFI | 0,2289 | 0,2075 | 0,4177 |
| Hemoglobin | B Plasma Cells CB2 MFI | 0,0211 | 0,9088 | 0,9394 |
| Hemoglobin | B reg CB1 MFI | 0,3905 | 0,0271 | 0,1628 |
| Hemoglobin | B reg CB2 MFI | -0,1803 | 0,3234 | 0,5311 |
| Hemoglobin | B Sm CB1 MFI | 0,3259 | 0,0687 | 0,2544 |
| Hemoglobin | B Sm CB2 MFI | -0,0238 | 0,8973 | 0,9347 |
| Hemoglobin | BTransitional CB1 MFI | 0,3797 | 0,0321 | 0,1702 |
| Hemoglobin | BTransitional CB2 MFI | -0,2507 | 0,1663 | 0,3669 |
| Hemoglobin | B USm CB1 MFI | 0,5115 | 0,0028 | 0,0487 |
| Hemoglobin | B USm CB2 MFI | -0,1189 | 0,5170 | 0,7159 |
| Hemoglobin | eBm5 CB1 MFI | 0,4054 | 0,0214 | 0,1435 |
| Hemoglobin | eBm5 CB2 MFI | -0,0880 | 0,6318 | 0,8038 |
| Hemoglobin | T cell CB1 MFI | 0,2860 | 0,1126 | 0,3127 |
| Hemoglobin | T cell CB2 MFI | -0,0503 | 0,7847 | 0,8742 |
| Hemoglobin | T CD4 CB1 MFI | 0,2792 | 0,1218 | 0,3249 |
| Hemoglobin | T CD4 CB2 MFI | -0,1716 | 0,3476 | 0,5534 |
| Hemoglobin | T CD4 Effectors CB1 MFI | 0,2252 | 0,2152 | 0,4296 |
| Hemoglobin | T CD4 Effectors CB2 MFI | -0,2733 | 0,1301 | 0,3324 |
| Hemoglobin | T CD4 EffectorsMemory CB1 MFI | 0,2610 | 0,1490 | 0,3548 |
| Hemoglobin | T CD4 EffectorsMemory CB2 MFI | 0,1787 | 0,3279 | 0,5347 |
| Hemoglobin | T CD4 MemoryCentral CB1 MFI | 0,3041 | 0,0906 | 0,2812 |
| Hemoglobin | T CD4 MemoryCentral CB2 MFI | 0,0846 | 0,6454 | 0,8138 |
| Hemoglobin | T CD4 Naive CB1 MFI | 0,2463 | 0,1742 | 0,3759 |
| Hemoglobin | T CD4 Naive CB2 MFI | -0,2008 | 0,2704 | 0,4790 |
| Hemoglobin | T CD8 CB1 MFI | 0,2445 | 0,1775 | 0,3786 |
| Hemoglobin | T CD8 CB2 MFI | 0,0912 | 0,6197 | 0,7987 |
| Hemoglobin | T CD8 Effectors CB1 MFI | 0,1717 | 0,3474 | 0,5534 |
| Hemoglobin | T CD8 Effectors CB2 MFI | 0,1352 | 0,4607 | 0,6602 |
| Hemoglobin | T CD8 EffectorsMemory CB1 MFI | 0,2010 | 0,2701 | 0,4790 |
| Hemoglobin | T CD8 EffectorsMemory CB2 MFI | 0,0947 | 0,6063 | 0,7889 |
| Hemoglobin | T CD8 MemoryCentral CB1 MFI | 0,2300 | 0,2053 | 0,4177 |
| Hemoglobin | T CD8 MemoryCentral CB2 MFI | 0,1877 | 0,3037 | 0,5112 |
| Hemoglobin | T CD8 Naive CB1 MFI | 0,2561 | 0,1572 | 0,3567 |
| Hemoglobin | T CD8 Naive CB2 MFI | -0,0556 | 0,7626 | 0,8622 |
| Hemoglobin | T reg CB1 MFI | 0,3065 | 0,1362 | 0,3501 |
| Hemoglobin | T reg CB2 MFI | -0,0474 | 0,8221 | 0,9249 |
| Leukocytes | B cell CB1 MFI | 0,2090 | 0,2511 | 0,4587 |
| Leukocytes | B cell CB2 MFI | 0,0413 | 0,8223 | 0,8916 |
| Leukocytes | B eUSm CB1 MFI | 0,3331 | 0,0625 | 0,2446 |
| Leukocytes | B eUSm CB2 MFI | 0,0022 | 0,9905 | 0,9927 |
| Leukocytes | Bm1 CB1 MFI | 0,1767 | 0,3333 | 0,5376 |
| Leukocytes | Bm1 CB2 MFI | -0,0973 | 0,5961 | 0,7867 |
| Leukocytes | Bm2 CB1 MFI | 0,2788 | 0,1223 | 0,3249 |
| Leukocytes | Bm2 CB2 MFI | -0,0222 | 0,9041 | 0,9374 |
| Leukocytes | Bm2p CB1 MFI | 0,1941 | 0,2870 | 0,4968 |
| Leukocytes | Bm2p CB2 MFI | -0,0488 | 0,7910 | 0,8789 |
| Leukocytes | Bm3-4 CB1 MFI | 0,2020 | 0,2676 | 0,4790 |
| Leukocytes | Bm3-4 CB2 MFI | 0,0434 | 0,8134 | 0,8915 |
| Leukocytes | Bm5 CB1 MFI | 0,2090 | 0,2511 | 0,4587 |
| Leukocytes | Bm5 CB2 MFI | -0,0957 | 0,6024 | 0,7880 |
| Leukocytes | B Naive CB1 MFI | 0,1868 | 0,3060 | 0,5112 |
| Leukocytes | B Naive CB2 MFI | -0,0649 | 0,7242 | 0,8450 |
| Leukocytes | B Plasma Cells CB1 MFI | 0,2292 | 0,2071 | 0,4177 |
| Leukocytes | B Plasma Cells CB2 MFI | 0,0550 | 0,7650 | 0,8628 |
| Leukocytes | B reg CB1 MFI | 0,1970 | 0,2797 | 0,4917 |
| Leukocytes | B reg CB2 MFI | -0,0275 | 0,8812 | 0,9244 |
| Leukocytes | B Sm CB1 MFI | 0,1523 | 0,4053 | 0,6088 |
| Leukocytes | B Sm CB2 MFI | 0,1094 | 0,5510 | 0,7528 |
| Leukocytes | BTransitional CB1 MFI | 0,2240 | 0,2177 | 0,4296 |
| Leukocytes | BTransitional CB2 MFI | -0,0431 | 0,8149 | 0,8915 |
| Leukocytes | B USm CB1 MFI | 0,2596 | 0,1514 | 0,3566 |
| Leukocytes | B USm CB2 MFI | -0,0915 | 0,6186 | 0,7987 |
| Leukocytes | eBm5 CB1 MFI | 0,2082 | 0,2528 | 0,4587 |
| Leukocytes | eBm5 CB2 MFI | -0,0238 | 0,8970 | 0,9347 |
| Leukocytes | T cell CB1 MFI | 0,3281 | 0,0667 | 0,2544 |
| Leukocytes | T cell CB2 MFI | -0,1846 | 0,3118 | 0,5178 |
| Leukocytes | T CD4 CB1 MFI | 0,2905 | 0,1067 | 0,3065 |
| Leukocytes | T CD4 CB2 MFI | -0,1966 | 0,2809 | 0,4919 |
| Leukocytes | T CD4 Effectors CB1 MFI | 0,3628 | 0,0413 | 0,1977 |
| Leukocytes | T CD4 Effectors CB2 MFI | -0,1540 | 0,4001 | 0,6042 |
| Leukocytes | T CD4 EffectorsMemory CB1 MFI | 0,3809 | 0,0315 | 0,1702 |
| Leukocytes | T CD4 EffectorsMemory CB2 MFI | 0,0631 | 0,7317 | 0,8450 |
| Leukocytes | T CD4 MemoryCentral CB1 MFI | 0,3274 | 0,0674 | 0,2544 |
| Leukocytes | T CD4 MemoryCentral CB2 MFI | -0,0110 | 0,9524 | 0,9718 |
| Leukocytes | T CD4 Naive CB1 MFI | 0,2514 | 0,1652 | 0,3669 |
| Leukocytes | T CD4 Naive CB2 MFI | -0,2649 | 0,1429 | 0,3458 |
| Leukocytes | T CD8 CB1 MFI | 0,3716 | 0,0363 | 0,1794 |
| Leukocytes | T CD8 CB2 MFI | -0,0794 | 0,6659 | 0,8177 |
| Leukocytes | T CD8 Effectors CB1 MFI | 0,3256 | 0,0690 | 0,2544 |
| Leukocytes | T CD8 Effectors CB2 MFI | 0,0713 | 0,6981 | 0,8333 |
| Leukocytes | T CD8 EffectorsMemory CB1 MFI | 0,3375 | 0,0589 | 0,2408 |
| Leukocytes | T CD8 EffectorsMemory CB2 MFI | 0,0958 | 0,6020 | 0,7880 |
| Leukocytes | T CD8 MemoryCentral CB1 MFI | 0,3234 | 0,0710 | 0,2557 |
| Leukocytes | T CD8 MemoryCentral CB2 MFI | 0,0946 | 0,6066 | 0,7889 |
| Leukocytes | T CD8 Naive CB1 MFI | 0,2933 | 0,1033 | 0,3037 |
| Leukocytes | T CD8 Naive CB2 MFI | -0,2082 | 0,2528 | 0,4587 |
| Leukocytes | T reg CB1 MFI | 0,1893 | 0,3649 | 0,5971 |
| Leukocytes | T reg CB2 MFI | 0,0262 | 0,9012 | 0,9316 |
| Lymphocytes | B cell CB1 MFI | 0,4313 | 0,0137 | 0,1254 |
| Lymphocytes | B cell CB2 MFI | -0,2093 | 0,2502 | 0,4587 |
| Lymphocytes | B eUSm CB1 MFI | 0,2717 | 0,1326 | 0,3324 |
| Lymphocytes | B eUSm CB2 MFI | -0,1837 | 0,3143 | 0,5199 |
| Lymphocytes | Bm1 CB1 MFI | 0,4100 | 0,0198 | 0,1408 |
| Lymphocytes | Bm1 CB2 MFI | -0,2115 | 0,2452 | 0,4587 |
| Lymphocytes | Bm2 CB1 MFI | 0,3351 | 0,0608 | 0,2423 |
| Lymphocytes | Bm2 CB2 MFI | -0,2799 | 0,1208 | 0,3249 |
| Lymphocytes | Bm2p CB1 MFI | 0,4018 | 0,0226 | 0,1475 |
| Lymphocytes | Bm2p CB2 MFI | -0,2337 | 0,1980 | 0,4086 |
| Lymphocytes | Bm3-4 CB1 MFI | 0,3761 | 0,0339 | 0,1712 |
| Lymphocytes | Bm3-4 CB2 MFI | -0,1775 | 0,3312 | 0,5370 |
| Lymphocytes | Bm5 CB1 MFI | 0,4214 | 0,0163 | 0,1265 |
| Lymphocytes | Bm5 CB2 MFI | -0,1521 | 0,4058 | 0,6088 |
| Lymphocytes | B Naive CB1 MFI | 0,4214 | 0,0163 | 0,1265 |
| Lymphocytes | B Naive CB2 MFI | -0,2585 | 0,1532 | 0,3567 |
| Lymphocytes | B Plasma Cells CB1 MFI | 0,3787 | 0,0326 | 0,1702 |
| Lymphocytes | B Plasma Cells CB2 MFI | -0,2288 | 0,2079 | 0,4177 |
| Lymphocytes | B reg CB1 MFI | 0,4359 | 0,0126 | 0,1210 |
| Lymphocytes | B reg CB2 MFI | -0,1599 | 0,3822 | 0,5849 |
| Lymphocytes | B Sm CB1 MFI | 0,4784 | 0,0056 | 0,0765 |
| Lymphocytes | B Sm CB2 MFI | -0,1709 | 0,3498 | 0,5534 |
| Lymphocytes | BTransitional CB1 MFI | 0,4500 | 0,0098 | 0,1018 |
| Lymphocytes | BTransitional CB2 MFI | -0,2141 | 0,2393 | 0,4571 |
| Lymphocytes | B USm CB1 MFI | 0,3782 | 0,0328 | 0,1702 |
| Lymphocytes | B USm CB2 MFI | -0,1875 | 0,3041 | 0,5112 |
| Lymphocytes | eBm5 CB1 MFI | 0,4370 | 0,0124 | 0,1210 |
| Lymphocytes | eBm5 CB2 MFI | -0,2366 | 0,1922 | 0,4042 |
| Lymphocytes | T cell CB1 MFI | 0,4053 | 0,0214 | 0,1435 |
| Lymphocytes | T cell CB2 MFI | -0,3811 | 0,0314 | 0,1702 |
| Lymphocytes | T CD4 CB1 MFI | 0,3963 | 0,0247 | 0,1546 |
| Lymphocytes | T CD4 CB2 MFI | -0,4177 | 0,0174 | 0,1282 |
| Lymphocytes | T CD4 Effectors CB1 MFI | 0,4022 | 0,0225 | 0,1475 |
| Lymphocytes | T CD4 Effectors CB2 MFI | -0,4489 | 0,0100 | 0,1018 |
| Lymphocytes | T CD4 EffectorsMemory CB1 MFI | 0,4737 | 0,0062 | 0,0793 |
| Lymphocytes | T CD4 EffectorsMemory CB2 MFI | -0,1883 | 0,3022 | 0,5112 |
| Lymphocytes | T CD4 MemoryCentral CB1 MFI | 0,4251 | 0,0153 | 0,1265 |
| Lymphocytes | T CD4 MemoryCentral CB2 MFI | -0,2014 | 0,2689 | 0,4790 |
| Lymphocytes | T CD4 Naive CB1 MFI | 0,3647 | 0,0402 | 0,1944 |
| Lymphocytes | T CD4 Naive CB2 MFI | -0,4267 | 0,0149 | 0,1265 |
| Lymphocytes | T CD8 CB1 MFI | 0,4229 | 0,0159 | 0,1265 |
| Lymphocytes | T CD8 CB2 MFI | -0,2537 | 0,1612 | 0,3626 |
| Lymphocytes | T CD8 Effectors CB1 MFI | 0,4004 | 0,0232 | 0,1489 |
| Lymphocytes | T CD8 Effectors CB2 MFI | -0,1439 | 0,4320 | 0,6353 |
| Lymphocytes | T CD8 EffectorsMemory CB1 MFI | 0,4505 | 0,0097 | 0,1018 |
| Lymphocytes | T CD8 EffectorsMemory CB2 MFI | -0,1221 | 0,5056 | 0,7044 |
| Lymphocytes | T CD8 MemoryCentral CB1 MFI | 0,4312 | 0,0137 | 0,1254 |
| Lymphocytes | T CD8 MemoryCentral CB2 MFI | -0,0502 | 0,7848 | 0,8742 |
| Lymphocytes | T CD8 Naive CB1 MFI | 0,3610 | 0,0423 | 0,2006 |
| Lymphocytes | T CD8 Naive CB2 MFI | -0,3893 | 0,0276 | 0,1628 |
| Lymphocytes | T reg CB1 MFI | 0,4185 | 0,0374 | 0,2242 |
| Lymphocytes | T reg CB2 MFI | -0,2893 | 0,1607 | 0,3617 |
| neutrophils | B cell CB1 MFI | 0,1665 | 0,3625 | 0,5644 |
| neutrophils | B cell CB2 MFI | 0,0774 | 0,6738 | 0,8217 |
| neutrophils | B eUSm CB1 MFI | 0,3007 | 0,0945 | 0,2872 |
| neutrophils | B eUSm CB2 MFI | 0,0422 | 0,8187 | 0,8915 |
| neutrophils | Bm1 CB1 MFI | 0,1375 | 0,4529 | 0,6575 |
| neutrophils | Bm1 CB2 MFI | -0,0693 | 0,7062 | 0,8408 |
| neutrophils | Bm2 CB1 MFI | 0,2508 | 0,1661 | 0,3669 |
| neutrophils | Bm2 CB2 MFI | 0,0319 | 0,8624 | 0,9131 |
| neutrophils | Bm2p CB1 MFI | 0,1357 | 0,4590 | 0,6599 |
| neutrophils | Bm2p CB2 MFI | -0,0238 | 0,8970 | 0,9347 |
| neutrophils | Bm3-4 CB1 MFI | 0,1430 | 0,4349 | 0,6375 |
| neutrophils | Bm3-4 CB2 MFI | 0,1034 | 0,5733 | 0,7719 |
| neutrophils | Bm5 CB1 MFI | 0,1672 | 0,3603 | 0,5630 |
| neutrophils | Bm5 CB2 MFI | -0,0766 | 0,6767 | 0,8217 |
| neutrophils | B Naive CB1 MFI | 0,1454 | 0,4272 | 0,6321 |
| neutrophils | B Naive CB2 MFI | -0,0009 | 0,9960 | 0,9960 |
| neutrophils | B Plasma Cells CB1 MFI | 0,1927 | 0,2906 | 0,4991 |
| neutrophils | B Plasma Cells CB2 MFI | 0,0997 | 0,5870 | 0,7824 |
| neutrophils | B reg CB1 MFI | 0,1368 | 0,4554 | 0,6589 |
| neutrophils | B reg CB2 MFI | -0,0442 | 0,8102 | 0,8915 |
| neutrophils | B Sm CB1 MFI | 0,0741 | 0,6870 | 0,8288 |
| neutrophils | B Sm CB2 MFI | 0,1592 | 0,3842 | 0,5861 |
| neutrophils | BTransitional CB1 MFI | 0,1619 | 0,3760 | 0,5794 |
| neutrophils | BTransitional CB2 MFI | -0,0363 | 0,8436 | 0,9029 |
| neutrophils | B USm CB1 MFI | 0,2239 | 0,2180 | 0,4296 |
| neutrophils | B USm CB2 MFI | -0,0631 | 0,7316 | 0,8450 |
| neutrophils | eBm5 CB1 MFI | 0,1823 | 0,3181 | 0,5244 |
| neutrophils | eBm5 CB2 MFI | 0,0293 | 0,8734 | 0,9198 |
| neutrophils | T cell CB1 MFI | 0,3394 | 0,0574 | 0,2390 |
| neutrophils | T cell CB2 MFI | -0,0895 | 0,6263 | 0,8006 |
| neutrophils | T CD4 CB1 MFI | 0,3025 | 0,0924 | 0,2847 |
| neutrophils | T CD4 CB2 MFI | -0,0827 | 0,6527 | 0,8170 |
| neutrophils | T CD4 Effectors CB1 MFI | 0,3682 | 0,0381 | 0,1866 |
| neutrophils | T CD4 Effectors CB2 MFI | -0,0576 | 0,7543 | 0,8571 |
| neutrophils | T CD4 EffectorsMemory CB1 MFI | 0,3787 | 0,0326 | 0,1702 |
| neutrophils | T CD4 EffectorsMemory CB2 MFI | 0,1584 | 0,3865 | 0,5876 |
| neutrophils | T CD4 MemoryCentral CB1 MFI | 0,3322 | 0,0632 | 0,2452 |
| neutrophils | T CD4 MemoryCentral CB2 MFI | 0,0796 | 0,6651 | 0,8177 |
| neutrophils | T CD4 Naive CB1 MFI | 0,2705 | 0,1343 | 0,3324 |
| neutrophils | T CD4 Naive CB2 MFI | -0,1691 | 0,3550 | 0,5586 |
| neutrophils | T CD8 CB1 MFI | 0,3777 | 0,0331 | 0,1702 |
| neutrophils | T CD8 CB2 MFI | -0,0633 | 0,7309 | 0,8450 |
| neutrophils | T CD8 Effectors CB1 MFI | 0,3119 | 0,0822 | 0,2688 |
| neutrophils | T CD8 Effectors CB2 MFI | 0,0875 | 0,6341 | 0,8038 |
| neutrophils | T CD8 EffectorsMemory CB1 MFI | 0,3169 | 0,0772 | 0,2593 |
| neutrophils | T CD8 EffectorsMemory CB2 MFI | 0,0939 | 0,6093 | 0,7901 |
| neutrophils | T CD8 MemoryCentral CB1 MFI | 0,3176 | 0,0765 | 0,2589 |
| neutrophils | T CD8 MemoryCentral CB2 MFI | 0,0638 | 0,7286 | 0,8450 |
| neutrophils | T CD8 Naive CB1 MFI | 0,3161 | 0,0780 | 0,2599 |
| neutrophils | T CD8 Naive CB2 MFI | -0,1305 | 0,4764 | 0,6762 |
| neutrophils | T reg CB1 MFI | 0,2348 | 0,2586 | 0,4654 |
| neutrophils | T reg CB2 MFI | 0,1036 | 0,6223 | 0,8616 |
| Platelets | B cell CB1 MFI | 0,1460 | 0,4254 | 0,6321 |
| Platelets | B cell CB2 MFI | 0,0977 | 0,5946 | 0,7867 |
| Platelets | B eUSm CB1 MFI | 0,3746 | 0,0346 | 0,1732 |
| Platelets | B eUSm CB2 MFI | 0,0224 | 0,9033 | 0,9374 |
| Platelets | Bm1 CB1 MFI | 0,2740 | 0,1292 | 0,3324 |
| Platelets | Bm1 CB2 MFI | -0,0198 | 0,9143 | 0,9394 |
| Platelets | Bm2 CB1 MFI | 0,2991 | 0,0963 | 0,2890 |
| Platelets | Bm2 CB2 MFI | 0,0752 | 0,6826 | 0,8257 |
| Platelets | Bm2p CB1 MFI | 0,2064 | 0,2571 | 0,4639 |
| Platelets | Bm2p CB2 MFI | 0,0088 | 0,9619 | 0,9793 |
| Platelets | Bm3-4 CB1 MFI | 0,1913 | 0,2944 | 0,5036 |
| Platelets | Bm3-4 CB2 MFI | 0,1322 | 0,4707 | 0,6703 |
| Platelets | Bm5 CB1 MFI | 0,2144 | 0,2388 | 0,4571 |
| Platelets | Bm5 CB2 MFI | -0,0303 | 0,8694 | 0,9184 |
| Platelets | B Naive CB1 MFI | 0,2120 | 0,2441 | 0,4587 |
| Platelets | B Naive CB2 MFI | 0,0545 | 0,7672 | 0,8631 |
| Platelets | B Plasma Cells CB1 MFI | 0,2346 | 0,1963 | 0,4086 |
| Platelets | B Plasma Cells CB2 MFI | 0,2338 | 0,1978 | 0,4086 |
| Platelets | B reg CB1 MFI | 0,2166 | 0,2339 | 0,4536 |
| Platelets | B reg CB2 MFI | -0,0119 | 0,9484 | 0,9699 |
| Platelets | B Sm CB1 MFI | 0,0990 | 0,5898 | 0,7829 |
| Platelets | B Sm CB2 MFI | 0,1190 | 0,5165 | 0,7159 |
| Platelets | BTransitional CB1 MFI | 0,2557 | 0,1577 | 0,3567 |
| Platelets | BTransitional CB2 MFI | 0,0673 | 0,7144 | 0,8450 |
| Platelets | B USm CB1 MFI | 0,3099 | 0,0843 | 0,2691 |
| Platelets | B USm CB2 MFI | -0,0440 | 0,8110 | 0,8915 |
| Platelets | eBm5 CB1 MFI | 0,2199 | 0,2266 | 0,4415 |
| Platelets | eBm5 CB2 MFI | 0,0581 | 0,7520 | 0,8567 |
| Platelets | T cell CB1 MFI | 0,0713 | 0,6980 | 0,8333 |
| Platelets | T cell CB2 MFI | -0,0735 | 0,6892 | 0,8292 |
| Platelets | T CD4 CB1 MFI | 0,0633 | 0,7309 | 0,8450 |
| Platelets | T CD4 CB2 MFI | -0,0598 | 0,7451 | 0,8536 |
| Platelets | T CD4 Effectors CB1 MFI | 0,0059 | 0,9746 | 0,9829 |
| Platelets | T CD4 Effectors CB2 MFI | -0,0805 | 0,6614 | 0,8177 |
| Platelets | T CD4 EffectorsMemory CB1 MFI | 0,0908 | 0,6212 | 0,7987 |
| Platelets | T CD4 EffectorsMemory CB2 MFI | 0,0334 | 0,8561 | 0,9108 |
| Platelets | T CD4 MemoryCentral CB1 MFI | 0,1031 | 0,5746 | 0,7719 |
| Platelets | T CD4 MemoryCentral CB2 MFI | 0,0523 | 0,7764 | 0,8697 |
| Platelets | T CD4 Naive CB1 MFI | 0,0594 | 0,7467 | 0,8536 |
| Platelets | T CD4 Naive CB2 MFI | 0,0053 | 0,9770 | 0,9829 |
| Platelets | T CD8 CB1 MFI | 0,0787 | 0,6687 | 0,8177 |
| Platelets | T CD8 CB2 MFI | -0,0178 | 0,9230 | 0,9461 |
| Platelets | T CD8 Effectors CB1 MFI | 0,0359 | 0,8455 | 0,9029 |
| Platelets | T CD8 Effectors CB2 MFI | 0,0996 | 0,5877 | 0,7824 |
| Platelets | T CD8 EffectorsMemory CB1 MFI | 0,0798 | 0,6643 | 0,8177 |
| Platelets | T CD8 EffectorsMemory CB2 MFI | 0,0202 | 0,9127 | 0,9394 |
| Platelets | T CD8 MemoryCentral CB1 MFI | 0,0765 | 0,6774 | 0,8217 |
| Platelets | T CD8 MemoryCentral CB2 MFI | -0,0375 | 0,8385 | 0,9027 |
| Platelets | T CD8 Naive CB1 MFI | 0,0624 | 0,7342 | 0,8450 |
| Platelets | T CD8 Naive CB2 MFI | -0,0050 | 0,9785 | 0,9829 |
| Platelets | T reg CB1 MFI | -0,0181 | 0,9316 | 0,9316 |
| Platelets | T reg CB2 MFI | -0,0879 | 0,6760 | 0,8691 |
| SLEDAI | B cell CB1 MFI | -0,6727 | 0,0000 | 0,0022 |
| SLEDAI | B cell CB2 MFI | 0,4098 | 0,0198 | 0,1408 |
| SLEDAI | B eUSm CB1 MFI | -0,5562 | 0,0009 | 0,0213 |
| SLEDAI | B eUSm CB2 MFI | 0,3987 | 0,0238 | 0,1508 |
| SLEDAI | Bm1 CB1 MFI | -0,6907 | 0,0000 | 0,0021 |
| SLEDAI | Bm1 CB2 MFI | 0,3773 | 0,0333 | 0,1702 |
| SLEDAI | Bm2 CB1 MFI | -0,6329 | 0,0001 | 0,0046 |
| SLEDAI | Bm2 CB2 MFI | 0,4758 | 0,0059 | 0,0783 |
| SLEDAI | Bm2p CB1 MFI | -0,6353 | 0,0001 | 0,0046 |
| SLEDAI | Bm2p CB2 MFI | 0,5351 | 0,0016 | 0,0305 |
| SLEDAI | Bm3-4 CB1 MFI | -0,5768 | 0,0005 | 0,0145 |
| SLEDAI | Bm3-4 CB2 MFI | 0,3888 | 0,0279 | 0,1628 |
| SLEDAI | Bm5 CB1 MFI | -0,6801 | 0,0000 | 0,0021 |
| SLEDAI | Bm5 CB2 MFI | 0,3184 | 0,0757 | 0,2586 |
| SLEDAI | B Naive CB1 MFI | -0,6860 | 0,0000 | 0,0021 |
| SLEDAI | B Naive CB2 MFI | 0,4534 | 0,0091 | 0,1018 |
| SLEDAI | B Plasma Cells CB1 MFI | -0,4665 | 0,0071 | 0,0890 |
| SLEDAI | B Plasma Cells CB2 MFI | 0,3815 | 0,0312 | 0,1702 |
| SLEDAI | B reg CB1 MFI | -0,6816 | 0,0000 | 0,0021 |
| SLEDAI | B reg CB2 MFI | 0,4093 | 0,0200 | 0,1408 |
| SLEDAI | B Sm CB1 MFI | -0,5814 | 0,0005 | 0,0136 |
| SLEDAI | B Sm CB2 MFI | 0,3432 | 0,0545 | 0,2290 |
| SLEDAI | BTransitional CB1 MFI | -0,6608 | 0,0000 | 0,0028 |
| SLEDAI | BTransitional CB2 MFI | 0,4888 | 0,0045 | 0,0637 |
| SLEDAI | B USm CB1 MFI | -0,6407 | 0,0001 | 0,0044 |
| SLEDAI | B USm CB2 MFI | 0,3576 | 0,0445 | 0,2042 |
| SLEDAI | eBm5 CB1 MFI | -0,6573 | 0,0000 | 0,0028 |
| SLEDAI | eBm5 CB2 MFI | 0,4525 | 0,0093 | 0,1018 |
| SLEDAI | T cell CB1 MFI | -0,5822 | 0,0005 | 0,0136 |
| SLEDAI | T cell CB2 MFI | 0,4201 | 0,0167 | 0,1272 |
| SLEDAI | T CD4 CB1 MFI | -0,5656 | 0,0007 | 0,0185 |
| SLEDAI | T CD4 CB2 MFI | 0,4947 | 0,0040 | 0,0581 |
| SLEDAI | T CD4 Effectors CB1 MFI | -0,5027 | 0,0034 | 0,0523 |
| SLEDAI | T CD4 Effectors CB2 MFI | 0,5536 | 0,0010 | 0,0217 |
| SLEDAI | T CD4 EffectorsMemory CB1 MFI | -0,5904 | 0,0004 | 0,0130 |
| SLEDAI | T CD4 EffectorsMemory CB2 MFI | 0,1391 | 0,4477 | 0,6520 |
| SLEDAI | T CD4 MemoryCentral CB1 MFI | -0,6077 | 0,0002 | 0,0092 |
| SLEDAI | T CD4 MemoryCentral CB2 MFI | 0,2139 | 0,2397 | 0,4571 |
| SLEDAI | T CD4 Naive CB1 MFI | -0,5344 | 0,0016 | 0,0305 |
| SLEDAI | T CD4 Naive CB2 MFI | 0,4991 | 0,0036 | 0,0545 |
| SLEDAI | T CD8 CB1 MFI | -0,5590 | 0,0009 | 0,0209 |
| SLEDAI | T CD8 CB2 MFI | 0,2844 | 0,1146 | 0,3165 |
| SLEDAI | T CD8 Effectors CB1 MFI | -0,5088 | 0,0029 | 0,0491 |
| SLEDAI | T CD8 Effectors CB2 MFI | 0,1865 | 0,3067 | 0,5112 |
| SLEDAI | T CD8 EffectorsMemory CB1 MFI | -0,5823 | 0,0005 | 0,0136 |
| SLEDAI | T CD8 EffectorsMemory CB2 MFI | 0,1977 | 0,2780 | 0,4906 |
| SLEDAI | T CD8 MemoryCentral CB1 MFI | -0,5943 | 0,0003 | 0,0126 |
| SLEDAI | T CD8 MemoryCentral CB2 MFI | 0,1869 | 0,3057 | 0,5112 |
| SLEDAI | T CD8 Naive CB1 MFI | -0,5346 | 0,0016 | 0,0305 |
| SLEDAI | T CD8 Naive CB2 MFI | 0,4304 | 0,0139 | 0,1254 |
| SLEDAI | T reg CB1 MFI | -0,5510 | 0,0043 | 0,0776 |
| SLEDAI | T reg CB2 MFI | 0,3346 | 0,1021 | 0,3321 |
